# Bacterial and Parasitic Pathogens as Risk Factors for Cancers in the Gastrointestinal Tract: A Review of Current Epidemiological Knowledge

**DOI:** 10.3389/fmicb.2021.790256

**Published:** 2021-12-08

**Authors:** Janneke W. Duijster, Eelco Franz, Jacques Neefjes, Lapo Mughini-Gras

**Affiliations:** ^1^Centre for Infectious Disease Control, National Institute for Public Health and the Environment (RIVM), Bilthoven, Netherlands; ^2^Department of Cell and Chemical Biology, Oncode Institute, Leiden University Medical Center, Leiden, Netherlands; ^3^Institute for Risk Assessment Sciences, Faculty of Veterinary Medicine, Utrecht University, Utrecht, Netherlands

**Keywords:** bacterial infection, parasitic infection, gastrointestinal cancer, epidemiology, oncogenic potential, environmental risk factor

## Abstract

The oncogenic potential of viral infections is well established and documented for many years already. However, the contribution of (commensal) bacteria and parasites to the development and progression of cancers has only recently gained momentum, resulting in a rapid growth of publications on the topic. Indeed, various bacteria and parasites have been suggested to play a role in the development of gastrointestinal cancer in particular. Therefore, an overview of the current epidemiological knowledge on the association between infections with bacteria and parasites and cancers of the gastrointestinal tract is needed. In this review, we summarized the methodological characteristics and main results of epidemiological studies investigating the association of 10 different bacteria (*Bacteroides fragilis*, *Campylobacter* spp., *Clostridium* spp., *Enterococcus faecalis*, *Escherichia coli*, *Fusobacterium nucleatum*, *Porphyromonas gingivalis*, non-typhoidal *Salmonella*, *Salmonella* Typhi, and *Streptococcus* spp.) and three parasites (*Cryptosporidium* spp., *Schistosoma* spp., and *Strongyloides stercoralis*) with gastrointestinal cancer. While the large body of studies based on microbiome sequencing provides valuable insights into the relative abundance of different bacterial taxa in cancer patients as compared to individuals with pre-malignant conditions or healthy controls, more research is needed to fulfill Koch’s postulates, possibly making use of follow-up data, to assess the complex role of bacterial and parasitic infections in cancer epidemiology. Studies incorporating follow-up time between detection of the bacterium or parasite and cancer diagnosis remain valuable as these allow for estimation of cause-effect relationships.

## Introduction

In 2020, an estimated number of over 5 million new cancers of the gastrointestinal (GI) tract were diagnosed globally (Sung et al., 2021). For most of these cancers, the incidence is increasing, mostly as a result of increasing age and welfare characterized by factors such as changing diets and more sedentary lifestyles. Apart from the major risk factors for GI tract cancer that include genetics, age, smoking, alcohol consumption, obesity, and exposure to radiation/chemicals, a potentially carcinogenic role of microorganisms is gaining momentum. Particularly, the rapid evolution of high throughput sequencing as a tool to identify/quantify the composition of the human microbiome, has led to accumulating indications for a role of commensal bacteria such as *Escherichia coli* and *Fusobacterium nucleatum* in cancer development (Chiu and Miller, 2019). Several mechanisms have been described by which bacteria contribute to cancer development, including induction of DNA damage by toxins and manipulation of host cell signaling pathways, thereby affecting cell proliferation, differentiation, apoptosis, and immune signaling (Van Elsland and Neefjes, 2018). Concerning the gastrointestinal tract, the association is best established (both mechanistically and epidemiologically) for *Helicobacter pylori* as causative agent of gastric cancer (GC) and Mucosa-Associated Lymphoid Tissue (MALT) lymphoma (Kuo and Cheng, 2013; Berger et al., 2016). Yet, while substantial laboratory evidence already exists for the role of several pathogens in cancer development, epidemiological data on of the broader (potential) role of (opportunistic) pathogens in GI cancer development is generally dispersed and unclear.

This review paper aims to provide an overview of the current epidemiological knowledge regarding the association between bacteria and parasites and cancers in the GI tract. To this end, we reviewed epidemiological studies reporting on an association between bacterial or parasitic gastrointestinal infections and GI cancers to identify (in)consistencies in their results also in relation to the different study designs.

## Methods

We systematically searched PubMed, Embase, and Web of Science for articles published since 1966, 1946, and 1988, respectively, until April 2021. Details about the search strategy as well as inclusion and exclusion criteria are listed in Supplementary Tables 1, 2. The search was conducted using every possible combination of keywords listed in the categories in Supplementary Table 1, including key words related to malignancies in the GI tract, microorganisms and study design or measurement indicators. We included case-control studies, cohort studies, and cross-sectional studies making use of surveillance data (e.g., bacterial infection records), serological assays or data about presence/abundance of microbial genetic material in human specimens (e.g., tumor tissue, blood, and feces) in relation to GI cancer. The outcome of interest comprised all primary malignancies of the GI, including esophagus, stomach, small intestine, duodenum, colon, rectum, anus, liver, intrahepatic bile ducts, biliary tract, gallbladder, and pancreas. Viruses were not included in this review. Given that the association between *H. pylori* and GC is already extensively reviewed and meta-analyzed, this was excluded from this review. For the same reason, the relation between the microbiome composition at bacterial phylum- or family-level in and GI cancer (i.e., those studies addressing commensal bacterial phyla or families rather than bacterial species) was excluded. Finally, also the relation between the presence or abundance of specific microorganisms during or post-cancer treatment, as well as experimental (*in vivo* or *in vitro*) studies were excluded (Supplementary Table 2). This primary search was supplemented by a search in MedRvix, Google Scholar, and Google for pre-prints of articles and conference abstracts, using the aforementioned inclusion and exclusion criteria (Supplementary Table 2). To ensure literature saturation, we scanned the reference lists of included studies or relevant reviews identified through the search. The search results were exported and unduplicated by EndNote. PROSPERO was screened for ongoing or recently completed systematic reviews about this topic.

In the primary eligibility screening, titles and abstracts were screened against the inclusion criteria, subsequently, (potential) relevant articles were screened based on full text reports. The following data items were extracted from the included articles: first author, year of publication, country/region of study, study period, microorganism and cancer(s) of interest, study design, population size or number of cases and controls, type of test(s) (e.g., serological, culture-confirmed infection, and presence of bacterial DNA) and type of material tested (e.g., blood, tumor tissue, and feces), measurement indicator(s) (e.g., odds ratios and hazard ratios) and main study outcomes. For each of the included studies, the primary outcome was the overall risk estimate of the association between infection and cancer. Further, subgroup estimates (e.g., stratified by gender and age group or follow-up time) were considered secondary outcomes. Characteristics and findings of the included studies were summarized in text and tables and (in)consistencies in study outcomes within and between microorganisms and malignancies were discussed, also in light of the study designs. The definition of search terms and inclusion and exclusion criteria was done by three reviewers (JD, LM-G, and EF). One reviewer (JD) performed the search, screening of abstracts an full-text articles and data extraction, whereas the process thereafter was performed by four reviewers (JD, LMG, EF, and JN).

## Results

**FIGURE 1:**
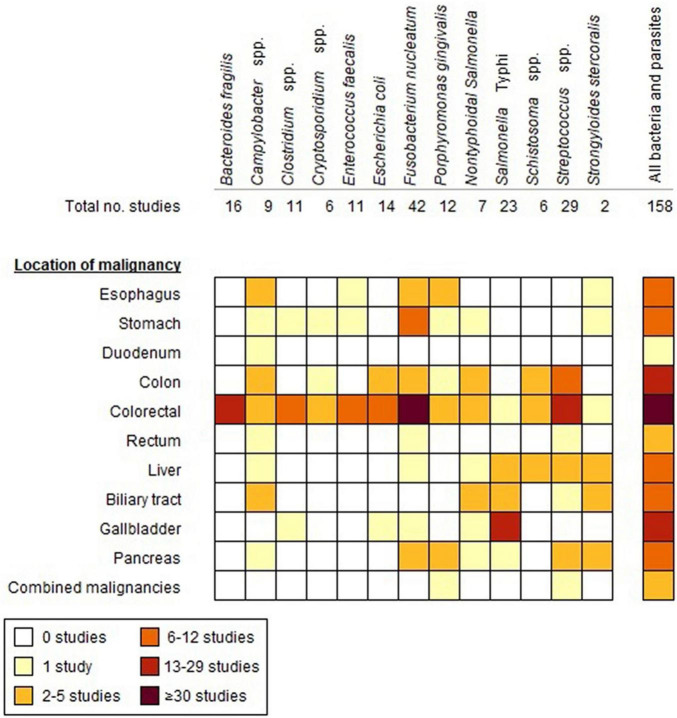
Number of studies included in the review for each bacterium/parasite in relation to the location of GI malignancies.

We identified 4,826 articles by searching the electronic databases (Supplementary Figure 1). After exclusion of duplicates and ineligible articles, 229 articles remained for full-text screening, resulting in 91 included scientific articles. In addition, the manual search [in Google (Scholar)] and screening of the reference lists of eligible studies yielded another 65 scientific articles and two conference abstracts (Supplementary Figure 1). The 158 eligible articles/abstracts cover 13 different micro-organisms, including 10 bacteria and 3 parasites (Figure 1). Most studies had a case-control design (*n* = 101), 33 were cohort studies (22 retrospective and 11 prospective), 23 were cross-sectional studies and 1 was a case series. The majority of the studies were published during the last decade (≥2011: *n* = 116). Study characteristics and main outcomes of the reviewed studies are elaborated in the following paragraphs.

### Bacteroides fragilis

*Bacteroides fragilis* is a commensal bacterium of the gut, subdivided into non-toxigenic *B. fragilis* (NTBF) and enterotoxigenic *B. fragilis* (ETBF). In contrast to the non-harmful NTBF, ETBF is associated with inflammatory bowel disease (IBD) and plays a role in colorectal carcinogenesis by producing pro-inflammatory cytokines and stimulating Wnt signaling (Vacante et al., 2020). Many studies report the presence or abundance of the *Bacteroides* genus (also including NTBF) in the human microbiome in relation to GI cancers. Supplementary Table 3 summarizes the characteristics and main results of 16 studies investigating the association between *B. fragilis* and colorectal cancer (CRC) (Ulger Toprak et al., 2006; Abdulamir et al., 2009; Rahimkhani et al., 2010; Keenan et al., 2014; Boleij et al., 2015; Fukugaiti et al., 2015; Viljoen et al., 2015; Xie et al., 2016; Purcell et al., 2017; Hale et al., 2018; Kwong et al., 2018; Bundgaard-Nielsen et al., 2019; Haghi et al., 2019; Justesen, 2020; Zamani et al., 2020; Ma et al., 2021). Most (*n* = 7) studies found an increased presence of *B. fragilis* and/or ETBF in fecal (*n* = 3) or mucosa/tissue (*n* = 4) samples from CRC patients versus healthy controls, albeit that the prevalences differed substantially between studies (Supplementary Table 3). One study found no significant difference in antibody titers against *B. fragilis* between CRC patients and healthy controls (Abdulamir et al., 2009). No studies were found assessing potential associations between antibody-levels against *B. fragilis*/ETBF and risk of cancer later in life (i.e., with follow-up time). In fact, one study reported similar antibody titers against *B. fragilis* in CRC patients as compared to healthy controls (Abdulamir et al., 2009). *B. fragilis* appeared also significantly enriched in precancerous conditions (Zamani et al., 2020) and was significantly associated with late stages of CRC (Supplementary Table 3; Boleij et al., 2015; Viljoen et al., 2015; Haghi et al., 2019). Whilst the role of the gut microbiome (including the relative abundance of the *Bacteroides* phylum) in other GI cancers is gaining interest, no studies directly assessed the role of *B. fragilis* in GI cancers beyond CRC yet.

### *Campylobacter* spp.

Within the genus *Campylobacter*, two zoonotic species, *Campylobacter jejuni* and *Campylobacter coli*, are the leading causes of gastroenteritis worldwide. A small portion of infections lead to long-term sequelae, including Guillain-Barré syndrome, reactive arthritis, and irritable bowel syndrome (Keithlin et al., 2014). An experimental study in mice showed that infection with a human *C. jejuni* strain induced the development of colorectal tumors and changes in microbial composition through the action of cytolethal distending toxin (CDT). CDT causes double-stranded DNA breaks that may contribute to cancer formation (He et al., 2019). In Supplementary Table 4 nine epidemiological studies are summarized which assess the association between *Campylobacter* and GI cancer (Brauner et al., 2010; Blackett et al., 2013; Wu et al., 2013; Allali et al., 2015; Alexander et al., 2016; Mughini Gras, 2017; De Savornin Lohman et al., 2020; Wang et al., 2020; Wei et al., 2020). In humans, *Campylobacter* spp. is significantly more abundant in the feces of CRC patients compared to healthy controls (Supplementary Table 4; Wu et al., 2013). Similarly, some studies observed significant higher abundance of *Campylobacter* spp. in tumor tissue versus adjacent normal tissue (Allali et al., 2015; Alexander et al., 2016) and tissue samples from cancer patients versus healthy controls (Supplementary Table 4; Wu et al., 2013; Wang et al., 2020). Three cohort studies assessed the association between *Campylobacter* infection and risk of colon cancer (CC), biliary tract cancer or multiple types of cancer later in life. All three studies used a follow-up time of more than 10 years (Supplementary Table 4; Brauner et al., 2010; Mughini Gras, 2017; De Savornin Lohman et al., 2020). None of the studies found a significantly increased risk of digestive cancer after infection compared to the general population, all with standardized incidence ratios (SIRs) below or close to 1. Subgroup analysis in a Dutch cohort study revealed only a significant twofold increased risk of CC when infected with *C. jejuni* or *C. coli* between 40 and 49 years of age (Mughini Gras, 2017). Another species, *Campylobacter concisus*, is considered a human host-adapted species (not isolated from animals), which belongs to the commensal bacteria of the oral cavity and is associated with gingivitis, periodontitis, Barrett’s esophagus (a complication of gastroesophageal reflux disease), gastroenteritis and IBD (Liu et al., 2018). Recently, *C. concisus* has been linked to esophageal adenocarcinoma as this species was found to be significantly more abundant in patients with Barrett’s esophagus, which is a precursor of esophageal cancer (Supplementary Table 4; Yang et al., 2009; Blackett et al., 2013; Kaakoush et al., 2015).

### *Clostridium* spp.

*Clostridium* is a genus of spore-forming bacteria with over 200 species. While the commensal species constitute 10–40% of the total gut microbiome, contributing to gut homeostasis from early infancy onward, the bacteria are also a common cause of food-poisoning and nosocomial infections, particularly in immunocompromised patients (Lopetuso et al., 2013; Yamamoto et al., 2020). Chemotherapeutic cancer treatment alters the composition of the gut microbiome and damages the intestinal mucosa, favoring *Clostridium difficile* colonization and the subsequent production of toxins (toxin A and B) which induce inflammation, tissue damage, and cell death (Di Bella et al., 2016). Whether *Clostridium* also contributes to cancer progression remains elusive. Supplementary Table 5 summarizes 11 studies focusing on *Clostridium* infection and cancer risk (Lu et al., 2004; Rahimkhani et al., 2010; Ahn et al., 2013; Ohigashi et al., 2013; Fukugaiti et al., 2015; Liang et al., 2017, 2021; Hsieh et al., 2018; Kwong et al., 2018; Jahani-Sherafat et al., 2019; Justesen, 2020). Significant increased risk of CRC was observed among individuals with a history of bacteremia with *Clostridium perfringens* or *Clostridium septicum* in a Chinese cohort. However, these numbers were low and the lag-time between bacteremia and cancer diagnosis was not reported (Kwong et al., 2018). Likewise, Justesen (2020) found 22 incidences of CRC among 457 individuals with a history of bacteremia, most of them caused by *C. septicum*. The majority of these cancers (*n* = 20) were diagnosed within one year after the bacteremia (Supplementary Table 5; Justesen, 2020). In another study, significant higher levels of *C. difficile* were detected in feces of CRC patients (prior to treatment) as compared to healthy controls (Supplementary Table 5; Fukugaiti et al., 2015). Similarly, *Clostridium hathewayi*, a relative unknown *Clostridium* species, has been proposed as potential biomarker for early detection of CRC (rather than a causal agent), as it has been associated with overabundance in CRC patients (Supplementary Table 5; Liang et al., 2017, 2021).

### *Cryptosporidium* spp.

*Cryptosporidium* spp. is a protozoan parasite causing enteric infections, generally presenting as a self-limiting watery diarrhea. Infection occurs mainly through the fecal-oral pathway or through consumption/ingestion of contaminated food and water. Over 90% of the infections are caused by the species *Cryptosporidium parvum* and *Cryptosporidium hominis* (Sawant et al., 2020). *Cryptosporidium* is known for its opportunistic behavior in immunocompromised patients (e.g., HIV-patients or individuals receiving oncological treatment) (Kalantari et al., 2020). Experimental studies in mice suggest that *Cryptosporidium* infection can induce intestinal dysplasia (Abdou et al., 2013; El-Kersh et al., 2019). Supplementary Table 6 summarizes the characteristics of six studies assessing the association between *Cryptosporidium* and CRC (*n* = 5), CC (*n* = 1) or GC (*n* = 1) in four different cohorts in Poland, Saudi, Lebanon, and Tunisia (Sulżyc-Bielicka et al., 2007, 2012, 2018; Sanad et al., 2014; Osman et al., 2017; Essid et al., 2018). In a recent meta-analysis summarizing the association between *Cryptosporidium* and cancer, most of the 19 included studies focused on all types of cancer combined (Supplementary Table 7; Kalantari et al., 2020). An over threefold increased risk of *Cryptosporidium* infection was observed among cancer patients (all malignancies) as compared to non-cancer controls (OR 3.30; 95% CI 2.18–4.98), whereas for the digestive cancers only a site-specific estimate for CRC was given (OR 3.70; 95% CI 2.10–6.50), based on four studies (Supplementary Tables 6, 7; Sanad et al., 2014; Osman et al., 2017; Essid et al., 2018; Sulżyc-Bielicka et al., 2018; Kalantari et al., 2020).

### Enterococcus faecalis

*Enterococcus faecalis* is one of the most abundant bacterial species of the human gastrointestinal microbiome playing a major role in maintaining gut homeostasis, particularly in newborns. Some strains are widely used as probiotic in food (supplements) for their health-promoting effects. However, by virtue of its capacity to exchange/acquire virulence factors, *E. faecalis* is frequently associated with severe illness, including bloodstream infections and infective endocarditis (De Almeida et al., 2018). Similarly, the contribution of *E. faecalis* in (colorectal) cancer development is controversial with some studies suggesting cancer promoting capacities while others reported protective effects (De Almeida et al., 2018), likely depending on the presence/absence of specific virulence factors. De Almeida et al. (2019) assessed the oncogenic potential of different *E. faecalis* strains isolated from CRC patients and healthy controls on tumor cell lines. Four strains from controls had an antiproliferative effect on three tumor cell lines, whereas four other strains (two from CRC patients and two from controls) showed no effect (De Almeida et al., 2019). Upon infection of colonic epithelial cells, *E. faecalis* induces the production of reactive oxygen species (ROS), leading to DNA damage and activation of multiple signaling pathways, thereby contributing to cancer (Strickertsson et al., 2013). Regarding epidemiological literature, most of the 11 studies summarized in Supplementary Table 8 compare the (relative) abundance of the bacterium in feces from cancer patients versus healthy controls (Balamurugan et al., 2008; Wang et al., 2012; Corredoira et al., 2015; Viljoen et al., 2015; Zhou et al., 2016; Pericas et al., 2017; Rezasoltani et al., 2018; De Almeida et al., 2019; Geravand et al., 2019; D’asheesh et al., 2021; Shoji et al., 2021). Five studies reported a higher abundance of *E. faecalis* in feces from CRC patients compared to healthy controls or controls with polyps (Supplementary Table 8; Balamurugan et al., 2008; Wang et al., 2012; Rezasoltani et al., 2018; Geravand et al., 2019; D’asheesh et al., 2021), whereas one study reported an opposite pattern (De Almeida et al., 2019). *E. faecalis* was more frequently found in tumor tissue as compared to normal adjacent tissue (Supplementary Table 8; Viljoen et al., 2015; Zhou et al., 2016). Also, patients with a history of *E. faecalis* infective endocarditis showed a higher risk of being diagnosed with CRC (Corredoira et al., 2015; Pericas et al., 2017).

### Escherichia coli

*Escherichia coli* is a commensal bacterium and part of the normal human gut flora. However, within the species, different pathogenic groups exist causing various types of enteric infections (Kaper et al., 2004). Moreover, some *E. coli* strains contribute to CC development through the production of cyclomodulins; toxins that induce DNA double-stranded breaks, chromosomal instability and cell cycle arrest (Dalmasso et al., 2014; Faïs et al., 2018). Particularly, strains harboring the pks genomic island (pks^+^ *E. coli*) produce colibactin, which is subject of research in relation to CC during the last decade (Faïs et al., 2018). Microbiome studies aiming at the identification of bacterial genera and abundance of bacterial species in the gut of CRC patients revealed a significantly reduced (relative) abundance of *Escherichia* in feces of CRC patients compared to healthy controls (Sheng et al., 2019; Tang et al., 2020; Wang Y. et al., 2021). Supplementary Table 9 summarizes the results of 14 studies assessing the link between (mainly) oncogenic/cyclomodulin-producing *E. coli* and CRC (Buc et al., 2013; Bonnet et al., 2014; Kohoutova et al., 2014; Fukugaiti et al., 2015; Xie et al., 2016; Shimpoh et al., 2017; Tsuchiya et al., 2018; Tunsjø et al., 2019; Zarei et al., 2019; Iyadorai et al., 2020; Pleguezuelos-Manzano et al., 2020; Rezasoltani et al., 2020; Tang et al., 2020; Yoshikawa et al., 2020). Multiple studies report overrepresentation of *E. coli* in tumor tissue or fecal samples from CRC patients compared to (paired) normal tissue or fecal samples from healthy controls (Buc et al., 2013; Bonnet et al., 2014; Kohoutova et al., 2014; Rezasoltani et al., 2020). Presence of pks^+^ *E. coli* or specific genes coding for toxins was assessed in seven studies, most of which observed a significantly higher prevalence among CRC patients, though substantial difference in observed prevalences existed between studies (Supplementary Table 9; Buc et al., 2013; Xie et al., 2016; Shimpoh et al., 2017; Tunsjø et al., 2019; Iyadorai et al., 2020; Pleguezuelos-Manzano et al., 2020; Yoshikawa et al., 2020).

### Fusobacterium nucleatum

The anaerobic bacterium *F. nucleatum* is one of the most dominant species of the oral microbiome, displaying opportunistic behavior by causing oral inflammations such as periodontitis and gingivitis. Periodontitis is often caused by a complex of bacteria and is characterized by degradation of the soft tissue and alveolar bone around the teeth and tooth loss (Yang et al., 2014). The last decades, the association between *Fusobacterium nucleatum* and CRC is intensively studied. Multiple mechanisms have been proposed by which *F. nucleatum* promotes carcinogenesis, including the suppression of antitumor activities of the host, promotion of tumor cell proliferation and the induction of a pro-inflammatory tumor microenvironment (Lee et al., 2019). The earliest research assessing the abundance of *Fusobacterium* (at genus level) in relation to CC, dates from 1980, where lower numbers of *Fusobacterium* were isolated from feces of CC patients as compared to healthy controls (Vargo et al., 1980). A large number of microbiome studies have been published during the last decades in which the composition of the major phyla of commensal bacteria, including *Fusobacterium*, was examined in cancer patients versus (healthy) controls. Over 100 epidemiological studies have been identified in the primary search specifically mentioning *F. nucleatum* in the abstract. Supplementary Table 10 summarizes the characteristics and main outcomes of 42 of these epidemiological studies which met the eligibility criteria, most of which comparing *F. nucleatum* presence/abundance in feces from CRC patients versus healthy controls and of tumor tissue versus adjacent normal tissue (Supplementary Table 10; Castellarin et al., 2012; Flanagan et al., 2014; Fukugaiti et al., 2015; Ito et al., 2015; Mira-Pascual et al., 2015; Wang et al., 2016; Xie and Fang, 2016; Yu et al., 2016, 2017; Amitay et al., 2017; Drewes et al., 2017; Eklöf et al., 2017; Liang et al., 2017, 2021; Scott et al., 2017; Suehiro et al., 2017; Yamamura et al., 2017; Yoon et al., 2017; Hale et al., 2018; Hsieh et al., 2018; Kwong et al., 2018; Repass, 2018; Russo et al., 2018; Tsuchiya et al., 2018; Bundgaard-Nielsen et al., 2019; Butt et al., 2019; Chen et al., 2019; De Carvalho et al., 2019; Kageyama et al., 2019; Saito et al., 2019; Tunsjø et al., 2019; Yachida et al., 2019; Zhang et al., 2019; Alkharaan et al., 2020; Boehm et al., 2020; Gantuya et al., 2020; Kashani et al., 2020; Reynolds et al., 2020; Eisele et al., 2021; Kawasaki et al., 2021; Kurt and Yumuk, 2021; Pignatelli et al., 2021). Two meta-analyses published in 2020 reported pooled ORs of 8.3 for detection of *F. nucleatum* in colorectal specimens (feces/mucosa/tissue) and being diagnosed with CRC, and 10.06 for detection of *F. nucleatum* in CRC tissue versus healthy tissue from controls (Supplementary Table 7; Gethings-Behncke et al., 2020; Janati et al., 2020). A similar odds was observed when comparing the presence/abundance of *F. nucleatum* in fecal samples from CRC patients versus healthy controls (OR: 9.01, 95% CI 3.39–23.95; *n* = 7 studies) (Ito et al., 2015; Mira-Pascual et al., 2015; Yu et al., 2016; Drewes et al., 2017; Yoon et al., 2017; Repass, 2018; Gethings-Behncke et al., 2020). The odds of detecting *F. nucleatum* in CRC tissue was also significantly higher than in adjacent normal tissue (OR: 2.42, 95% CI 1.62–3.61; *n* = 7 studies) (Wu et al., 2013; Fukugaiti et al., 2015; Mira-Pascual et al., 2015; Amitay et al., 2017; Eklöf et al., 2017; Suehiro et al., 2017; Yu et al., 2017; Gethings-Behncke et al., 2020). The association between *F. nucleatum* and CRC appeared stronger in Asian populations (OR 12.6; 95% CI 7.2–21.9) compared to American and European populations (OR 5.6; 95% CI 2.8–11.6 and OR 4.6; 95% CI 2.5–8.4, respectively) (Janati et al., 2020). A previous review identified a relatively large difference in observed prevalences of *F. nucleatum* in CRC tissue, ranging from 13 till 75% (Lee et al., 2019). In addition to detection of *F. nucleatum* DNA in fecal or tissue samples, few studies assessed the humoral immune response against *F. nucleatum* in relation to CRC. Significantly higher IgA and IgG titers were measured in serum from CRC patients compared to healthy controls and controls with benign colon diseases (Supplementary Table 10; Wang et al., 2016; Kurt and Yumuk, 2021). Antibody titers were higher for proximal versus distal CRC (Kurt and Yumuk, 2021), though no association between antibody titer and CRC stage was found (Wang et al., 2016). A large European study investigated whether higher *F. nucleatum* antibody titers represent a risk factor for CRC later in life (0.4–8.5 years after serum sampling) (Butt et al., 2019). Antibody responses against ≥2 or ≥3 out of 11 *F. nucleatum* proteins were similar for individuals who were diagnosed with CRC after the serum sampling compared to a control group without cancer diagnosis (17 and 9 versus 21 and 9%, respectively). Also, no difference was observed when stratifying for time of serum analyses and CRC diagnoses (Butt et al., 2019). In addition to CRC, the potential role of *F. nucleatum* in carcinogenesis of other (GI) cancers is gaining momentum. While *F. nucleatum* is an oral bacterium, it is suggested to be translocated hematogenously from the oral cavity to tumor tissues *via* the bloodstream during periodontitis. Here, its adhesion protein Fap2 binds to a carbohydrate (Gal-GalNAc), which is overrepresented in tumor cells of several GI cancers (Hashemi Goradel et al., 2019). Ten studies investigated the association between *F. nucleatum* and other organs in the GI tract, including esophagus (*n* = 4) (Yamamura et al., 2017; Kageyama et al., 2019; Zhang et al., 2019; Kawasaki et al., 2021), stomach (*n* = 6) (Yamamura et al., 2017; Hsieh et al., 2018; Chen et al., 2019; Kageyama et al., 2019; Boehm et al., 2020; Gantuya et al., 2020), pancreas (*n* = 2) (Yamamura et al., 2017; Alkharaan et al., 2020), liver and gallbladder (both *n* = 1) (Yamamura et al., 2017; Tsuchiya et al., 2018). In two studies, presence of *F. nucleatum* was confirmed in (tumor) tissue samples of a large portion of esophageal cancer patients (Zhang et al., 2019; Kawasaki et al., 2021), whereas presence of *F. nucleatum* in saliva of esophageal cancer patients as compared with controls appeared less consistent (Kageyama et al., 2019; Kawasaki et al., 2021). For GC, significant associations between *F. nucleatum* presence/abundance in tumor tissue were observed as compared to adjacent normal tissue and individuals with other underlying medical conditions (Hsieh et al., 2018; Chen et al., 2019; Boehm et al., 2020; Gantuya et al., 2020). For pancreatic cancer, the antibody concentrations against *F. nucleatum* in saliva and plasma were higher in cancer patients than in controls (Alkharaan et al., 2020), with poor evidence of *F. nucleatum* presence in pancreatic tumor tissue (Supplementary Table 10).

### Porphyromonas gingivalis

The Gram-negative bacterium *P. gingivalis* is part of the oral microbiome and is considered a leading cause of severe periodontitis (Mysak et al., 2014; Yang et al., 2014). The (chronic) inflammatory response in periodontitis is associated with several systemic diseases including cardiovascular disease, diabetes, and cancer (Corbella et al., 2018). *P. gingivalis* exhibits a range of virulence factors enabling invasion of (oral) endothelial and epithelial cells, dysregulation of the immune response and inhibition of apoptosis, conditions that favor cancer initiation (Corbella et al., 2018). Recently, Liu et al. (2019) published a literature review about the role of *P. gingivalis* in gastrointestinal cancers, in which they observed that this bacterium is particularly associated with esophageal, colorectal and pancreatic cancers. Generally the presence or abundance of *P. gingivalis* is measured through detection of bacterial DNA in oral/tissue/fecal samples or antibody serum titers (Supplementary Table 11; Ahn et al., 2012; Michaud et al., 2013; Gao et al., 2016, 2018; Sinha et al., 2016; Peters et al., 2017; Fan et al., 2018; Kageyama et al., 2019; Wei et al., 2020; Kawasaki et al., 2021; Pignatelli et al., 2021; Wang X. et al., 2021). Increased orodigestive cancer mortality was reported in individuals with higher serum antibody levels against *P. gingivalis* [relative risk (RR) 2.25; 95% CI 1.23–4.14] although this study also included oral cancers and no site-specific estimates were provided (Ahn et al., 2012). With regard to esophageal cancer, in a Chinese cohort, serum IgA, and IgG titers against *P. gingivalis* were significantly higher in esophageal squamous cell carcinoma (ESCC) patients compared to controls (Supplementary Table 11; Gao et al., 2018). Similarly, the bacterium was detected in 61% of tumor tissues whereas none of the normal esophageal tissue contained bacterial DNA (Gao et al., 2016). This was confirmed in a US cohort, in which the OR for presence of *P. gingivalis* DNA in an oral swab was 1.30 (95% CI 0.96–1.77) for ESCC patients versus controls (Supplementary Table 11; Peters et al., 2017). Moreover, some mechanistical evidence exists for the oncogenic potential of *P. gingivalis* in CRC development (Wang X. et al., 2021), though limited studies assessed the association from an epidemiological perspective (Supplementary Table 11). Ahn et al. (2012) reported a significant excess risk of CRC mortality in patients with periodontal disease (RR: 3.58; 95% CI 1.15–11.16); however, direct links with *P. gingivalis* serum levels were not provided (Ahn et al., 2012). Yet, elevated levels of *P. gingivalis* have been found in feces and tumor tissue samples from CRC patients (Supplementary Table 11; Sinha et al., 2016; Wang X. et al., 2021). Regarding pancreatic cancer, two studies reported a significant association between *P. gingivalis* and pancreatic cancer, either based on its presence in oral samples of cases versus controls (OR 1.60; 95% CI 1.15–2.22) (Fan et al., 2018) or based on high (>200 ng/ml) versus low (≤200 ng/ml) serum antibody titers (OR 2.14; 95% CI 1.05–4.36) (Michaud et al., 2013). However, another study reported a significant reduced abundance of *P. gingivalis* in saliva of pancreatic cancer patients (Supplementary Table 11; Wei et al., 2020). Although, several studies confirmed an association between periodontitis and liver cancer and *P. gingivalis* is suggested to play a role in liver diseases (Liu et al., 2019), no epidemiological studies directly assessing the association between *P. gingivalis* and liver cancer were found.

### Non-typhoidal *Salmonella*

Non-typhoidal *Salmonella* (NTS) is one of the major zoonotic bacteria causing (foodborne) gastrointestinal infections. Most infections are mild and do not require medical care. NTS induces cell transformation of pre-transformed cells by activating the AKT and MAPK pathways through secretion of its effector proteins which was shown for *Salmonella enterica* subsp. *enterica* serotype Typhimurium in an experimental setting using murine gallbladder organoids (Scanu et al., 2015). Seven epidemiological articles were found assessing the association between NTS infection and colorectal (*n* = 3), CC (*n* = 2), biliary tract cancer (*n* = 2), and gallbladder, gastric, liver, and pancreatic cancer (latter four: *n* = 1) (Supplementary Table 12; Kato et al., 2013; Iyer et al., 2016; Lu et al., 2017; Mughini-Gras et al., 2018; De Savornin Lohman et al., 2020; Chang et al., 2021; Duijster et al., 2021). The humoral immune response against *Salmonella* flagellin in CRC patients and individuals with colorectal polyps versus healthy controls was assessed in two different cohorts (Supplementary Table 12; Kato et al., 2013). In both cohorts, significantly higher antibody titers were observed in CRC patients versus controls (Kato et al., 2013). In another study, the *Salmonella* effector protein AvrA, exerting a role in carcinogenesis through activation of the host β-catenin pathway, showed higher abundance in colorectal tumor tissues than in healthy mucosa (Lu et al., 2017). Moreover, two studies focusing exclusively on CC, used a comparable design in which the risk of developing CC after severe salmonellosis was investigated (Mughini-Gras et al., 2018; Duijster et al., 2021). In both studies, the risk of proximal CC after salmonellosis was higher than of distal CC for most subgroups (Supplementary Table 12; Mughini-Gras et al., 2018; Duijster et al., 2021). However, a generally lower risk of CC after NTS infection was found in the Danish cohort as compared with the Dutch cohort. Whilst a significantly increased risk of proximal CC was observed after *S*. Enteritidis infection in the Dutch cohort (SIR: 1.86; 95% CI 1.28–2.61), the Danish study reported only a significant increased risk for serotypes other than Enteritidis and Typhimurium (HR: 1.40; 95% CI 1.03–1.90). In the Dutch cohort, NTS infections reported in people ≥20 years were included in the study, while the risk estimates of the Danish cohort study were based on NTS infections in all age groups. Risks of overall CC and proximal CC were particularly higher in people infected between 20 and 60 years of age in the Dutch cohort [SIRs 1.54 (95% CI 1.09–2.10) and 2.12 (95% CI 1.38–3.09), respectively] (Mughini-Gras et al., 2018). After the age of 60, the incidence of cancer increases substantially due to multiple factors, such as spontaneous mutations (Risques and Kennedy, 2018). Hence, this may dilute the observed effect of NTS in risk estimates including the older age groups. In a Taiwanese cohort, a HR of 1.03 (95% CI 0.72–1.47) was reported for the combined risk of colon and rectum cancer, while an over twofold significantly increased risk of GC was observed (Supplementary Table 12; Chang et al., 2021). In addition, also an increased risk of developing biliary tract cancer after salmonellosis was reported in both a Dutch study (SIR: 1.53; 95% CI 0.70–2.91) and the Taiwanese cohort (HR: 2.23; 95% CI 0.83–6.05), though numbers were low in both studies (De Savornin Lohman et al., 2020; Chang et al., 2021). Iyer et al. (2016) demonstrated the presence of traces of *S*. Typhimurium in 12 out of 26 tumors from Indian gallbladder cancer patients (Supplementary Table 12; Iyer et al., 2016).

### Typhoidal *Salmonella*

*Salmonella* Typhi is a pathogenic bacterium causing typhoid fever mainly in developing countries in Southeast-Asia, South-America, and Africa. Upon invasion of the intestinal mucosa, *S.* Typhi spreads to other organs leading to colonization of the gallbladder and liver in 2–5% of the infections (Di Domenico et al., 2017). Subsequently, an estimated 1–4% of the infected people become chronic asymptomatic carriers, as the bacterium is able to form biofilms on gallstones (Di Domenico et al., 2017). A strong correlation exists between concurrent carriage of chronic *S.* Typhi and gallstones (up to 90% in endemic countries), with the latter considered a major risk factor for GBC (Di Domenico et al., 2017). While GBC is a (relatively) rare malignancy in Western countries, its incidence is higher in countries with endemic *S.* Typhi. Secretion of the typhoid toxin (CDT) is suggested to play a role in establishing long-term infection. Also, CDT induces DNA double-stranded breaks and is involved in activation of the MAPK and AKT pathways, ultimately leading to transformation of pre-transformed cells (Scanu et al., 2015; Di Domenico et al., 2017). Numerous epidemiological studies assessing the association between *S.* Typhi infection and/or chronic carriage and cancer have been published. Supplementary Table 13 summarizes 23 studies, mostly focusing on GBC (Welton et al., 1979; Mellemgaard and Gaarslev, 1988; Caygill et al., 1994; Csendes et al., 1994; Strom et al., 1995; Singh et al., 1996; Nath et al., 1997, 2008; Roa et al., 1999; Dutta et al., 2000; Shukla et al., 2000; Serra et al., 2002; Pandey and Shukla, 2003; Hazrah et al., 2004; Yagyu et al., 2004; Vaishnavi et al., 2005; Sharma et al., 2007; Capoor et al., 2008; Tewari et al., 2010; Safaeian et al., 2011; Scanu et al., 2015; Iyer et al., 2016; Tsuchiya et al., 2018). The design of the studies is often based on observed differences in antibody response against *S.* Typhi between GBC patients and controls or detection rates of the bacterium in gallbladder tissue or bile. A meta-analysis from 2014 reported an overall pooled OR of 4.28 (95% CI 1.84–9.96) (Supplementary Tables 7, 13) (Welton et al., 1979; Caygill et al., 1994; Csendes et al., 1994; Strom et al., 1995; Singh et al., 1996; Nath et al., 1997, 2008; Roa et al., 1999; Dutta et al., 2000; Shukla et al., 2000; Serra et al., 2002; Pandey and Shukla, 2003; Hazrah et al., 2004; Yagyu et al., 2004; Sharma et al., 2007; Tewari et al., 2010; Safaeian et al., 2011; Nagaraja and Eslick, 2014). Results were similar for serological detection (OR: 3.52; 95% CI 2.48–5.00) versus culturing methods (OR: 4.14; 95% CI 2.41–7.12) (Nagaraja and Eslick, 2014). Results were corroborated by a more recent meta-analysis of Koshiol et al. (2016) who also reported slightly higher estimates for culturing methods as compared to antibody detection (Supplementary Tables 7, 13) (Welton et al., 1979; Mellemgaard and Gaarslev, 1988; Csendes et al., 1994; Strom et al., 1995; Roa et al., 1999; Dutta et al., 2000; Shukla et al., 2000; Yagyu et al., 2004; Vaishnavi et al., 2005; Capoor et al., 2008; Nath et al., 2008; Safaeian et al., 2011; Koshiol et al., 2016).

### *Schistosoma* spp.

*Schistosoma* is a genus of trematode worms, commonly referred to as blood flukes, which cause chronic schistosomiasis characterized by intestinal and hepatosplenic disease (Elbaz and Esmat, 2013). The species causing most infections are *Schistosoma japonicum*, endemic in parts of China, East Asia, and the Philippines, *Schistosoma mansoni* mainly occurring in South America and Africa, and *Schistosoma haematobium* present in Africa and the Middle East. Schistosomiasis is associated with high morbidity and mortality levels, particularly in populations with poor sanitation and limited access to safe drinking water (Elbaz and Esmat, 2013). Infection with *S. japonicum* is assigned as a group 2b (possibly carcinogenic to humans) carcinogen by the International Agency for Research on Cancer (IARC) for its role in liver cancer, whereas the carcinogenicity of *S. haematobium* infection in bladder cancer is well established (classified as group 1 by IARC) (International Agency for Research on Cancer [IARC], 1994). Part of the *Schistosoma* eggs become trapped in the gut and liver where they induce inflammations and granulomas, thereby being the major drivers of *Schistosoma* pathogenicity. Moreover, *S. mansoni* soluble egg antigens activate c-Jun (proto-oncogene) and STAT3 (transcription factor), which facilitate the development and progression of HCC tumor formation (Roderfeld et al., 2020). In addition, the inducible nitric oxide synthase present in host cells as part of antimicrobial defense against *S. japonicum*, has a promoting effect on p53 mutations, and tumor formation and progression (Hamid, 2019). Disease severity is generally worse in case of co-infection of a *Schistosoma* species with hepatitis B (HBV) or hepatitis C virus (HCV), which is frequently observed in areas with high incidences of both pathogens (Abruzzi et al., 2016). Chronic infection with HBV/HCV can lead to liver fibrosis/cirrhosis and ultimately liver cancer. The interaction between schistosomiasis and chronic hepatitis infection and their combined contribution to cancer formation remains to be clarified (Abruzzi et al., 2016). With regard to the epidemiological evidence, the earliest documentation dates from the late 1970s with several sources, mainly from China, reporting the transforming potential of *S. japonicum* infections. They describe (geographical) associations between *S. japonicum* infection and (mortality from) CRC (Chen, 2014). It should be mentioned that most of these older studies are difficult to access and hardly describe the methods used. Increased risks of CC of 3.3 and 1.2 were observed among Chinese individuals with a history of *S. japonicum* infection, although the latter was not significant, whereas eightfold and almost fourfold increased risks were observed for rectal cancer and liver cancer, respectively (Xu and Su, 1984; Qiu et al., 2005). Still, literature about *S. japonicum* and cancer is limited to a number of case reports and case series and relatively few larger case-control/cohort studies (Supplementary Table 14; En-Sheng, 1981; Xu and Su, 1984; Iida et al., 1999; Qiu et al., 2005). Similarly, for *S*. *mansoni* a small number of epidemiological studies have been published (Supplementary Table 14; Madbouly et al., 2007; Toda et al., 2015). Particularly for this species, quantification of the cancer risk after infection is more challenging as it remains elusive whether it directly promotes cancer development or indirect through the action of HBV/HCV co-infections (Palumbo, 2007). In the past, HBV/HCV viruses have often been transmitted (*via* contaminated blood, syringes, and needles) during antischistosomal parenteral therapy, particularly in Egypt (Palumbo, 2007). None of the seven HCC patients with a history of schistosomiasis in Brazil had antibodies against HCV, whereas four were tested positive for HBV-antibodies (Toda et al., 2015). In an Egyptian cohort, CRC of patients with *S. mansoni* schistosomiasis occurred at an earlier age and were in a more advanced stage as compared to CRC in patients not associated with schistosomiasis (Supplementary Table 14; Madbouly et al., 2007).

### *Streptococcus* spp.

*Streptococcus gallolyticus* subsp. *gallolyticus* (hereafter referred to as *Sgg*), formerly known as *Streptococcus bovis* biotype I is a low-abundance commensal bacterium of the gut. *Sgg* is associated with infective endocarditis and CRC, which is proposed to be related to its capacity to adhere to collagen (types I and IV), frequently present in damaged heart valves and tumors (Jans and Boleij, 2018). Laboratory evidence shows that *Sgg* promotes tumor development through increasing epithelial cell proliferation and upregulated β-catenin levels (Kumar et al., 2017). Many studies assessed the association between infection with (different types of) *Streptococcus* and endocarditis and CRC, albeit that the magnitude of the observed associations in these studies vary considerably, probably due to differences in study populations and methodology (Supplementary Table 15; Murray and Roberts, 1978; Ruoff et al., 1989; Zarkin et al., 1990; Gonzlez-Quintela et al., 2001; Jean et al., 2004; Corredoira et al., 2005, 2008, 2015; Beck et al., 2008; Abdulamir et al., 2009; Vaska and Faoagali, 2009; Boleij et al., 2010, 2012; Fernández-Ruiz et al., 2010; Rahimkhani et al., 2010; Garza-González et al., 2012; Zammit et al., 2014; Paritsky et al., 2015; Butt et al., 2016, 2017, 2018a,b; Guven et al., 2018; Kale et al., 2018; Kwong et al., 2018; Bundgaard-Nielsen et al., 2019; Justesen, 2020; Sheikh et al., 2020; Wang et al., 2020). A meta-analysis published in 2011, showed that 60% of people with *S. bovis* infections undergoing colonoscopy were diagnosed with CRC, which exceeds the CRC prevalence in the general population (10–25%) (Supplementary Tables 7, 15; Murray and Roberts, 1978; Ruoff et al., 1989; Zarkin et al., 1990; Gonzlez-Quintela et al., 2001; Jean et al., 2004; Corredoira et al., 2005, 2008; Beck et al., 2008; Vaska and Faoagali, 2009; Fernández-Ruiz et al., 2010; Boleij et al., 2011). Similarly, the risk of CRC was significantly increased among people infected with *Sgg* compared to individuals infected with *S. bovis* biotype II [pooled OR: 7.26 (95% CI 3.94–13.36)] (Boleij et al., 2011). Another, more recent, meta-analysis (Supplementary Table 7) reported pooled ORs of 14.54 (95% CI 5.66–37.35) and 2.52 (95% CI 1.14–5.58) for co-occurrence of *S. bovis* infective endocarditis and CRC and fecal carriage and CRC, respectively (Supplementary Table 7; Krishnan and Eslick, 2014). Fecal carriage of *Streptococcus* ranged from 6–46% in individuals with adenomas or CRC to 7–14% in controls (Supplementary Table 15; Jans and Boleij, 2018; Sheikh et al., 2020), while the presence of *Streptococcal* DNA in tumor tissue varies considerably between studies (3–74%) (Jans and Boleij, 2018). *Sgg* infection is more frequently associated with adenomas than carcinomas (Boleij et al., 2011; Pasquereau-Kotula et al., 2018). Whilst the majority of evidence on the association between *Streptococcus* and CRC originates from studies assessing the presence of the bacterium in feces and tumor tissue concomitant with CRC diagnosis as compared with controls, *in vitro* and *in vivo* evidence for a causal relationship between *Streptococcus* and CRC is limited (Jans and Boleij, 2018). Corredoira et al. (2015) found that within a cohort of people with a history of *S. bovis* infective endocarditis 43/54 (80%) of the individuals developed a colorectal neoplasm [(non-)advanced adenoma or carcinoma] several years after the acute infective endocarditis (mean follow-up time 60.6 months) (Supplementary Table 15; Corredoira et al., 2015). More recently, a series of large cohort studies have been published assessing the association from a serological perspective. In these studies, using data from Germany, Spain, and the United States, associations between several *Sgg* antigens and CRC were confirmed. Results from these studies collectively showed that an earlier infection with *Streptococcus*, as detected by antibodies, is a predictor of CRC development, also when the antibodies were detected in blood collected up to 10 years before cancer diagnosis. One study showed a particular stronger association in people aged <65 years (Supplementary Table 15; Butt et al., 2016, 2017, 2018a,b). In contrast to the numerous studies corroborating the association between *Streptococcus* and CRC, only three studies were found describing *Streptococcus* in relation to other GI malignancies (mainly pancreatic and liver cancers), but these concerned (sub)species other than *Sgg* (Supplementary Table 15; Corredoira et al., 2008; Zammit et al., 2014; Kale et al., 2018).

### Strongyloides stercoralis

*Strongyloides stercoralis* is a soil-transmitted helminth mainly occurring in (sub)tropical regions where it is estimated to cause over 100 million infections annually (Buonfrate et al., 2020). Most *S. stercoralis* infected people are asymptomatic or have intermittent symptoms including abdominal pain, diarrhea, respiratory complaints, or skin problems. Individuals with an HTLV-1 (human T-cell lymphotropic virus type 1) coinfection or immunocompromised patients, the autoinfective cycle within the host can result in a hyperinfection, characterized by disseminated colonization affecting numerous organs with a high mortality rate (Buonfrate et al., 2013). This causes colitis-like intestinal symptoms, including ulcer formation, patchy inflammation, submucosal hemorrhage and eosinophilic infiltration, mimicking Crohn’s disease, and ulcerative colitis (Catalano et al., 2017). The mechanism by which *S. stercoralis* contributes to initiation and/or progression of malignancies is unclear. It is also unclear whether this nematode exhibits direct oncogenic potential. It is hypothesized that *S. stercoralis* stimulates replication of HTLV-1, which is known to cause adult T cell leukemia/lymphoma. The association between *S. stercoralis* and HTLV-1 was supported by epidemiological data that which showed an over twofold increased prevalence of *S. stercoralis* among HTLV-1 patients as compared to HTLV-free individuals. However, risk of GI cancers other than liver cancer were not elevated amongst HTLV-infected individuals (Tanaka et al., 2016). Epidemiological data addressing the association between *S. stercoralis* infection and GI cancers is limited to few case-control studies (Supplementary Table 16; Hirata et al., 2007; Tanaka et al., 2016) and a number of case reports addressing strongyloidiasis and GC (Seo et al., 2015) and intestinal cancer (Tomaino et al., 2015; Catalano et al., 2017; Ishikawa et al., 2017; Sava et al., 2020). An almost threefold higher risk of developing cancer in the biliary tract was observed amongst patients with a *S. stercoralis* infection in a Japanese cohort (OR 2.7; 95% CI 1.1–6.3) (Hirata et al., 2007). Yet, these results could not be corroborated in a larger study several years later (Tanaka et al., 2016). Moreover, no significant increased risk of other GI cancers in patients infected with *S. stercoralis* were found (Supplementary Table 16; Hirata et al., 2007; Tanaka et al., 2016). Both studies in Japan, as well as the case reports, assessed the co-occurrence of *S. stercoralis* infection and cancer, which hampers definitive conclusions about the direction of the association. In another study, strongyloidiasis was considered an opportunistic infection, as patients with GI cancer receiving chemotherapy were found to have a 6.7 times higher risk of being infected with *S. stercoralis* as compared to patients receiving treatment for other forms of cancer (OR 6.7; 95% CI 1.3–34.2) (Machado et al., 2008).

## Discussion

**FIGURE 2:**
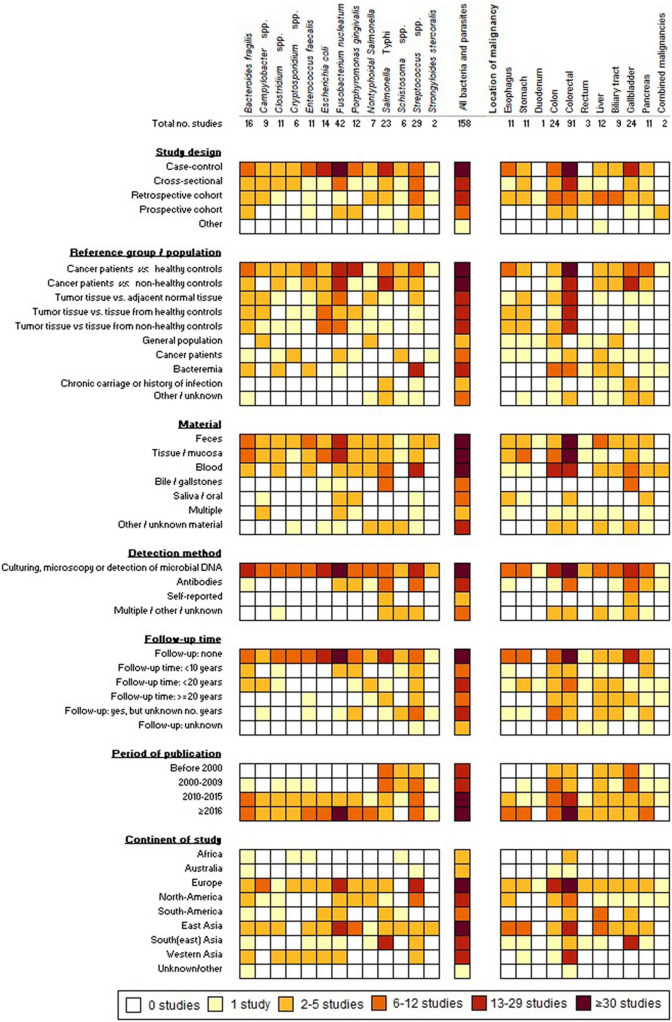
Graphical summary of the 158 included studies.

A growing number of microbial species is being associated with the induction and progression of cancers, partly driven by the development of new diagnostic techniques allowing for a rapid and better understanding of the complex interplay between commensals, pathogens and human cells. For some (mostly pathogenic) microorganisms, the link with cancer has been studied repeatedly in different study populations for many years, whereas for others the scarce evidence is scattered and originates from relatively recent studies. In this review, we provide a comprehensive consideration of epidemiological insights into the association between GI cancers and 13 bacteria and parasites. Figure 2 provides a graphical summary of the study characteristics. Most studies concerned *S.* Typhi, *Streptococcus* spp. and the commensals *F. nucleatum* and *B. fragilis*. Amongst studies comparing the incidence, presence, or abundance of these microorganisms in cancer patients versus healthy controls or the general population, significant positive associations were observed for *B. fragilis* (6/11 studies), *Campylobacter* spp. (4/9), *Clostridium* spp. (4/8), *Cryptosporidium* spp. (3/3), *E. faecalis* (4/7), *E. coli* (5/9), *F. nucleatum* (13/24), *P. gingivalis* (7/11), NTS (3/6), *S.* Typhi (9/10), *Schistosoma* spp. (2/2), *Streptococcus* spp. (11/14), and *S. stercoralis* (1/2) (Supplementary Tables 3–6, 8–16). It is noteworthy that over half of the reviewed studies included less than 50 cancer patients, whilst only 20% of the studies included over 100 cancer patients (Supplementary Tables 3–6, 8–16). For relatively rare malignancies and/or infections with low incidences, the samples sizes of studies are generally rather small, hence, statistical significance is frequently not achieved.

Forty-two studies were identified, that had a study design suitable for estimating the cause and effect relation between bacterial/parasitic infection and GI cancer by inclusion of several years of follow-up (Figure 2). These concerned mostly bacterial infections, including *Streptococcus* (*n* = 16), *Porphyromonas* (*n* = 5), *S.* Typhi (*n* = 5), NTS (*n* = 4), *Campylobacter* (*n* = 4), *Fusobacterium* (*n* = 5), *E. faecalis* (*n* = 2), *B. fragilis* (*n* = 4), and *Clostridium* (*n* = 1) (Figure 1). For pathogenic microorganisms able to cause severe illness, such as NTS, *Campylobacter* spp., and *S. bovis*, often person-level records exist (e.g., from physicians, laboratory diagnoses, or surveillance systems) that provide opportunity for linkage with cancer registry data. Subsequently, this allows for comparison of the cancer incidence among those with a registered history of infection and the cancer incidence in the general population. Maximum follow-up durations of <10 years, 10–20 years, and ≥20 years were reported in 10, 13, and 6 studies, respectively, whereas details about maximum follow-up time were not listed in a substantial fraction of the articles (*n* = 13) (Figure 2). Amongst the studies with follow-up time, five assessed whether seropositivity is a predictor of GI cancer risk later in life (Ahn et al., 2012; Michaud et al., 2013; Butt et al., 2018a,b, 2019). Significantly increased risks of up to twofold were observed for CRC and pancreatic cancer among individuals positive for *S. bovis* and *P. gingivalis* antigens, respectively, in the period up to 10 years before cancer diagnosis (Michaud et al., 2013; Butt et al., 2018a,b). Conversely, seropositivity for *F. nucleatum* was not a predictor of CRC risk later in life (Butt et al., 2019).

For studying the association between commensal bacteria and cancer, a registry-study based on linkage of retrospective databases is often not feasible. However, also the number of studies using a prospective design (with a follow-up period) was limited. Instead, studies focusing on commensal bacteria, including *E. faecalis, F. nucleatum*, and *B. fragilis*, primarily compare the presence or abundance of bacteria in patient material (feces, tumors, and saliva) to the presence/abundance in normal tissue or samples from healthy controls, measured at one point in time, usually resulting from a medical intervention (colonoscopy, endoscopy, and surgery) (Figure 2). These studies are often presented as case-control studies; however, the cross-sectional design without retrospective (risk-factor) data does not allow for assessment of the cause and effect relationship. Tjalsma et al. (2012) described the distinct temporal associations and separate roles of different bacteria with CRC tissue in a “driver-passenger” model (Tjalsma et al., 2012). In this model, bacteria that initiate tumorigenesis by causing DNA damage and malignant transformation of epithelial stem cells are referred to as “driver bacteria.” Subsequently, the induced intestinal alterations favor the proliferation of opportunistic “passenger bacteria” leading to colonization of the tumor microenvironment, thereby outcompeting the original driver bacteria (Tjalsma et al., 2012). Among the bacteria discussed here, *Bacteroides*, *Clostridium* and members of the Enterobacteriaceae family (including *Salmonella* spp.) are considered driver bacteria according to this model, whereas *Fusobacterium* spp. and *Streptococcus* are considered passengers (Tjalsma et al., 2012; Kostic et al., 2013). Although the driver-passenger model was originally developed for CRC, it might apply for other malignancies as well, as *F. nucleatum* and *P. gingivalis* are suggested passengers in pancreatic cancer (Sun et al., 2019).

Twenty-eight studies exclusively made use of control groups with underlying medical conditions, including individuals presenting with gastritis or cholelithiasis, or patients undergoing colonoscopy for gastrointestinal complaints. Although selection of individuals with medical conditions as controls is a convenient option (i.e., with regard to obtaining samples) and can provide insights into the correlation between microbial infection/presence/(relative) abundance and the presence of risk factors (such as cholelithiasis) or pre-malignant conditions (e.g., polyps), the lack of a baseline healthy reference group hampers the accurate assessment of causality (Wacholder et al., 1992).

For all bacteria and parasites, except *B. fragilis*, the association was studied for more than one malignancy, with colon and rectal cancer being the most frequent malignancies analyzed in the literature (CRC *n* = 83, CC *n* = 21, and rectum *n* = 1) (Figure 1). Whilst cancers in colon and rectum are commonly combined into one outcome or risk estimate (i.e., CRC) in epidemiological research given the similarities in anatomical structure; differences in risk factors, etiology and incidence favor the reporting of separate estimates for these subsites (Paschke et al., 2018; Xi et al., 2019). A recent study showed that the relative abundance of commensal bacteria differs at genus level in patients with sigmoid CC as compared to rectal cancer patients (Xi et al., 2019). Similarly, part of the included studies reported separate estimates for proximal versus distal CC and EAC versus ESCC, as these cancers differ in life-style and diet related risk factors (Liu et al., 2014). Particularly for pathogenic bacteria establishing infection in the intestine, the proximal part of the colon (i.e., closest to the ileum) might be of more interest as exposure to bacteria is highest in this part of the colon. For *B. fragilis*, *E. faecalis*, *E. coli*, *F. nucleatum*, and NTS, significant differences in the estimated cancer risk or the observed presence in samples in proximal versus distal CC were reported, though not consistent across bacteria for either of the colon subsites.

Substantial differences were observed in the magnitude of the microorganism-cancer association across different countries/continents for some of the bacteria and parasites. Various factors might underly these inconsistencies, including differences in incidence of both the bacterial/parasitic infections and the specific malignancy, diagnostic performance, and cancer screening programs. With respect to global cancer epidemiology, Asian, African, and Latin American countries generally have higher incidences of esophageal, stomach, liver, and gallbladder cancer, although there is an ongoing displacement toward cancers associated with a higher development index (based on life expectancy, education, and income), such as colorectal and pancreatic cancer, which display higher incidences in countries in Europe, Northern America, and Australia/New Zealand (Sung et al., 2021). Moreover, the cancer inducing and/or promoting capacities sometimes differ between subtypes of the same microorganism, as suggested for *E. coli*, NTS, and *Campylobacter*, consequently leading to differences in cancer risk when global distributions of microorganism subtypes vary (Hendriksen et al., 2011).

For pathogenic bacteria and parasites, the burden of disease is usually based on the incidence, morbidity and possible long-term sequalae, expressed as disability adjusted life years (DALYs) or years of life lost (YLLs) (Pijnacker et al., 2019). According to the IARC, none of the bacteria and only one parasite (*Schistosoma*) discussed in this review is classified as potentially carcinogenic for humans (Boleij et al., 2012). Considering more bacteria and parasites to this IARC list would imply cancer to be recognized as a long-term sequalae of infection and as a consequence a much higher disease burden associated with the specific pathogens. This requires ongoing research to unravel the magnitude and conditions for existence of the association between bacteria/parasites and cancers, and the fraction of GI cancers attributable to these infections.

In order to establish a causal relationship between a microorganism and a disease, the four Koch’s postulates need to be met. Overabundance of the microorganism in people with the disease compared to healthy individuals and isolation of the microorganism from diseased people (either directly or indirectly through serum antibodies) as defined in the first two postulates, are fulfilled in epidemiological studies. However, the last two postulates defining that the microorganism must be able to cause disease in a healthy organism and can be isolated from an experimental host, are not always met and require an experimental design. Hence, this might be a goal for the years to come. Whilst for pathogenic bacteria, measures for prevention of spread (e.g., improved kitchen hygiene, sanitation, etc.) could aid in the prevention of cancers, for commensals prevention is more complex and mainly requires life-style changes related to smoking, eating habits, alcohol consumption, and physical activity, as these factors shape the composition of the microbiome (Matejcic et al., 2017; Savin et al., 2018; Litwinowicz et al., 2020; Aya et al., 2021).

The aim of this review was to provide an comprehensive overview of the volume of research on the several documented associations between bacteria/parasites and GI cancers. However, describing all associations in detail was beyond the scope of this review. Moreover, comparing the magnitude and strength of evidence of the cancer promoting potential between the bacteria and parasites would require thorough quality assessments and weighing of the included articles considering the different study designs and sample sizes, which would be a worthwhile future research objective. As for all literature reviews, relevant articles might have been missed in the search of databases, which may especially be true for those associations addressed only to a limited extent in a few bodies of the scientific literature and not cited in other papers. Likewise, we neither included bacteria nor parasites for which the main body of evidence is indicative of reverse causality, nor bacteria that were exclusively addressed at phylum or family level in, for example, microbiome studies. Last, the review is subject to a degree of publication bias, as studies observing no or limited associations are less likely to be published.

## Conclusion

In conclusion, this review provides a broad overview of the currently existing epidemiological literature about bacterial and parasitic infection/colonization in relation to development and progression of GI malignancies. While the rapidly growing body of studies based on microbiome sequencing provides valuable insights into the relative abundance of different bacterial taxa in cancer patients as compared to individuals with pre-malignant conditions or healthy controls potentially leading to new biomarkers for early detection of cancer, more research is needed to fulfill Koch’s postulates. This involves the use of follow-up data, assessing the complex role of bacteria and parasites in cancer epidemiology and experimental data where isolated infectious species are tested under controlled (laboratory) conditions for their transforming potential. In the future, artificial intelligence could aid in the analysis and transformation of the increasing amount of research data into meaningful risk estimates.

## Author Contributions

JD, LM-G, EF, and JN designed and conceptualized the study. JD performed the literature search and provided a first draft of the review. All authors contributed to writing and revising the manuscript.

## Conflict of Interest

The authors declare that the research was conducted in the absence of any commercial or financial relationships that could be construed as a potential conflict of interest.

## Publisher’s Note

All claims expressed in this article are solely those of the authors and do not necessarily represent those of their affiliated organizations, or those of the publisher, the editors and the reviewers. Any product that may be evaluated in this article, or claim that may be made by its manufacturer, is not guaranteed or endorsed by the publisher.
