# Supplementary material for: Bacterial and Parasitic Pathogens as Risk Factors for Cancers in the Gastrointestinal Tract: A Review of Current Epidemiological Knowledge
Source: Front Microbiol. 2021 Dec 8;12:790256. doi: 10.3389/fmicb.2021.790256 (PMC8692736; doi:10.3389/fmicb.2021.790256)
Supplement: Supplementary file 1 [file Data_Sheet_1.docx]

**Supplementary tables and figure**

**Table S1.** Search terms used in literature search

| **Category 1 † [Title]** | **Category 2† [Title]** | **Category 3 † [Title/Abstract]** | **Category 4†**  **[Title/Abstract]** | **Category 5†**  **[Title]** |
| --- | --- | --- | --- | --- |
| digestive  orodigestive  gastrointestinal  enteric  abdomen  abdominal  esophagus  oesophagus  esophageal  oesophageal  stomach  gastric  duodenum  duodenal  intestine  intestinal  gut  colon  colorectal  bowel  rectosigmoid  rectum  rectal  anus  anal  liver  intrahepatic  hepatic  bile duct  cholangiocarcinoma  gallbladder  gall-bladder  biliary  pancreas  pancreatic | cancer* neoplasm* neoplasia* adenocarcinoma* adenoma* tumor* carcinoma* malignancy malignancies | bacteria*  bacterium  microorganism*  microbe*  microbial  parasite  parasitic | hazard ratio*  HR*  relative risk*  RR*  standardized incidence ratio*  SIR*  standardized mortality ratio*  SMR*  odds  incidence*  Cox  case-control  cohort | COVID  COVID-19  SARS-COV2  virus*  viral  papillomavirus  HPV  Helicobacter  pylori  treatment*  chemotherap*  therapy  therapies  therapeutic  cholecystectomy  hemicolectomy  splenectomy  colectomy  gastrectomy  surgery  surgeries  postoperative  operative  surgical  rat  rats  mouse  mice |

**†** Categories 1-4 combined with ‘AND’, Category 5 ‘AND NOT’.

Table S2. Inclusion and exclusion criteria

| **Inclusion criteria** | **Exclusion criteria** |
| --- | --- |
| - One of the following study designs: - Case-control; - Cohort; - Cross-sectional. - At least one of the following data types: - Surveillance or survey data (e.g. bacterial infection records, self-reported disease); - Serological assays; - Data about presence and/or abundance of microbial genetic material in human specimens (e.g. tumor tissue, blood, feces, saliva) in relation to GI cancer. - Diagnosed malignancy in one of the following organs: - Esophagus; - Stomach; - Small intestine; - Duodenum; - Colon; - Rectum; - Anus; - Liver; - Intrahepatic bile ducts; - Biliary tract; - Gallbladder; - Pancreas. | - Articles exclusively focusing on viruses in relation to cancer. - Articles exclusively focusing on *Helicobacter* *pylori.* - Articles focusing on the broad composition of the microbiome in relation to cancer, i.e. those only addressing bacterial phyla or genera rather than bacterial species* - Articles addressing the association of bacteria/parasites and cancer during or after cancer treatment. - Articles with an experimental design (e.g. in vitro or in vivo studies). - Review articles. |

***** Based on search of title and abstract.

PubMed 1975-2021 (n = 833);
Embase 1972-2021 (n = 2,347);
Web of Science 1994-2021 (n = 1,646)

## Screening

## Included

## Eligibility

## Identification

Google (Scholar) and screening of reference lists of included records (n = 67)

Records after duplicates removed
(n = 2,533 + 69 = 2,602)

Records screened (based on title and abstract)
(n = 2,602)

Records excluded
(n = 2,304)

Full-text articles assessed for eligibility
(n = 298)

Full-text articles excluded
(n = 142)

Studies included in qualitative synthesis (n = 156)

&

(Conference) abstracts included in qualitative synthesis (n = 2)


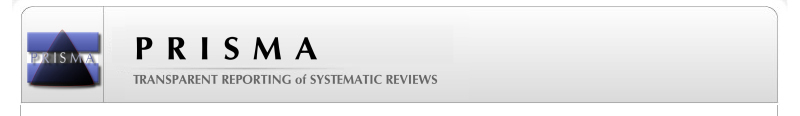


Records identified through database searching

Additional records identified through other sources

**Figure S1.** Prisma flow-diagram of the article selection process.

**Table S3**. Characteristics and main outcomes of epidemiological studies assessing the association between ***Bacteroides fragilis*** and cancer in the gastrointestinal tract.

| **First author, year** | **Country** | **Study period** | **Malignancy/ malignancies** | **Study type** | **Diagnostic method(s)** | **Population size or no. cases & controls** | **Main outcomes** |
| --- | --- | --- | --- | --- | --- | --- | --- |
| Toprak, 2006 [20] | Turkey | *unk* | Colorectal | Case-control | *Bf*: DNA detection in fecal samples using PCR. Cancer: *unk.* | 73 CRC patients, 59 healthy controls. | *Bf* present in fecal samples of 56 (76.7%) CRC patients *vs.* 40 (67.8%) controls (p>0.05). *Bft* gene present in 38% of the CRC patients *vs.* 12% of the controls (RR 4.16; 95%CI 1.39-12.43, p=0.009). |
| Abdulamir, 2009 [11] | Malaysia | 2006-2007 | Colorectal | Case-control | *Bf*: detection of IgG antibodies in blood samples using ELISA, and detection of NF-kB and IL-8 mRNA expression in tissue (tumor, [adjacent] normal) samples using in situ hybridization assay.  Cancer: diagnosis based on colonoscopy. | 50 CRC patients, 14 colorectal adenoma patients, 30 apparently healthy volunteers and 30 controls without colon tumors. | Seroprevalence of *Bf* not higher in CRC patients (0.166 ± 0.013) and adenoma patients (0.178 ± 0.032) as compared with healthy volunteers (0.176 ± 0.014) and controls (0.180 ± 0.020).  NF-kB and IL-8 expression significantly higher in tumor tissue of CRC patients and adenoma patients compared to adjacent normal tissue and tissue from healthy controls (p<0.05). |
| Rahimkhani, 2010 [19] | Iran | *unk* | Colorectal | Case-control | *Bf*: detection in fecal samples using culturing methods.  Cancer: *unk.* | 30 CRC patients, 30 healthy controls. | *Bf* most abundant bacterial species in fecal samples of 8 CRC patients and 2 controls. |
| Keenan, 2014 [16] | *unk* | *unk* | Colorectal | Case-control | *Bf*: DNA and toxin gene detection in fecal samples using PCR. Cancer: *unk.* | 61 CRC patients, 125 healthy controls. | *Bf* detected in 51 (83.6%) of fecal samples from CRC patients *vs.* 114 (91.2%) of healthy controls. ETBF*-*positivity higher in CRC patients (19.6%) *vs.* controls (7.4%) (p=0.087). |
| Boleij, 2015 [12] | USA | 2010-2013 | Colorectal | Case-control | *Bf*: detection of *bft* gene in mucosal samples using PCR.  Cancer: *unk.* | 49 CRC patients, 49 healthy controls. | *Bft* gene present in mucosa of 91.7% proximal and 85.7% of distal CRC tissue or adjacent normal tissue *vs.* 55.5% (proximal) and 53.1% (distal) in controls (significance levels: proximal p=0.04; distal p=0.03). Positive association between *Bft-*positivity and CRC stage (72.7% in early CRC, 100% in late CRC, p=0.09). |
| Fukugaiti, 2015 [14] | Brazil | *unk* | Colorectal | Case-control | *Bf*: detection in fecal samples using PCR. Cancer: diagnosis based on colonoscopy. | 7 CRC patients, 10 healthy controls. | *Bf* detected in feces of 6/7 (85.7%) CRC patients and 6/10 (60.0%) controls. No significant difference in fecal abundance of *Bf* in CRC patients *vs.* controls (4.8 ± 2.6 *vs.* 4.4 ± 2.5 log_10_ no. of copies, p=0.78). |
| Viljoen, 2015 [21] | South-Africa | *unk* | Colorectal | Cross-sectional | *Bf*: detection of *Bf* DNA in tissue samples using qPCR. Cancer: *unk.* | Paired tumor and normal adjacent tissue from 55 CRC patients. Additionally, 18 adenocarcinoma samples. | ETBF present in 14/54 (25.9%) of CRC patients and 15/53 (28.3%) of adjacent normal tissue. Both tumor and normal tissue infected in 71% of ETBF-positive patients. ETBF significantly more present in colon than rectum, no differences between proximal and distal colon. Positive association between ETBF-positivity and CRC stage. |
| Xie, 2016a* [22] | China | 2011-2013 | Colorectal | Case-control | *Bf*: DNA detection in tissue samples using PCR. Cancer: colon/rectum cancer diagnosed by colonoscopy and pathology. | Paired tumor and normal adjacent tissue from 36 CRC patients. Normal tissue from 18 healthy controls. | Significant decreased number of *Bf* in CRC tissue (5.5x10^3 copies/g) *vs.* normal adjacent (4.3x10^4) and healthy control tissue (6.0x10^5). ETBF was significantly increased in CRC tissue compared to healthy normal tissue. |
| Purcell, 2017 [18] | New-Zealand | 2003-2005 | Colorectal | Prospective cohort | *Bf*: *bft* gene detection in tissue samples using PCR. Cancer: *unk.* | 150 patients referred for colonoscopy, 20 of which developed CRC. | ETBF present in tissue samples of 74/150 (49.3%) patients. No significant association between ETBF-positivity and CRC was found. Significant association between ETBF-positivity and low-grade dysplasia (0.0007), tubular adenomas (p 0.027) and serrated polyps (0.007). ETBF presence more likely in lower (distal) parts of colon. |
| Hale, 2018 [15] | USA | *unk* | Colorectal | Cross-sectional | *Bf*: DNA detection in (tumor and adjacent normal) tissue using 16S rRNA gene sequencing and PCR to detect *bft* gene.  Cancer: *unk.* | 83 CRC patients (25 with deficient mismatch repair [dMMR] CRC, 58 with proficient mismatch repair [pMMR] CRC). | *Bf* significantly more abundant in tumor tissue of dMMR CRC patients as compared with normal tissue (p=0.02), not in pMMR CRC patients. No significant difference in prevalence of *bft* gene between dMMR CRC patients and pMMR CRC patients. |
| Kwong, 2018 [17] | China | 2006-2015 | Colorectal | Retrospective cohort | *Bf*: culture-confirmed bacteremia. Cancer: *unk.* | 1,338 *Bf* bacteremia patients, 6,690 matched controls without history of bacteremia. | 42/1,338 (3.1%) of the bacteremia patients developed CRC compared to 73/6,690 (1.1%) of the controls, aHR: 3.85 (95%CI 2.62-5.64; p<0.0001). |
| Bundgaard, 2019 [13] | Denmark | 2002-2010 | Colorectal | Prospective cohort | *Bf*: DNA detection in (tumor and adjacent normal) tissue samples using qPCR.  Cancer: *unk.* | 99 CRC patients, 96 adenoma patients, 104 patients with diverticular disease. | *Bf* detected in 36.4% of the CRC patients. Detection rate lowest for adenoma samples as compared to tumor tissue, adjacent normal and diverticular tissue. Detection rate significantly higher in adjacent normal tissue compared to tumor tissue (p<0.05). |
| Haghi, 2019 [7] | Iran | 2016-2018 | Colorectal | Case-control | *Bf*: detection of *neu* and *bft* genes and enterotoxin in fecal samples using PCR. Cancer: *unk.* | 60 CRC patients, 60 healthy controls. | *neu* gene present in 58.3% CRC patients *vs.* 26.6% controls. *Bft* gene present in 31.6% CRC patients *vs.* 8.3% controls. Prevalence of *Bf* significantly higher in CRC patients *vs.* controls (p<0.05). Positive association between presence of *bft* gene and CRC stage (p<0.05). |
| Justesen, 2020* [9] | Denmark | 2007-2018 | Colorectal | Retrospective cohort | *Bf*: culture confirmed bacteremia (in blood samples).  Cancer: *unk.* | 583 patients with *Bf* bacteremia of which 11 developed CRC. Reference population: ~2 million. | 11/583 (1.9%) individuals with a history of bacteremia caused by *Bf* were diagnosed with CRC, all within 1 year after bacteremia. |
| Zamani, 2020 [8] | Iran | 2015-2017 | Colorectal | Case-control | *Bf*: detection of *Bf* and *bft* gene in mucosal biopsies using rt-PCR. Cancer: histologically confirmed colon/rectum cancer. | Mucosal biopsies from 26 CRC patients and 42 patients with precancerous conditions and from 52 healthy controls. | *Bf* detected in 15/26 (57.7%) CRC patients *vs.* 42/52 (80.8%) healthy controls. *Bft* gene detected in 11/26 (42.3%) CRC patients *vs.* 2/52 (3.8%) healthy controls (OR 18.3; 95%CI 3.7-92.0). |
| Ma, 2021 [10] | Europe | *unk* | Colorectal | Case-control | *Bf*: detection in fecal samples using sequencing.  Cancer: *unk.* | 285 CRC patients, 512 IBD patients, 290 healthy controls. | Abundance of genus-level *Bacteroides* and *Bf* significantly elevated in IBD patients compared to healthy controls and CRC patients. |

*Only abstract available. *unk*: unknown. *Bf*: *Bacteroides fragilis*. (q)PCR: (quantitative) polymerase chain reaction. CRC: colorectal cancer. RR: relative risk. ETBF: enterotoxigenic *B. fragilis.* OR: odds ratio. aHR: adjusted hazard ratio. IBD: inflammatory bowel disease.

**Table S4**. Characteristics and main outcomes of epidemiological studies assessing the association between ***Campylobacter* spp**. and cancer in the gastrointestinal tract.

| **First author, year** | **Country** | **Study period** | **Malignancy/ malignancies** | **Study type** | **Diagnostic method(s)** | **Population size or no. cases & controls** | **Main outcomes** |
| --- | --- | --- | --- | --- | --- | --- | --- |
| Brauner, 2010 [28] | Sweden | 1989-2007 | Esophagus, Stomach, Duodenum, Colon, Rectum + anus, Liver + bile duct | Retrospective cohort | *C. jejuni*: detection in fecal samples using culturing methods.  Cancer: diagnosis according to ICD-9 150-156 codes. | 16,276 individuals with a registered *C. jejuni* infection, of which 3 developed esophageal cancer, 5 GC, none duodenal cancer, 21 CC, 8 rectal or anal cancer, 6 liver or bile duct cancer. Reference population: ~9 million. | Esophageal cancer: SIR 0.99 (95%CI 0.20-2.89); GC SIR: 0.75 (95%CI 0.24-1.76); Duodenal cancer: SIR: 0.00 (95%CI 0.00-2.15); CC SIR: 0.90 (95%CI 0.56-1.38); Rectal or anal cancer: SIR 0.56 (95%CI 0.24-1.10); Liver or bile duct cancer: SIR 1.00 (95%CI 0.37-2.17). Follow-up up to 19 years (median 7.6 years). |
| Blackett, 2013 [27] | UK | *unk* | Esophagus | Case-control | *Campylobacter* spp.: detection in esophageal tissue using culturing methods.  Cancer: diagnosis based on histology. | 30 esophageal cancer patients, 45 Barrett’s esophagus (BE) patients, 37 gastro-esophageal reflux disease (GERD) patients and 39 healthy controls. | *Campylobacter* spp. detected in 3/34 (8.8%) of the cancer patients, 19/45 (42.2%) BE patients, 19/37 (51.4%) GERD patients, and 5/39 (12.8%) controls. Most of the isolated *Campylobacter* were *C. concisus.* Esophageal colonization by *Campylobacter* in GERD and BE patients is likely caused by refluxate (which is reduced/minimal in cancer patients and healthy controls). |
| Wu, 2013 [33] | China | *unk* | Colorectal | Case-control | *Campylobacter* spp.: detection in fecal samples using pyro-sequencing of the 16S rRNA gene V3 region.  Cancer: *unk.* | 19 CRC patients, 20 healthy controls. | *Campylobacter* significantly more abundant in CRC patients *vs.* controls. |
| Allali, 2015 [26] | USA,  Spain | *unk* | Colorectal | Cross-sectional | *Campylobacter* spp.: detection in (tumor) tissue using 16S rRNA gene sequencing. Cancer: *unk.* | Paired tumor and adjacent normal tissue from 90 CRC patients. | *Campylobacter* spp. significantly more abundant in tumor tissue *vs.* adjacent normal tissue of the Spanish cohort and in the adjacent normal tissue of the USA cohort *vs.* the adjacent normal tissue of the Spanish cohort. |
| Alexander, 2016* [25] | UK | *unk* | Colorectal | Cross-sectional | *Campylobacter* spp.: detection in (tumor) tissue using 16S rRNA gene sequencing.  Cancer: *unk.* | Paired tumor and adjacent normal tissue from 46 CRC patients (19 proximal, 11 distal, 16 rectal). | *Campylobacter* spp. detected in tissue from 11/46 (23.9%) of CRC patients. Significant higher abundance of *Campylobacter* spp. in tumor tissue compared to adjacent normal tissue (p=0.007). |
| Mughini-Gras, 2017* [30] | The Netherlands | 2000-2015 | Colon | Retrospective cohort | *Campylobacter* spp: detection in feces, blood, or other samples using culturing methods. Cancer: diagnosis according to ICD-10 C180-C187 codes. | 83 CC patients. Reference population: ~17 million. | Overall risk of CC not significantly higher after *Campylobacter* infection (SIR 1.06; 95%CI 0.84-1.31). Significant higher risk of CC after infection between 40-49 years of age (SIR 2.27; 95%CI 1.17-3.96). |
| De Savornin-Lohman, 2020 [29] | The Netherlands | 2000-2016 | Biliary tract | Retrospective cohort | *Campylobacter* spp: detection in feces, blood, or other samples using culturing methods. Cancer: diagnosis according to ICD-O C239, C240, C242-C244, C248, C249 codes. | 7 biliary tract cancer patients. Reference population: ~17 million. | No increased risk of biliary tract cancer after *Campylobacter* infection (SIR 0.97; 95%CI 0.39–2.00). |
| Wang, 2020 [31] | China | *unk* | Colorectal | Case-control | *Campylobacter* spp.: detection in (tumor) and mucosa using 16S rRNA gene sequencing. Cancer: *unk.* | Tumor, adjacent normal and off-tumor site tissue from 75 CRC patients and mucosa from 26 healthy controls. | Relative abundance of *Campylobacter* spp. significantly higher at tumor, adjacent normal and off-tumor tissue of CRC patients *vs.* healthy controls. |
| Wei, 2020 [32] | China | 2017-2018 | Pancreas | Case-control | *Campylobacter* spp: detection in saliva samples using 16S rRNA gene sequencing. Cancer: histopathologically-confirmed pancreatic adenocarcinoma. | 41 PDAC patients, 69 healthy controls. | Reduced abundance of *Campylobacter* spp. in saliva of PDAC patients *vs.* controls. |

*Only abstract available. ICD-9 or ICD-10: International Classification of Diseases (9^th^ or 10^th^ revision). GC: gastric cancer. CC: colon cancer. ICD(-O): International Classification of Diseases (for Oncology). SIR: standardized incidence ratio.. *Unk*: unknown.

**Table S5.** Characteristics and main outcomes of epidemiological studies assessing the association between ***Clostridium* spp**. and cancer in the gastrointestinal tract.

| **First author, year** | **Country** | **Study period** | **Malignancy/ malignancies** | **Study type** | **Diagnostic method(s)** | **Population size or no. cases & controls** | **Main outcomes** |
| --- | --- | --- | --- | --- | --- | --- | --- |
| Lu, 2004* [42] | China | *unk* | Gallbladder | Cross-sectional | *Clostridium*: detection in GBC tissue using 16S rRNA PCR. Cancer: *unk.* | 46 GBC patients. | 36/46 (78.3%) of tissue samples contained bacterial DNA, including *C. perfringens*. |
| Rahimkhani, 2010 [19] | Iran | *unk* | Colorectal | Case-control | *Clostridium*: detection in fecal samples using culturing methods. Cancer: *unk.* | 30 CRC patients, 30 healthy controls. | *C. perfringens* most abundant bacterial species in fecal samples of 11 CRC patients and 16 controls. |
| Ahn, 2013 [40] | US | 1985-1989 | Colorectal | Case-control | *Clostridium* spp: detection in fecal samples using 16S rRNA sequencing. Cancer: diagnosis based on histology. | 47 CRC patients, 94 healthy controls. | Decreased abundance of *Clostridium* spp. in CRC patients *vs.* controls. |
| Ohigashi, 2013* [43] | Japan | *unk* | Colorectal | Case-control | *Clostridium*: detection in fecal samples using RT-qPCR. Cancer: *unk.* | 93 CRC patients, 23 adenoma patients, 27 healthy controls. | *C. coccoides* group and *C. leptum* subgroup were significantly less abundant in CRC patients *vs.* controls. |
| Fukugaiti, 2015 [14] | Brazil | *unk* | Colorectal | Case-control | *Clostridium*: detection in fecal samples using PCR. Cancer: diagnosis based on colonoscopy. | 7 CRC patients, 10 healthy controls. | *C. difficile* and *C. perfringens* detected 7/7 and 4/7 CRC patients respectively and 8/10 and 8/10 controls respectively. Significant higher abundance of *C. difficile* in CRC patients *vs.* controls (2.5 ± 0.6 *vs.* 1.6 ± 0.8 log_10_ no. of copies, p=0.04). |
| Liang, 2016 [41] | China | 2009-2015 | Colorectal | Case-control | *C. hathewayi*: detection in fecal samples using qPCR. Cancer: *unk.* | 203 CRC patients, 236 healthy controls. | Significant higher abundance of *C. hathewayi* in feces of CRC patients *vs.* controls (p<0.0001). A combination of four bacteria including *Fusobacterium* *nucleatum*, *Bacteroides* *clarus*, *C. hathewayi* and an undefined species could serve as potential biomarker for early detection of CRC. |
| Hsieh, 2018 [44] | Taiwan | *unk* | Stomach | Case-control | *Clostridium* spp.*:* detection of *Clostridium* in gastric biopsies using 16S RNA sequencing.  Cancer: *unk.* | 7 GC patients, 9 gastritis patients, 7 patients with intestinal metaplasia. | *Clostridium* spp. enriched in GC patients *vs.* gastritis patients. *C. colicanis* most frequently detected species. |
| Kwong, 2018 [17] | China | 2006-2015 | Colorectal | Retrospective cohort | *Clostridium*: culture-confirmed bacteremia. Cancer: *unk.* | 13 patients with *C. septicum* bacteremia *vs.* 65 matched controls without bacteremia. 522 patients with *C. perfringens* bacteremia *vs.* 2,610 matched controls. | *C. septicum*: 4/13 (30.8%) of the bacteremia patients developed CRC compared to 1/65 (1.5%) of the controls, aHR: 17.1 (95%CI 1.82-160.0; p=0.013). *C. perfringens*: 11/522 (2.1%) of the bacteremia patients developed CRC compared to 35/2,610 (1.3%) of the controls, aHR 2.29 (95%CI 1.16-4.52, p=0.017). |
| Jahani-Sherafat, 2019 [45] | Iran | 2016-2017 | Colorectal | Cross-sectional | *C. difficile*: detection in colon biopsies by culturing methods and PCR. Cancer: diagnosis based on colonoscopy and pathologic reports. | Tumor tissue and adjacent normal tissue from 30 CRC patients. | *C. difficile* detected in 18/30 (60.0%) of tumor tissues and 6/30 (20.0%) healthy adjacent tissue. |
| Justesen, 2020* [9] | Denmark | 2007-2018 | Colorectal | Retrospective cohort | *Clostridium*.: culture confirmed bacteremia (in blood samples).  Cancer: *unk.* | 457 patients with *Clostridium* spp. bacteremia, of which 167 caused by *C. perfringens* and 53 by *C. septicum*. Reference population: ~2 million. | 22/457 (4.8%) of individuals with a history of bacteremia caused by *Clostridium* spp. were diagnosed with CRC, most of them (n=20) within 1 year. 3/167 (1.8%) *C. perfringens* and 12/53 (22.6%) *C. septicum* bacteremia patients were diagnosed with CRC, all but one *C. septicum* within one year after bacteremia. |
| Liang, 2021 [46] | China | 2009-2014 | Colorectal | Case-control | *C. hathewayi*: detection in fecal samples using PCR. Cancer: diagnosis based on colonoscopic examination, histopathological review. | 210 CRC patients, 115 AA patients, 86 NAA patients, 265 healthy controls. | *Clostridium* useful as fecal diagnostic marker for CRC as compared to *Fusobacterium* *nucleatum* and *Lachnoclostridium* spp. |

*Only abstract available. *Unk:* unknown. PCR: polymerase chain reaction. GBC: gallbladder carcinoma. CRC: colorectal cancer. GC: gastric cancer. aHR; adjusted hazard ratio. AA: advanced adenocarcinoma. NAA: non-advanced adenocarcinoma.

**Table S6**. Characteristics and main outcomes of epidemiological studies assessing the association between ***Cryptosporidium* spp**. and cancer in the gastrointestinal tract.

| **First author, year** | **Country** | **Study period** | **Malignancy/ malignancies** | **Study type** | **Diagnostic method(s)** | **Population size or no. cases & controls** | **Main outcomes** |
| --- | --- | --- | --- | --- | --- | --- | --- |
| Sulżyc-Bielicka, 2007 [52] | Poland | *unk* | Colorectal | Cross-sectional | *Cryptosporidium*: detection in fecal samples using microscopy and microplate assay. Cancer: diagnosis based on colonoscopy. | 55 CRC patients. | 10/55 (18.2%) of the CRC patients tested positive on *Cryptosporidium.* No statistically significant difference in positivity was observed between proximal and distal colon cancer (proximal: 1/12, 8.3%; distal: 9/43, 20.9%). |
| Sulżyc-Bielicka, 2012* [56] | Poland | 2009-2010 | Colorectal | Cross-sectional | *Cryptosporidium*: detection in fecal samples using immune-enzymatic tests. Cancer: *unk.* | 87 CRC patients. | *Cryptosporidium* detected in feces of 11/87 (12.6%) CRC patients. *Cryptosporidium* infection not significantly associated with age, tumor location or cancer stage. |
| Sanad, 2014 [55] | Saudi | 2010-2011 | Colorectal | Case-control | *Cryptosporidium*: detection based on microscopy. Cancer: *unk.* | 20 CRC patients, 42 healthy controls. | 14/20 (70.0%) of the CRC patients *Cryptosporidium*-positive *vs.* 8/42  (19.0%) of the controls. |
| Osman, 2017 [54] | Lebanon | 2012-2013 | Stomach, Colon | Case-control | *Cryptosporidium:* detection in biopsies using 18S rRNA real-time PCR, microscopy, immunofluorescence analysis. Cancer: recently diagnosed colon or stomach intraepithelial neoplasia/adenocarcinoma. | 72 CC patients, 21 GC patients, 125 controls without cancer but with persistent digestive symptoms (non-healthy controls), 44 healthy controls. | PCR-positive CC patients *vs.* healthy controls: OR 11.32 (95%CI 1.44-89.02). PCR-positive colon cancer patients *vs.* non-healthy controls: OR 4.05 (95%CI 1.39-11.79). No PCR-positive samples of GC patients. |
| Essid, 2018 [53] | Tunisia | 2010-2015 | Colorectal | Case-control | *Cryptosporidium*: detection in fecal samples using microscopy and PCR. Cancer: *unk.* | 526 fecal samples, of which 15 from CRC patients, 260 from children, 197 from immunocompromised patients, 54 from myeloma patients. | 5/15 (33.3%) CRC patients tested *Cryptosporidium*-positive *vs.* 37/511 (7.2%) of the non-CRC patients and controls. |
| Sulżyc-Bielicka, 2018 [52] | Poland | 2009-2014 | Colorectal | Case-control | *Cryptosporidium*: antigen detection in fecal samples using antigen-EIA technique. Cancer: diagnosis based on histopathology. | 108 CRC patients, 125 healthy controls. | *Cryptosporidium* infections in cancer patients (prior to oncological treatment) *vs.* controls OR 3.43 (95%CI 1.17-10.07). |

*Only abstract available. *Unk:* unknown. CRC: colorectal cancer. CC: colon cancer. GC: gastric cancer. PCR: polymerase chain reaction. Antigen-EIA: antigen enzyme immunoassay. OR: odds ratio.

**Table S7.** Characteristics and main outcomes of meta-analyses assessing the association between microorganism(s) and cancer in the gastrointestinal tract.

| **First author, year** | **Microorganism** | **Type of study** | **Study period** | **Malignancy/ malignancies*** | **Articles/sources included** | **Main outcomes** |
| --- | --- | --- | --- | --- | --- | --- |
| Boleij, 2011 [204] | *Streptococcus bovis* | SR + M-A | up to Dec 2010 | Colorectal | 52 case reports, 31 case series, of which 11 used for M-A. | Significant increased risk of CRC among patients with *Sgg* infection *vs.* *S. bovis* biotype II-infected persons, pooled OR 7.26 (95% CI 3.94–13.36). Higher CRC occurrence among *S*. bovis IE patients than other *S. bovis* infections (OR 3.72; 95%CI 2.03-6.81). The prevalence of CRC among *Sgg* infected people (33-71%) exceeds the prevalence of CRC in general population (10-25%). |
| Krishnan, 2014 [205] | *Streptococcus bovis* | M-A | up to March 2014 | Colorectal | 9 case-control studies, 39 case series. | Strongest association between S. bovis IE and CRC (OR 14.54; 95%CI 5.66–37.35). OR for *S. bovis* septicemia and CRC: 7.48 (95%CI: 3.10–18.06). OR for *S. bovis* fecal carriage and CRC 2.52 (95%CI: 1.14–5.58). All three with moderate levels of heterogeneity. |
| Nagaraja, 2014 [165] | *Salmonella* Typhi | SR + M-A | up to Nov 2013 | Gallbladder | 15 case-control studies, 2 cohort studies. | Chronic carriage of *S*. Typhi was significantly associated with an increased risk of GBC (pooled OR 4.28; 95%CI 1.84-9.96, p<0.01). Estimated risk was higher based on culturing methods (pooled OR 4.14; 95%CI 2.48-5.00, p<0.01) compared to methods based on detection of antibody titers (pooled OR 3.52; 95%CI 2.48-5.00, p<0.01). Estimated risk was higher when using controls without as reference (pooled OR 5.86; 95%CI 3.84-8.95, p<0.01) *vs.* controls with gallstones (pooled OR 2.71; 95%CI 1.92-3.83, p<0.01). |
| Koshiol, 2016 [166] | *Salmonella* Typhi | M-A | up to Feb 2016 | Liver, Biliary tract, Gallbladder | 18 case-control studies, 4 cohort studies. 14 studies used for M-A. | Odds of detecting higher Vi-antibody titers against *S.* Typhi higher in GBC patients compared to controls (OR 4.0; 95%CI 0.9-18.3). RR of detecting *S.* Typhi in bile or fecal samples was 5.0 (95%CI 2.7-9.3). |
| Gethings-Behncke, 2020 [122] | *Fusobacterium nucleatum* | SR + M-A | up to Jan 2019 | Colorectal | 45 relevant articles identified, of which 18 were used for  M-A. | Higher odds of detecting *Fn* in tissue from CRC patients or polyp patients *vs.* healthy controls (pooled OR 10.06; 95%CI 4.48-22.58, n=6 studies, and pooled OR 1.83; 95%CI 1.07-3.16, n=5 studies respectively). Also higher OR for detecting *Fn* in tumor tissue compared to adjacent normal tissue (OR 2.42; 95%CI 1.62-3.61, n=7 studies). Higher *Fn-*positivity rates in fecal samples from CRC patients *vs.* healthy controls (OR 9.01; 95%CI 3.39-23.95, n=7 studies). |
| Janati, 2020 [123] | *Fusobacterium nucleatum* | SR + M-A | up to Dec 2018 | Colorectal | 24 relevant articles identified, of which 12 were used for M-A. | A significant overall association was found between detection of F. nucleatum in colorectal specimens (fecal, mucosal and/or tissue samples) and CRC, based on data of 12 studies (OR 8.3; 95%CI 5.2-13.0; moderate heterogeneity I^2^ 26.3%, p=0.018). The association appeared stronger for Asian populations (OR 12.6; 95%CI 7.2-21.9) compared to American (OR 5.6; 95%CI 2.8-11.6) and European (OR 4.6; 95%CI 2.5-8.4) populations. |
| Kalantari, 2020 [48] | *Cryptosporidium* spp. | SR + M-A | up to July 2018 | Colorectal | 9 case-control studies, 10 cross-sectional studies. | 4 of the included studies reported site-specific estimates for CRC, the other studies assessed malignancies outside the GI tract or provided a single estimate for all malignancies combined. The pooled OR for CRC was 3.7 (95%CI 2.10–6.50). |

*Malignancies within the gastrointestinal tract, excluding possible malignancies outside the gastrointestinal tract. SR: systematic review. M-A: meta-analysis. CRC: colorectal cancer. GI: gastrointestinal. OR: odds ratio. *Sgg: Streptococcus bovis* biotype I. IE: infective endocarditis. GBC: gallbladder carcinoma. RR: relative risk. *Fn*: *Fusobacterium nucleatum.*

**Table S8**. Characteristics and main outcomes of epidemiological studies assessing the association between ***Enterococcus faecalis*** and cancer in the gastrointestinal tract.

| **First author, year** | **Country** | **Study period** | **Malignancy/ malignancies** | **Study type** | **Diagnostic method(s)** | **Population size or no. cases & controls** | **Main outcomes** |
| --- | --- | --- | --- | --- | --- | --- | --- |
| Balamurugan, 2008 [60] | India | *unk* | Esophagus, Stomach, Colorectal | Case-control | *E. faecalis*: detection in fecal samples using PCR.  Cancer: diagnosis based on colonoscopy or biopsy. | 20 CRC patients, 9 patients with esophageal or gastric cancer, 17 healthy controls. | Increased abundance of *E. faecalis* in feces of CRC patients (0.0500 ± 0.0426) *vs.* healthy controls (0.0106 ± 0.0086). No difference observed between esophageal/gastric cancer patients and controls. |
| Wang, 2012 [67] | China | *unk* | Colorectal | Case-control | *Enterococcus*: detection in fecal samples using pyrosequencing of V3 region of 16S rRNA gene.  Cancer: diagnosis based on histopathology. | 46 CRC patients, 56 healthy controls. | Higher relative abundance of *Enterococcus* (genus level) in CRC patients (2.4%) *vs.* controls (0.1%) (p<0.001). |
| Corredoira, 2015 [61] | Spain | 1988-2014 | Colorectal | Prospective cohort | *Enterococcus* spp: detection in blood samples using culturing methods.  Cancer: diagnosis based on histology. | 1,061 patients with enterococcal bacteremia, of which 36 (3.4%) with endocarditis. | 6/28 (21%) of patients with enterococcal endocarditis developed CRC. During the acute endocarditis episode, 9/18 (50%) patients showed colorectal neoplasm, 4 NAA, 5 AA. |
| Viljoen, 2015 [21] | South-Africa | *unk* | Colorectal | Cross-sectional | *E. faecalis*: detection in tissue samples using qPCR. Cancer: *unk.* | Paired tumor and normal adjacent tissue from 55 CRC patients. Additionally, 18 adenocarcinoma samples. | *E. faecalis* detected in 11/40 (27.5%) tumor tissues and 7/38 (18.4%) normal adjacent tissues. Concurrent detection in both tumor and normal tissue in 5/10 (50.0%) patients. |
| Zhou, 2016 [68] | China | 2012-2014 | Colorectal | Case-control | *E. faecalis*: detection in (tumor and/or healthy) tissue samples using PCR. Cancer: diagnosis based on pathology. | 97 CRC patients, 48 healthy controls. | Higher detection rates of *E. faecalis* in tumor tissue (93/97; 95.9%) *vs.* adjacent normal tissue (91/97; 93.8%) (p<0.05). *E. faecalis* more abundant in CRC patients *vs.* controls. |
| Pericàs, 2017 [64] | Spain | 1979-2015 | Colorectal | Retrospective cohort | *E. faecalis*: *E. faecalis* infective endocarditis according to Duke criteria. Cancer: diagnosis based on colonoscopy. | 154 EFIE patients of which 5 developed CRC, 22 NAA, 5 AA. | Prevalence of CRC over 16-fold higher in studied EFIE cohort (8.2%) compared to Spanish population (0.5%). |
| Rezasoltani, 2018 [65] | Iran | 2015-2017 | Colorectal | Case-control | *E. faecalis*: detection in fecal samples using PCR. Cancer: diagnosis based on colonoscopy. | 87 patients with polyps (n=21 hyperplastic polyp [HP], n=16 sessile serrated polyp [SSA], n=29 tubular adenoma [TA], n=21 [tubu]villous polyp [VP/TVP]), 31 healthy controls. | Higher abundance of *E. faecalis* in feces from TA and VP/TVP patients compared to HP, SSA patients and healthy controls. Higher abundance in patients with proximal polyps (*vs.* distal polyps) and with a higher grade of dysplasia. |
| de Almeida, 2019 [58] | Italy | 2016 | Colorectal | Case-control | *E. faecalis*: detection in fecal samples using culturing methods.  Cancer: *unk.* | 9 CRC patients, 9 healthy controls. | Frequency of *E. faecalis* lower in CRC patients (22%) *vs.* healthy controls (77%). |
| Geravand, 2019 [62] | Iran | *unk* | Colorectal | Case-control | *E. faecalis*: detection in fecal samples using PCR. Cancer: diagnosis based on colonoscopy and positive pathology. | 25 CRC patients, 28 patients with polyps, 24 healthy controls. | Higher abundance of *E. faecalis* in feces from CRC patients (11.2x10^9^) compared to polyp patients (9.4x10^8^) (p=0.002) and healthy controls (9x10^8^) (p=0.001). |
| Hussen, 2020 [63] | Iran | 2014-2019 | Colorectal | Case-control | *E. faecalis*: detection in fecal samples using PCR. Cancer: *unk.* | 300 patients with polyps and CRC, 300 healthy controls. | 2.2-fold higher abundance of *E. faecalis* in CRC patients (1.5x10^9^ CFU/mL) *vs.* healthy controls (8.5x10^8^ CFU/mL) (p=0.0013). |
| Shoji, 2021 [66] | Japan | 2018-2019 | Colorectal | Case-control | *E. faecalis*: detection in fecal samples using PCR and sequencing of 16s rRNA gene. Cancer: diagnosis based on colonoscopy. | 36 CRC patients, 38 healthy controls. | Lower abundance of *E. faecalis* in obese CRC patients *vs.* nonobese CRC patients (p<0.01), opposite pattern in healthy controls. No significant difference in relative abundance of *E. faecalis* across CRC stages. |

*Unk:* unknown. PCR: polymerase chain reaction. CRC: colorectal cancer. NAA: non-advanced adenoma. AA: advanced adenoma. qPCR: (quantitative) polymerase chain reaction. EFIE: *E. faecalis* infective endocarditis. HP: hyperplastic polyp. SSA: sessile serrated polyp. TA: tubular adenoma. (T)VP: (tubu)villous polyp.

**Table S9**. Characteristics and main outcomes of epidemiological studies assessing the association between ***Escherichia coli*** and cancer in the gastrointestinal tract.

| **First author, year** | **Country** | **Study period** | **Malignancy/ malignancies** | **Study type** | **Diagnostic method(s)** | **Population size or no. cases & controls** | **Main outcomes** |
| --- | --- | --- | --- | --- | --- | --- | --- |
| Buc, 2013 [76] | France | 2007-2009 | Colon | Case-control | *E. coli*: detection of specific genes in tissue samples using PCR. Cancer: diagnosis based on pathology. | 38 CC patients, 31 non-cancer controls (with diverticulosis). | Number of *E. coli*-negative samples significantly lower in CC patients (1/38, 2.6%) *vs.* non-cancer controls (6/31, 19.4%) (p=0.04). Prevalence of phylogroup B2 *E. coli* significantly higher in tissue from CC patients (55.3%) *vs.* non-cancer controls (19.3%) (p<0.01). Cyclomodulin-encoding genes (*pks*, *cnf1*, *cdt*) overrepresented in CC patients *vs*. non-cancer controls. |
| Bonnet, 2014 [75] | France | 2007-2010 | Colon | Case-control | *E. coli*: detection in (tumor, normal) tissue using microscopy. Cancer: *unk.* | Paired tissue samples (tumor and adjacent normal) from 50 CC patients, tissue samples from 33 non-cancer controls (with uncomplicated diverticulosis). | Significant increased mucosa-associated *E. coli* in tumor tissue (93%) *vs.* adjacent normal tissue (90%) and non-cancer control tissue (88%) (p=0.01 and p<0.001). Significant more mucosa-internalized *E. coli* in tumors (86%) *vs.* adjacent normal (54%) and non-cancer tissue (48%) (p<0.0002). More *E. coli* in normal mucosa distant from tumor in stage III/IV CC *vs.* stage I (p<0.01). |
| Kohoutova, 2014 [78] | Czech Republic | *unk* | Colorectal | Case-control | *E. coli*: detection in mucosal samples using PCR.  Cancer: diagnosis based on colonoscopy. | 30 CRC patients, 30 colorectal adenoma patients, 20 healthy controls. | Frequency of *E. coli* significantly higher in CRC mucosal samples compared to healthy controls (p<0.001). Statistically higher prevalence of *E. coli* phylogroup B2 in proximal CRC *vs.* distal CRC (p=0.028). A positive association between bacteriocinogeny and CRC stage was observed. |
| Fukugaiti, 2015 [14] | Brazil | *unk* | Colorectal | Case-control | *E. coli*: detection in fecal samples using PCR. Cancer: diagnosis based on colonoscopy. | 7 CRC patients, 10 healthy controls. | *E. coli* detected in feces of 7/7 CRC patients and 10/10 controls. No significant difference in fecal abundance of *E. coli* in CRC patients *vs.* controls (6.7 ± 2.4 *vs.* 6.4 ± 2.5 log_10_ no. of copies, p=0.78). |
| Xie, 2016a* [22] | China | 2011-2013 | Colorectal | Case-control | *E. coli*: *pks+ E. coli* detection in tissue samples using PCR. Cancer: diagnosis based on colonoscopy and pathology. | Paired tumor and adjacent normal tissue from 36 CRC patients. Normal tissue from 18 healthy controls. | Expression of *pks+ E. coli* was significantly higher for tumor tissue (2.96 ± 0.28) compared to healthy control tissue (1.06 ± 0.08) (p<0.001). |
| Shimpoh, 2017 [81] | Japan | 2014-2015 | Colorectal | Case-control | *E. coli: pks+ E. coli* in colonic lavage samples using PCR.  Cancer: diagnosis based on pathology. | Paired colonic lavage and biopsy samples of 13 patients. Colonic lavage samples from 35 CRC patients, 37 colorectal adenoma patients and 26 healthy controls. | *pks+ E. coli* prevalence was 43%, 51% and 46% in CRC, colorectal adenoma and healthy samples respectively. Similarly no significant differences were found in *pks+ E. coli* concentrations among the three groups. |
| Tsuchiya, 2018 [85] | Bolivia,  Chile | 2014-2016 | Gallbladder | Case-control | *E. coli*: detection of microbes in bile using sequencing of V3-V4 region. Cancer: *unk.* | 7 GBC patients, 30 controls with CL. | *E. coli* was detected in bile from both GBC and CL patients. |
| Tunsjø, 2019 [82] | Norway | 2014-2017 | Colorectal | Case-control | *E. coli*: detection of *pks, CNF1, tcpC, astA E. coli* genes in fecal and tissue samples using PCR. Cancer: diagnosis based on colonoscopy. | 25 CRC patients, 25 patients with adenomatous polyps, 22 healthy controls. | *E. coli* toxin genes in 52% of CRC tissues, 27% of polyp tissues and 45% of healthy control tissue (no significant difference). Also no difference in fecal samples. |
| Zarei, 2019 [84] | Iran | 2015-2017 | Colorectal | Case-control | *E. coli*: gene detection in biopsy samples using PCR. Cancer: diagnosis based on colonoscopy. | 40 CRC patients, 40 IBD patients, 40 healthy controls. | *E. coli* isolated from 40/48 (83.8%) CRC tissue samples, 40/51 (78.4%) IBD tissue samples and 40/43 (93.0%) healthy tissue samples. In CRC group, phylogroups B2 and A most prevalent. |
| Iyadorai, 2020 [77] | Malaysia | 2014-2015 | Colorectal | Case-control | *E. coli*: 16S rRNA and *clbB* gene detection in tissues using PCR. Cancer: *unk.* | 18 CRC patients, 23 healthy controls. | *pks+ E. coli* detected in 8/48 (16.7%) of CRC patients *vs.* 1/23 (4.4%) controls. Significantly more CRC tissues *pks+ E. coli*-positive (16/96; 16.7%) compared to control tissues (1/26; 3.8%) (p 0.01). |
| Pleguezuelos-Manzano, 2020 [79] | The Netherlands | *unk* | Colorectal | Case-control | *E. coli*: *SBS-pks* and *ID-pks* signatures using whole-genome sequencing. Cancer: data of metastatic whole-genome cancer from the Hartwig Medical cohort. | 496 CRC metastases, 2,969 metastases of other malignancies. | *SBS-pks* and *ID-pks* positive in 37/496 (7.5%) and 44/496 (8.8%) of CRC metastases *vs.* 12/2,969 (0.4%) and 134/2,969 (4.5%) of other metastases (significantly enriched, p<0.001). |
| Rezasoltani, 2020* [80] | Iran | 2016-2017 | Colorectal | Case-control | *E. coli:* detection in fecal samples using qPCR.  Cancer: *unk.* | 20 CRC patients, 42 patients with adenomatous polyposis, 31 healthy controls. | Higher numbers of *E. coli* in fecal samples from CRC patients and adenomatous polyposis patients *vs.* healthy controls (p<0.015). |
| Tang, 2020 [73] | China | *unk* | Colorectal | Case-control | *E. coli*: detection in fecal samples using genome sequencing and PFGE. Cancer: *unk* | 15 CRC patients, 170 healthy controls (68 preschool children, 87 university students, 15 seniors). | Genomic diversity of sampled *E. coli* populations increased with age group. *E. coli* diversity level significantly lower in CRC patients compared to students and seniors. |
| Yoshikawa, 2020 [83] | Japan | *unk* | Colorectal | Cross-sectional | *E. coli*: detection of colibactin-producing (*clb+*) *E. coli* in tissue samples using PCR. Cancer: adenocarcinoma diagnosis based on histopathology. | 34 colorectal adenoma patients. 450 *E. coli* isolates from tumor samples, 279 *E. coli* isolates from non-tumor tissues. | *clb+ E. coli* present in 22/34 colorectal adenoma patients. Prevalence of *clb+* in isolates from tumor tissue higher compared to non-tumor tissue (tumor: 327/450 [72.7%], non-tumor: 123/279 [44.1%]; p<0.05). Prevalence of *clb+ E. coli* not associated with tumor location. |

*Only abstract available. PCR: polymerase chain reaction. CRC: colorectal cancer. *Unk:* unknown. CC: colon cancer. IBD: inflammatory bowel disease. PFGE: pulsed field gel electrophoresis.

**Table S10**. Characteristics and main outcomes of epidemiological studies assessing the association between ***Fusobacterium nucleatum*** and cancer in the gastrointestinal tract.

| **First author, year** | **Country** | **Study period** | **Malignancy/ malignancies** | **Study type** | **Diagnostic method(s)** | **Population size or no. cases & controls** | **Main outcomes** |
| --- | --- | --- | --- | --- | --- | --- | --- |
| Castellarin, 2012 [89] | Canada | *unk* | Colorectal | Cross-sectional | *Fn*: detection in tissue samples using sequencing. Cancer: *unk.* | Paired tissue samples (tumor and normal tissue) from 11 CRC patients. | 9/11 (81.8%) tissue pairs showed over 2-fold higher abundance in tumor tissue compared to normal tissue. |
| Flanagan, 2014 [90] | Czech Republic, Germany, Ireland | 2008-2010 | Colorectal | Case-control | *Fn*: detection in tissue samples and fecal samples by qPCR. Cancer: diagnosis partially based on colonoscopy. | Tumor tissue from 122 CRC patients, matched normal tissue from 105 patients. Adenoma tissue and matched normal tissue from 52 patients. Fecal samples from 7 CRC patients, 24 adenoma patients and 25 healthy controls. | Relative quantification of *Fn* in tumor tissue significantly higher compared to normal tissue (range 2^-29^ – 2^-6^ in tumor tissue *vs.* 2^-30^ – 2^-14^ in normal tissue). *Fn* more abundant in fecal samples from CRC patients compared to the control groups. |
| Fukugaiti, 2015 [14] | Brazil | *unk* | Colorectal | Case-control | *Fn*: detection in fecal samples using PCR. Cancer: diagnosis based on colonoscopy. | 7 CRC patients, 10 healthy controls. | *Fn* detected in 7/7 CRC patients and 9/10 controls. Significant higher abundance of *Fn* in CRC patients *vs.* controls (6.2 ± 1.5 *vs.* 4.0 ± 1.5 log_10_ no. of copies, p=0.01). |
| Mira-Pascual, 2015 [92] | Spain | *unk* | Colorectal | Case-control | *Fn*: detection in fecal samples using pyro-sequencing of 16S rRNA gene and PCR.  Cancer: diagnosis based on colonoscopy. | 7 CRC patients, 11 patients with tubular adenomas, 10 healthy controls. | Higher abundance of *Fn* in CRC patients *vs.* controls (9/15, 60% *vs.* 2/9, 22%; p<0.07). |
| Ito, 2015 [91] | Japan | 2001-2013 | Colorectal | Case-control | *Fn*: detection in tumor tissue samples using PCR. Cancer: *unk.* | 544 CRC patients, 343 patients with serrated lesions, 122 patients with non-serrated adenomas. | 511/544 (93.9%) of tissue samples from CRC patients *Fn-*positive. *Fn-*positivity significantly higher in CRC patients *vs.* patients with precancerous lesions (p<0.0001). Gradual increase in *Fn* abundance from sigmoid to proximal colon in sessile serrated adenoma patients. |
| Liang, 2016 [41] | China | 2009-2015 | Colorectal | Case-control | *Fn*: detection in fecal samples using qPCR. Cancer: *unk.* | 203 CRC patients, 236 healthy controls. | Relative abundance of *Fn* significantly higher in CRC patients *vs.* healthy controls (p<0.0001). |
| Wang, 2016 [96] | China | June-Dec 2013 | Colorectal | Case-control | *Fn*: IgA and IgG antibody detection in serum samples using indirect whole-cell ELISA. Cancer: diagnosis based on histology. | 258 CRC patients, 150 benign colon disease patients, 200 healthy controls. | IgA and IgG titers significantly higher (p<0.001) in CRC patients *vs.* the control groups.  Anti-*Fn*-IgA: average absorbance ± SD in CRC patients 0.390 ± 0.215; benign colon disease group 0.268 ± 0.158; healthy controls 0.246 ± 0.132.  anti-*Fn*-IgG: average absorbance ± SD in CRC patients 0.362 ± 0.194; benign colon disease group 0.270 ± 0.162; healthy controls 0.262 ± 0.152. |
| Xie, 2016b* [97] | China | 2010-2016 | Colorectal | Case-control | *Fn*: detection in fecal samples using qPCR. Cancer: diagnosis based on endoscopy and pathological findings. | 62 CRC patients (38 early stage, 24 late stage), 38 adenoma patients and 54 healthy controls. | Significant increasing trend in *Fn* abundance from healthy controls, early stage CRC to late stage CRC (p<0.01). |
| Yu, 2016 [98] | China | 2014-2015 | Colorectal | Case-control | *Fn*: detection in tissue samples using 16S rRNA FISH. Cancer: diagnosis based on colonoscopy. | 48 proximal CRC patients, 45 distal CRC patients, 79 patients with traditional adenomas, 40 patients with hyperplastic polyps, 68 patients with serrated polyps, 20 mucosal tissues from healthy controls. | Invasive *Fn* more frequently detected in proximal CRC vs distal CRC (89.6% *vs.* 42.2%, p<0.05). Presence of bacterial biofilms slightly higher in proximal *vs.* distal CRC (52.1% *vs.* 48.9%). |
| Amitay, 2017 [93] | Germany | 2005-2013 | Colorectal | Case-control | *Fn*: detection in fecal samples using 16S rRNA gene analysis, PCR.  Cancer: diagnosis based on colonoscopy. | 46 CRC patients, 113 AA patients, 110 NAA patients, 231 healthy controls. | *Fn* more frequently detected in CRC patients *vs.* other three groups (54.3% *vs.* 23.6-25.1%, p<0.001). Relative abundance higher with increasing CRC stage (p=0.049). |
| Drewes, 2017 [94] | Malaysia | *unk* | Colon | Case-control | *Fn*: detection in tissue samples using 16S rRNA gene amplicon sequencing and FISH (for biofilm quantification).  Cancer: *unk.* | 23 paired tissue samples (tumor and adjacent normal tissue) of proximal CC patients. | Higher abundance of invasive biofilms on proximal CC tumors compared to normal tissue. *Fn* more frequently present in tumors with biofilms (16/17; 94.1%) compared to normal tissue. |
| Eklöf, 2017 [95] | Sweden | 2008-2013 | Colorectal | Case-control | *Fn*: detection in fecal samples using PCR. Cancer: diagnosis based on colonoscopy. | 39 CRC patients, 135 patients with low- and high-grade dysplasia, 66 healthy controls. | *Fn* present in fecal samples of all study participants, although at significantly higher levels in CRC patients compared to dysplasia patients and controls (p<0.001). |
| Scott, 2017* [99] | UK | *unk* | Colon | Case-control | *Fn*.: detection in (tumor) tissue using 16S rRNA gene sequencing.  Cancer: diagnosis based on histology. | 17 CC patients, 37 patients with suspected appendicitis. | Higher abundance of *Fn* in appendixes of CC patients compared to adjacent normal tissue (p=0.059). |
| Suehiro, 2017 [100] | Japan | *unk* | Colorectal | Case-control | *Fn*: detection in fecal samples using PCR. Cancer: diagnosis based on colonoscopy. | 158 CRC patients, 19 patients with AA or carcinoma *in situ,* 11 NAA patients and 60 healthy controls. | Significant higher abundance of *Fn* in CRC patients (median copy number: 317 *vs.* control group 17.5; p<0.0001). |
| Yamamura, 2017 [101] | Japan | *unk* | Esophagus, Stomach, Colorectal, Liver, Pancreas | Cross-sectional | *Fn:* detection in tissue samples using qPCR. Cancer: histopathological cancer confirmation. | 20 paired tissue samples (tumor and adjacent normal tissue) for each cancer included. | *Fn* DNA detection: Esophagus: 4/20 tumor tissue; 1/20 normal tissue. Stomach: 2/20 tumor tissue; 0/20 normal tissue. Colorectal: 9/20 tumor tissue; 8/20 tumor tissue. Liver and pancreatic tissues: no DNA detected in tumor nor normal tissues. |
| Yoon, 2017 [102] | Korea | *unk* | Colorectal | Case-control | *Fn*: detection in mucosal tissue samples using 16S rRNA gene pyrosequencing. Cancer: *unk.* | 6 CRC patients, 6 patients with conventional adenoma, 6 patients with sessile serrated adenoma and 6 healthy controls. | *Fn* not detected in samples from CRC patients. Diversity of mucosal communities lowest in CRC patients as compared to other three groups. |
| Yu, 2017 [103] | China, Denmark | *unk* | Colorectal | Case-control | *Fn*: detection in fecal samples using metagenomic sequencing and qPCR. Cancer: diagnosis based on colonoscopy. | Chinese cohort1: 74 CRC patients, 54 controls. Chinese cohort2: 47 CRC patients, 109 controls. Denmark: 16 CRC patients, 24 controls. | *Fn* enriched in fecal samples of CRC patients. *Fn* gene present in 4/109 (3.7%) control samples, potential suitable marker for CRC. |
| Hale, 2018 [15] | USA | *unk* | Colorectal | Cross-sectional | *Fn*: detection in (tumor and adjacent normal) tissue using 16S rRNA gene sequencing  Cancer: *unk.* | 83 CRC patients (25 with deficient mismatch repair [dMMR] CRC, 58 with proficient mismatch repair [pMMR] CRC). | *Fn* significantly more abundant in tumor tissue of dMMR CRC patients as compared with normal tissue (p=0.03), not in pMMR CRC patients. Abundance of *Fusobacterium* spp. more frequently observed in proximal *vs.* distal CC. |
| Hsieh, 2018 [44] | Taiwan | *unk* | Stomach | Case-control | *Fn*: detection in tissue samples using 16S rRNA gene sequencing. Cancer: *unk.* | 11 GC patients, 9 gastritis patients, 7 intestinal metaplasia patients. | *Fn* significantly enriched in gastric tumor tissue compared to gastritis tissue (p<0.01). |
| Kwong, 2018 [17] | China | 2006-2015 | Colorectal | Retrospective cohort | *Fn*: culture-confirmed bacteremia.  Cancer: *unk.* | 79 *Fn* bacteremia patients, 395 matched controls without history of bacteremia. | 4/79 (5.1%) of the bacteremia patients developed CRC compared to 4/395 (1.0%) of the controls, aHR: 6.89 (95%CI 1.70-27.9; p<0.007). |
| Repass, 2018 [105] | USA | *unk* | Colorectal | Cross-sectional | *Fn*: detection in tissue samples using sequencing and PCR. Cancer: *unk.* | 16 CRC tissue samples, 10 adjacent normal tissue samples. | *Fn* present in 25% of CRC samples *vs.* 15% and 0% of adjacent normal and matched normal tissue respectively. |
| Russo, 2018 [106] | Italy | 2015-2016 | Colorectal | Case-control | *Fn*: detection in saliva, feces and tumor tissue samples using 16S rRNA gene sequencing and qPCR. Cancer: diagnosis confirmed by histological analysis. | 10 CRC patients, 10 healthy controls. | No significant difference in observed *Fn* abundance in fecal samples of CRC patients *vs.* controls. Higher abundance in saliva compared to feces in both CRC patients (p<0.01) and controls (p<0.002). |
| Tsuchiya, 2018 [85] | Bolivia,  Chile | 2014-2016 | Gallbladder | Case-control | *Fn*: detection of microbes in bile using sequencing of V3-V4 region. Cancer: *unk.* | 7 GBC patients, 30 controls with CL. | *Fn* detected in bile of GBC patients. |
| Bundgaard, 2019 [13] | Denmark | 2002-2010 | Colorectal | Prospective cohort | *Fn*: detection in (tumor and adjacent normal) tissue samples using qPCR.  Cancer: *unk.* | 99 CRC patients, 96 adenoma patients, 104 patients with diverticular disease. | *Fn* detected in 29.3% of the tumor samples. Detection rate lowest for adenoma samples as compared to tumor tissue, adjacent normal and diverticular tissue. |
| Butt, 2019 [104] | 10 European countries | 1992-2000 | Colorectal | Case-control | *Fn*: detection of antibodies against 11 *Fn* proteins in serum using multiplex serology. Cancer: diagnosis according to ICD-10 C18-C20 codes. | 485 CRC patients (samples drawn 0.4-8.5 years before CRC diagnosis), 485 healthy controls. | Positivity to: ≥1 protein in 47% of patients *vs.* 53% of controls (OR 0.81; 95%CI 0.62-1.06). ≥2 proteins: 17% patients *vs.* 21% controls.  ≥3 proteins: 9% patients and 9% controls. Positivity to a either single or multiple *Fn* proteins not associated with higher cancer risk. |
| Chen, 2019 [107] | China | 2012-2014 | Stomach | Cross-sectional | *Fn*: detection in tissue samples using 16S rRNA gene sequencing. Cancer: *unk.* | 62 paired GC tissue samples (tumor tissue and adjacent normal tissue). | Relative abundance of *Fn* in tumor tissue significantly higher compared to adjacent normal tissue (0.257% *vs.* 0.041%) (p<0.001). |
| de Carvalho, 2019 [108] | Brazil | 2008-2015 | Colorectal | Prospective cohort | *Fn*: detection in (tumor and adjacent normal) tissue using 16S rRNA gene sequencing. Cancer: *unk.* | 152 CRC patients. | *Fn* present in 35/152 (23.0%) of the tumor tissue samples and 6/57 (10.5%) of the adjacent normal tissue samples. Higher levels of *Fn* were found in tumor compared to adjacent normal (p=0.0033). Patients with *Fn* in tumor tissue were more likely to have proximal tumors (p=0.001), more advanced stage of cancer (p=0.033), higher level of invasion (p=0.014) and lower differentiation grade (p=0.011). |
| Kageyama, 2019 [109] | Japan | 2015-2017 | Esophagus, Stomach, Colorectal | Case-control | *Fn*: detection in saliva samples using 16S rRNA gene sequencing. Cancer: *unk.* | 59 patients with cancer in the gastrointestinal tract, 118 healthy controls. | *Fn* significantly more abundant in saliva of esophageal cancer patients as compared to controls (p<0.05). |
| Saito, 2019 [110] | Japan | *unk* | Colorectal | Case-control | *Fn*: detection in colonoscopy aspirates using 16S rRNA gene sequencing. Cancer: diagnosis based on colonoscopy. | 24 intramucosal CRC patients (stage 0 cancer), 47 colorectal adenoma patients, 10 healthy controls. | 60% of the *Fusobacterium* spp. were *Fn* in healthy controls. Overabundance of *Fusobacterium* spp. in intramucosal CRC patients *vs.* healthy controls. |
| Tunsjø, 2019 [82] | Norway | 2014-2017 | Colorectal | Case-control | *Fn*: detection in fecal and tissue samples using qPCR.  Cancer: diagnosis based on colonoscopy. | 25 CRC patients, 25 patients with adenomatous polyps, 22 healthy controls. | *Fn* significantly more abundant in CRC patients compared to the group with polyps (p<0.003) and the healthy controls (p<0.008). *Fn* detected in tumor tissue from 13 patients, of which 9 (69.2%) also had high *Fn* levels in feces. |
| Yachida, 2019 [111] | Japan | *unk* | Colorectal | Case-control | *Fn*: detection in fecal samples using whole-genome shotgun sequencing.  Cancer: diagnosis based on colonoscopy and histological findings. | 225 CRC patients, 140 adenoma patients, 251 healthy controls. | Significant increased abundance of *Fn* in pre-malignant cancer stages as well as the different CRC stages (p<0.005). |
| Zhang, 2019* [117] | USA | *unk* | Esophagus | Case-control | *Fn*: detection in tissue samples using WGS.  Cancer: *unk.* | 6 patients with esophageal adenocarcinoma, 8 patients with non-dysplastic Barrett’s esophagus and 9 healthy controls. | High abundance of *Fn* in samples from esophageal adenocarcinoma patients. |
| Alkharaan, 2020 [112] | Sweden | 2017-2019 | Pancreas | Case-control | *Fn*: DNA detection using qPCR and antibody detection using ELISA in plasma samples and saliva samples.  Cancer: dysplasia or cancer diagnosis based on histopathology. | 46 cancer patients, 45 low-grade dysplasia IPMN patients, 18 non-IPMN controls. | IgG binding reactivities in plasma significantly higher in cancer patients *vs.* non-IPMN control group (p<0.0006).  Salivary IgA reactivity to *Fn* higher in the cancer group compared to controls (based on 65 samples) (p<0.007). |
| Boehm, 2020 [113] | Lithuania, Germany | *unk* | Stomach, Colorectal | Case-control | *Fn*: detection in tissue samples using qPCR. Cancer: diagnosis based on histology. | 81 paired GC samples and 27 paired CRC samples (tumor and adjacent normal tissue), 17 gastritis patients, 9 atrophic gastritis or intestinal metaplasia patients. | *Fn* present in 16/27 (59.3%) CRC tumor tissue *vs.* 13/26 (50.0%) normal colon tissue samples. *Fn* load significantly correlated between CRC and GC tumor and normal tissues. *Fn* present in 23/80 (28.8%) GC tumor tissue *vs.* 18/78 (23.1%) normal gastric tissue samples. |
| Gantuya, 2020 [114] | Mongolia | 2014-2016 | Stomach | Cross-sectional | *Fn*: detection in tissue samples using 16S rRNA gene sequencing.  Cancer: diagnosis based on histopathology. | 48 GC patients, 120 non-cancer patients (20 healthy controls, 20 gastritis patients, 40 atrophy patients, 40 intestinal metaplasia patients). | *Fn* significantly enriched in GC tissue compared to tissue from intestinal metaplasia patients. |
| Kashani, 2020 [115] | Iran | 2017-2019 | Colorectal | Case-control | *Fn*: detection in tissue samples using PCR. Cancer: diagnosis based on colonoscopy and pathological findings. | 35 CRC patients, 45 controls with colorectal disorders other than cancer. | *Fn* present in 24/35 (68.6%) of the CRC patients, of which 11 had the *fad*A gene-positive *Fn*. *Fn* present in 11/45 (24.4%) of the controls, none of which carried the *fad*A gene. |
| Reynolds, 2020 [116] | Ireland | 2008-2017 | Rectum | Cross-sectional | *Fn*: detection in tumor and adjacent normal tissue samples using WGS.  Cancer: diagnosis based on biopsy, meeting the WHO mucinous rectal cancer diagnostic criteria. | 10 patients with mucinous rectal cancer. | *Fn* present in 10/10 tumor tissue samples and 9/10 adjacent normal tissue samples. |
| Eisele, 2021 [121] | Germany,  USA | 2010-2018 | Colorectal | Prospective cohort | *Fn*: detection in fecal samples using qPCR. Cancer: *unk.* | 105 CRC patients. | 22/105 (21.0%) of the CRC patients were classified as having high levels of *Fn* in feces (remaining 83 had none/low levels of *Fn*). OR for getting diagnosed with rectal cancer *vs.* colon cancer was 3.01 (95%CI 1.06-8.57; p=0.04) for those with high levels of *Fn.* In the same group, the OR for rectal cancer *vs.* proximal CC was 5.32 (95%CI 1.23-22.98; p=0.03). |
| Kawasaki, 2021 [118] | Japan | 2018-2020 | Esophagus | Case-control | *Fn*: detection in dental plaque samples, saliva and mucus samples from cancer tissue, using PCR. Cancer: *unk.* | 61 esophageal cancer patients, 62 healthy controls. | *Fn* detected in mucus of 35/37 (94.6%) esophageal cancer patients and in the saliva samples of all cancer patients and healthy controls. |
| Kurt, 2021 [119] | Turkey | 2018-2019 | Colorectal | Case-control | *Fn*: detection in tissue samples by qPCR, serum antibody detection using ELISA. Cancer: diagnosis based on colonoscopy. | 22 CRC patients, 35 precancerous-benign colon disease patients, 21 healthy controls. | *Fn* DNA present in tissue samples of 13/15 (86.7%) CRC patients *vs.* 19/26 (73.1%) P-BCD patients. Anti-*Fn*-IgA and -IgG were significantly higher in CRC patients *vs.* healthy controls. |
| Liang, 2021 [46] | China | 2009-2014 | Colorectal | Case-control | *Fn*: detection in fecal samples using qPCR.  Cancer: diagnosis based on colonoscopic examination and histopathological review | 210 CRC patients, 115 AA patients, 86 NAA patients, 265 healthy controls. | *Fn* significantly more abundant in CRC patients compared to controls (p<0.0001). *Fn* showed to be a potential marker for detection of CRC (sensitivity 81.8%; specificity 52.8%; positive predictive value 61.4%). |
| Pignatelli, 2021 [120] | Italy | 2018-2019 | Colon | Cross-sectional | *Fn*: detection in oral and (tumor and adjacent normal) tissue samples using qPCR. Cancer: *unk.* | 36 CC patients. | Abundance of *Fn* was significantly higher in oral samples (median 108.7 CFU/mL) compared to tumor tissue (median 4.8 CFU/mL) or adjacent normal tissue (2.19 CFU/mL). Difference in abundance between tumor tissue and adjacent normal tissue not significant. Abundance was not significantly different between cancer subsites (proximal, distal). |

*Only abstract available. *Fn: Fusobacterium nucleatum.* RFLP: restriction fragment length polymorphism. GC: gastric cancer. CRC: colorectal cancer. (q)PCR: (quantitative) polymerase chain reaction. SD: standard deviation. FISH: fluorescence in situ hybridization. NAA: non-advanced adenoma. AA: advanced adenoma. CC: colon cancer. ESCC: esophageal squamous cell carcinoma. EAC: esophageal adenocarcinoma. ICD-10: International Classification of Diseases (10^th^ revision). aHR: adjusted hazard ratio. OR: odds ratio. ELISA: enzyme-linked immunosorbent assay. WGS: whole-genome sequencing. WHO: World Health Organization. P-BCD: precancerous-benign colon disease. IPMN: intraductal papillary mucinous neoplasm.

**Table** **S11**. Characteristics and main outcomes of epidemiological studies assessing the association between ***Porphyromonas*** ***gingivalis*** and cancer in the gastrointestinal tract.

| **First author, year** | **Country** | **Study period** | **Malignancy/ malignancies** | **Study type** | **Diagnostic method(s)** | **Population size or no. cases & controls** | **Main outcomes** |
| --- | --- | --- | --- | --- | --- | --- | --- |
| Ahn, 2012 [128] | USA | 1988-2006 | GI cancers combined (esophagus, stomach, colon, rectum, anus, liver, pancreas) | Prospective cohort | *Pg*: detection of serum IgG titers using ELISA. Cancer: diagnosis according to ICD-10 C15, C16, C18-C22, C25 codes. | 817 orodigestive cancer deaths, 7,765 healthy controls. | Orodigestive cancer mortality in individuals with IgG titers against *Pg* ≥69.1 *vs.* <69.1: RR 2.25 (95%CI 1.23-4.14). |
| Michaud, 2013 [132] | Denmark, France Germany, Greece, Italy, The Netherlands, Norway, Spain, Sweden, United Kingdom | 1992-2000 | Pancreas | Prospective cohort | *Pg*: detection of antibody titers in serum samples using an immunoblot array. Cancer: data obtained from cancer registries, pathology based diagnosis. | 405 pancreatic cancer patients, 416 healthy controls. | Individuals with antibody titers >200 ng/ml had an over 2-fold higher risk of pancreatic cancer compared to individuals with an antibody titer of ≤200 ng/ml (OR 2.14; 95%CI 1.05-4.36). Mean follow-up time was 5 years. |
| Gao, 2016 [130] | China | 2010-2014 | Esophagus | Case-control | *Pg*: immuno-histochemial detection of *Pg* and gingipain Kgp (protease) and PCR for presence of rDNA in tumors *vs.* healthy tissues. Cancer: *unk.* | 100 ESCC tissue samples, 130 samples from healthy controls. | *Pg* present in: 61/100 cancer tissues, 12/100 adjacent normal tissues, 0/30 normal esophageal mucosa.  *Pg* positively correlated to poor differentiation, severe lymph node metastasis and cancer stage. |
| Sinha, 2016 [135] | USA | 1985-1987 | Colorectal | Case-control | *Pg*: detection in fecal samples using 16S rRNA gene sequencing. Cancer: newly diagnosed with colorectal adenocarcinoma. | 42 CRC patients, 89 healthy controls. | Significant higher abundance of *Pg* in CRC patients *vs.* controls (OR 3.83; 95%CI 1.03-14.22). |
| Peters, 2017 [133] | USA | 1993-2010 | Esophagus | Case–control study nested within a prospective cohort | *Pg*: detection in pre-diagnostic oral wash samples using 16S rRNA gene sequencing. Cancer: diagnosis according to ICD codes. EAC: 8140, 8144, 8480, 8481, 8560. ESCC: 8070, 8071, 8072, 8074, 8052. | 81 EAC patients, 160 controls. 25 ESCC patients, 50 controls. | *Pg* present in higher number of cancer patients *vs.* controls: EAC: OR 1.06 (95%CI 0.93–1.20). ESCC: OR 1.30 (95%CI 0.96-1.77). Follow-up time between oral sample and cancer diagnosis <1-9 years. |
| Gao, 2018 [131] | China | *unk* | Esophagus | Case-control | *Pg*: serum IgA and IgG titers measured by ELISA. Cancer: *unk.* | 96 ESCC patients, 130 healthy controls. | Median serum titers in ESCC patients *vs.* controls: IgG: 150.7 EU *vs.* 109.1 EU (p<0.001). IgA: 33.2 EU *vs.* 19.1 EU (p<0.01).  Diagnostic potential:  sensitivity: IgG 29.2%; IgA 52.1%. specificity: IgG 96.9%; IgA 70.8%. |
| Fan, 2018 [129] | USA | 2002-2008, 1993-2010 | Pancreas | Case–control study nested within a prospective cohort | *Pg*: detection in pre-diagnostic oral wash samples using 16S rRNA gene sequencing. Cancer: diagnosis according to ICD–O–2 codes C25.0–C25.3, C25.7–25.9. | 361 cancer patients, 371 healthy controls. | *Pg* present in higher number of cancer patients *vs.* controls, OR 1.60 (95%CI 1.15-2.22). Oral samples collected up to 10 years before cancer diagnosis. |
| Kageyama, 2019 [109] | Japan | 2015-2017 | Esophagus, Stomach, Colorectal | Case-control | *Pg*: detection in saliva samples using 16S rRNA gene sequencing. Cancer: *unk.* | 59 patients with cancer in the gastrointestinal tract, 118 healthy controls. | *Pg* significantly more abundant in saliva of EAC, GC and CRC patients as compared to controls (p<0.05). |
| Wei, 2020 [32] | China | 2017-2018 | Pancreas | Case-control | *Pg*: detection in saliva samples using 16S rRNA gene sequencing. Cancer: histopathologically-confirmed pancreatic adenocarcinoma. | 41 PDAC patients, 69 healthy controls. | Significant reduced abundance of *Pg* in saliva of PDAC patients *vs.* controls. |
| Kawasaki, 2021 [118] | Japan | 2018-2020 | Esophagus | Case-control | *Pg*: detection in dental plaque samples, saliva and mucus samples from cancer tissue, using PCR. Cancer: *unk.* | 61 patients with esophageal cancer, 62 healthy controls. | *Pg* detected in mucus of 13/37 (35.1%) esophageal cancer patients and in the saliva samples of 48/61 (78.7%) of the cancer patients and 49/62 (79.0%) of the healthy controls. |
| Pignatelli, 2021 [120] | Italy | 2018-2019 | Colon | Cross-sectional | *Pg*: DNA detection in oral and (tumor and adjacent normal) tissue samples using qPCR. Cancer: *unk.* | 36 patients with CC. | *Pg* present in oral samples but none of the colon samples (neither tumor tissue nor adjacent normal tissue). |
| Wang, 2021 [134] | China | 2012-2018 | Colorectal | Case-control | *Pg*: detection in fecal and tissue samples using 16S rDNA gene sequencing and IHC staining of tissue samples. Cancer: *unk.* | 23 CRC patients, 32 patients with CRC adenoma, 22 healthy controls. | High levels of *Pg* expression in 40.9% of CRC patients *vs.* 15.0% in adenoma group and 10.0% in controls. *Pg* significantly enriched in feces and CRC tissue compared to controls. |

GI: gastrointestinal. *Pg: Porphyromonas gingivalis.* ELISA: enzyme-linked immunosorbent assay. ICD: International Classification of Diseases. RR: relative risk. OR: odds ratio. PCR: polymerase chain reaction. ESCC: esophageal squamous cell carcinoma. CRC: colorectal cancer. EAC: esophageal adenocarcinoma. EU: ELISA unit. PDAC: pancreatic adenocarcinoma.

**Table S12**. Characteristics and main outcomes of epidemiological studies assessing the association between **nontyphoidal** ***Salmonella*** and cancer in the gastrointestinal tract.

| **First author, year** | **Country** | **Study period** | **Malignancy/ malignancies** | **Study type** | **Diagnostic method(s)** | **Population size or no. cases & controls** | **Main outcomes** |
| --- | --- | --- | --- | --- | --- | --- | --- |
| Kato, 2013 [140] | USA,  The Netherlands | 2003-2005 | Colorectal | Case-control | NT*S*: detection of *Salmonella* antiflagellin (FliC) IgG in blood samples using ELISA. Cancer: diagnosis based on histology. | 70 CRC patients, 23 controls with colorectal polyps, 74 healthy controls (from two independent cohorts). | Significantly higher Flic antibody titers in CRC patients and patients with colorectal polyps *vs.* healthy controls (Dutch cohort: 3.93 *vs.* 2.23; p=0.014; US cohort: 6.65 *vs.* 4.37; p<0.001). |
| Iyer, 2016 [139] | India | *unk* | Gallbladder | Cross-sectional | NT*S*: DNA detection in tissue using PCR and whole exosome sequencing. Cancer: diagnosis based on histopathology. | 17 tumor tissues from GBC patients, 9 matched adjacent normal tissue. | None of gallbladder samples tested NT*S*-positive. Traces of typhoidal *Salmonella* found in 12/26 (46.2%) gallbladder isolates. *S*. Typhimurium in 10/26 (38.5%) samples, *S*. Choleraesuis in 5/26 samples (19.2%). |
| Lu, 2017 [141] | USA | *unk* | Colorectal | Case-control | NT*S:* AvrA protein detection in fecal samples using rt-PCR, AvrA staining and IHC of human TMA  Cancer: diagnosis based on histopathology. | TMAs from 48 CRC tumors, 13 adjacent normal mucosa, 14 metastasized lymph nodes, 61 benign lesions, 19 healthy colorectal mucosa. 24 fecal samples from healthy controls. | Significantly lower mean normalized AvrA staining score in CRC tumors (1.37) *vs.* healthy mucosa (1.96) (p=0.013). Significantly higher staining score in adjacent normal mucosa (2.72) *vs.* healthy mucosa (1.96) (p=0.018). IHC-results: AvrA present in cancer tissue, absent in normal mucosa. Fecal samples exhibited amplification for AvrA. |
| Mughini-Gras, 2018 [142] | The Netherlands | 2000-2015 | Colon | Retrospective cohort | NT*S*: culture confirmed infection (feces, blood, other). Cancer: diagnosis according to ICD-10 C180-C187 codes. | 96 CC patients. Reference population: ~17 million. | The SIRs of developing CC after NT*S* infection expressed as compared to the general population were: - Overall CC: 1.17 (95%CI 0.95-1.43). - Proximal CC: 1.48 (95%CI 1.14-1.88; p<0.01) - Distal CC: 0.86 (95%CI 0.57-1.24). In patients aged <60 years when infected a higher risk was observed: SIR 1.54 (95%CI 1.09-2.10). |
| De Savornin-Lohman, 2020 [29] | The Netherlands | 2000-2016 | Biliary tract | Retrospective cohort | NT*S*: culture confirmed infection (feces, blood, other). Cancer: diagnosis according to ICD-O C239, C240, C242-C244, C248, C249 codes. | 9 patients with biliary tract cancer. Reference population: ~17 million. | Individuals with a history of NT*S* infection had a 1.5-fold increased risk of developing biliary tract cancer as compared to the general population (SIR 1.53; 95%CI 0.70-2.91). The risk was slightly higher for people infected before 60 years of age: SIR 1.74 (95%CI 0.36–5.04). |
| Chang, 2021 [137] | Taiwan | 2000-2013 | Stomach, Colorectal, Liver, Biliary tract, Pancreas | Retrospective cohort | NT*S*: culture confirmed infection (feces, blood) with hospitalization. Cancer: diagnosis according to ICD-9 151, 153-157 codes. | 9097 NT*S*-patients, 9097 non-NT*S* controls. 192 cancer patients (stomach n=37, colorectal n=55, biliary tract n=11, liver n=70, pancreas n=7). | The HRs of developing cancer after NT*S* infection as compared to non-NT*S* controls were: - Stomach 2.02 (95%CI 1.18-3.45); - Colorectal: 1.04 (95%CI 0.72-1.50); - Liver 1.03 (95%CI 0.72-1.47); - Biliary tract: 1.79 (95%CI 0.65-4.97); - Pancreas: 0.81 (95%CI 0.29-2.30). All combined 1.24 (95%CI 1.01-1.53). |
| Duijster, 2021 [138] | Denmark | 1994-2016 | Colon | Retrospective cohort | NT*S*: culture confirmed infection (feces, blood, other). Cancer: diagnosis according to ICD-10 C180-C187 codes. | 245 CC patients. Reference population: 7.6 million. | The HRs of developing CC after NTS infection as compared to non-NTS controls were:  - Overall CC 0.99 (95%CI 0.88-1.13). - - Proximal CC: 1.09 (95%CI 0.93-1.29) - Distal CC: 0.87 (95%CI 0.71-1.05) |

NT*S:* nontyphoidal *Salmonella.* ELISA: enzyme-linked immunosorbent assay. CRC: colorectal cancer. IHC: immunohistochemistry. TMA: tissue microarray. ICD: International Classification of Diseases. CC: colon cancer. SIR: standardized incidence ratio. HR: hazard ratio.

**Table S13**. Characteristics and main outcomes of epidemiological studies assessing the association between ***Salmonella*** **Typhi** and cancer in the gastrointestinal tract.

| **First author, year** | **Country** | **Study period** | **Malignancy/ malignancies** | **Study type** | **Diagnostic method(s)** | **Population size or no. cases & controls** | **Main outcomes** |
| --- | --- | --- | --- | --- | --- | --- | --- |
| Welton, 1979 [163] | USA | 1922-1975 | Liver, Biliary tract, Gallbladder | Case–control | *S*T: detection in fecal samples using culturing methods. Cancer: death due to hepatobiliary tract cancer. | 471 *S. Typhi* carriers, 942 controls. | Risk of death due to hepatobiliary cancer 6 times higher in chronic typhoid carriers compared to controls (p<0.001). |
| Mellemgaard, 1988 [150] | Denmark | 1943-1982 | Liver,  Biliary tract, Gallbladder | Retrospective cohort | *S*T: registered chronic typhoid carriers. Cancer: *unk.* | 219 typhoid carriers of which 3 with hepatobiliary tract cancer. | Significant increased risk of hepatobiliary tract cancer among chronic typhoid carriers: SIR 3.85 (90%CI 1.05-9.94) (3 observed cancers, 0.78 expected cancers). |
| Csendes, 1994* [147] | Chile | *unk* | Gallbladder | Cross-sectional | *S*T: detection in bile by culturing methods. Cancer: *unk.* | 58 GBC patients, 67 common bile duct stone patients, 165 CL patients, 46 acute cholecystitis patients, 36 healthy controls. | Pathogenic bacteria present in 47/58 (81.0%) of the GBC patients *vs.* 52/165 (31.5%) of the CL patients. |
| Caygill, 1994 [146] | United Kingdom | 1964-*unk* | Colorectal, Gallbladder, Pancreas | Retrospective cohort | *S*T: *unk.* Cancer: *unk.* | 83 chronic carriers of *S.* Typhi or Paratyphi, 386 individuals with history of acute *S*. Typhi (without chronic carriage). | SIR GBC 167.0 (95%CI 54.1-389). Also 8-fold and 3-fold increased risk of pancreatic cancer and CRC respectively. |
| Strom, 1995 [160] | Bolivia, Mexico | 1984-1988 | Gallbladder | Case-control | *S*T: detection of antibodies using ELISA. Cancer: diagnosis based on histology. | 84 GBC/biliary tract cancer patients, 264 CL or choledocholithiasis patients and 126 controls without biliary stones. (not all included in serological analysis). | Seropositivity in 7/15 (46.7%) cancer patients, 5/10 (50.0%) CL or choledocholithiasis patients and 4/8 (50.0%) controls. Significant elevated risk of self-reported history of physician-diagnosed typhoid fever (OR 12.7; 95%CI 1.5-598). |
| Singh, 1996 [159] | India | *unk* | Gallbladder | Case-control | *S*T: detection in bile samples using culturing methods. Cancer: *unk.* | 38 GBC patients, 67 CL patients. | *S*T present in bile of 4/38 (10.5%) GBC patients and 2/67 (3.0%) CL patients. No mixed infections with other pathogens in GBC patients (i.e. based on bile samples). |
| Nath, 1997* [151] | India | *unk* | Gallbladder | Case-control | *S*T: detection in bile samples using culturing methods. Cancer: *unk.* | 28 GBC patients, 56 CL patients and 17 healthy controls. | Significant higher prevalence of *S*T in GBC patients *vs.* CL patients and controls (p<0.05). |
| Roa, 1999* [154] | Chile | *unk* | Gallbladder | Cross-sectional | *S*T: detection in bile samples using culturing methods.  Cancer: *unk* | 24 GBC patients, 468 chronic cholecystitis\\s patients, 140 acute cholecystitis patients, 5 gallbladder dysplasia patients. | 13/29 (44.8%) of patients with gallbladder cancer or dysplasia had positive bacterial cultures in bile, not including *S*T. |
| Dutta, 2000 [148] | India | 1994-1995 | Gallbladder | Case-control | *S*T: detection of antibodies against *S*T Vi antigen using ELISA. Cancer: gallbladder mass on ultrasound or computed tomography. | 37 GBC patients with concurrent CL, 80 cholelithiasis patients with without cancer. | Antibody positivity in 6/37 (16.2%) of GBC patients *vs.* 2/80 (2.5%) of controls without cancer. 14-fold increased risk of GBC in chronic typhoid carriers with CL. |
| Shukla, 2000 [158] | India | *unk* | Gallbladder | Case-control | *S*T: detection of antibodies against *S*T Vi antigen (ViAb) in serum using indirect haemagglutination assay. Cancer: *unk.* | 51 GBC patients, 56 patients with cholelithiasis, 40 healthy controls. | Significantly higher ViAb positivity in GBC patients (29%) *vs.* healthy controls (5%) (OR 7.92; 95%CI 1.69-37.09, p<0.01). No significant difference between CL patients and controls (OR 3.47; 95%CI 0.44-11.93, p>0.05). |
| Serra, 2002 [156] | Chile | 1992-1995 | Gallbladder | Case-control | *S*T: self-reported history of *S. Typhi* infection. Cancer: diagnosis based on histology. | 114 GBC patients, 114 hospitalized controls with CL. | 9.7% of GBC patients reported a history of *S*T infection *vs.* 7.5% of the controls. |
| Pandey, 2003 [153] | India | *unk* | Gallbladder | Case-control | *S*T: self-reported history of *S. Typhi* infection. Cancer: *unk.* | 64 GBC patients, 101 controls with CL. | 14/64 (21.9%) of GBC patients reported a history of typhoid fever *vs.* 13/101 (12.9%) of the controls. |
| Hazrah, 2004 [149] | India | 1997-1999 | Gallbladder | Cross-sectional | *S*T: culturing bacteria from the core of the gallstones. Cancer: *unk.* | 14 GBC patients, 83 patients with cholecystitis or choledocholithiasis, 2 patients with empyema, 1 patient with periampullary carcinoma. All 100 patients had CL as well. | *Salmonella* spp. present in 1.5% of patients and controls combined. |
| Yagyu, 2004 [164] | Japan | 1988-1990 | Gallbladder | Prospective cohort | *S*T: self-reported history of *S*T infection. Cancer: GBC diagnosis using ICD-9 classification. | 113,394 individuals (healthy at start of study). | 2/116 (1.7%) individuals who died from GBC during a mean of 9.7 years of follow-up had a self-reported history of typhoid fever. |
| Vaishnavi, 2005 [162] | India | 2000-2002 | Gallbladder | Case-control | *S*T: detection of antibodies against *S*T Vi antigen in serum using ELISA. Cancer: *unk.* | 446 patients with gastrointestinal, biliary or other related diseases including 27 GBC patients, 705 healthy controls. | Significantly higher antibody positivity in GBC patients (2/27, 7.4%) *vs.* healthy controls (13/705, 1.8%) (p<0.05). |
| Sharma, 2007* [157] | India | *unk* | Gallbladder | Case-control | *S*T: detection of antibodies against ViAb in serum and detection of bacteria in bile. Cancer: *unk.* | 65 GBC patients, 125 CL patients, 200 healthy controls. | Significantly higher ViAb positivity in GBC patients (20/65, 30.8%) *vs.* controls (22/200, 11.0%) (OR: 3.60; p<0.05). |
| Capoor, 2008 [145] | India | 2005-2007 | Gallbladder | Case-control | *S*T: detection of *S*T in bile and feces samples using culturing methods. Cancer: diagnosis based on histopathological examination. | 6 GBC patients, 53 patients with acute cholecystitis with concurrent CL, 45 patients with acute cholecystitis with gastrointestinal ailments requiring biliary drainage. | *S*T isolated from bile of 1/6 (16.7%) GBC patients and 2/53 (3.8%) patients with acute cholecystitis with concurrent CL. |
| Nath, 2008 [152] | India | 2004-2007 | Gallbladder | Case-control | *S*T: ViAb detection in serum using indirect hemagglutination assay. *S*T flagellin gene detection in bile using PCR. Cancer: diagnosis based on ultrasonography, aspiration cytology and/or histopathology. | 52 GBC patients, 223 with benign gallbladder diseases, 508 healthy controls, 424 corpses (without visible gallbladder pathology). | Significantly higher ViAb-positivity in GBC patients (20/52, 38.4%) *vs.* controls (47/508, 9.2%) (OR 6.13; p<0.001). Significantly higher prevalence of St flagellin positivity in GBC patients (35/52, 67.3%) *vs.* benign disease group (95/223, 42.6%) (p<0.01). No significant difference in *S*T isolation from gallbladder tissue of GBC patients (2/52, 3.8%) *vs.* benign disease group (2/223, 0.9%). |
| Tewari, 2010 [161] | India | 2007-2009 | Gallbladder | Case-control | *S*T: detection in tissue/bile using PCR and culturing methods, and antibody detection in serum using the Widal test and IHA. Cancer: *unk.* | 54 GBC patients, 54 controls with CL. | 24/54 (44.4%) and 12/54 (22.2%) of GBC patients positivity on Widal and IHA test respectively *vs.* 13/54 (24.1%) and 5/54 (9.3%) of the controls. Positive *S*T PCR in 18/54 (33.3%) tissue samples and 2/54 (3.7%) bile samples of GBC patients *vs.* none of the controls. |
| Safaeian, 2011 [155] | China | 1997-2001 | Biliary tract, Gallbladder | Case-control | *S*T: detection of Vi antibody against *S*T. Cancer: diagnosis according to ICD-9 156 codes. | 627 GBC/biliary tract cancers, 774 CL patients, 263 bile-duct stone patients, 959 healthy controls. | Low prevalence of chronic typhoid carriers. No significantly elevated seropositivity in cancer patients as compared to controls. Seropositivity in 1/457 (0.22%) cancer patients, 4/977 (0.41%) patients with stones and 1/859 (0.12%) healthy controls. |
| Scanu, 2015 [136] | India | 2009-2013 | Gallbladder | Case-control | *S*T: detection of antibodies against *S*T Vi antigen in serum. Cancer: *unk* | 23 GBC patients, 60 controls with benign gallbladder disease. | Significant higher ViAb-positivity in GBC patients (12/23, 52.2%) *vs.* benign disease group (7/60, 11.7%) (p<0.001). |
| Iyer, 2016 [139] | India | *unk* | Gallbladder | Cross-sectional | *S*T: DNA detection in (tumor and normal) tissue using PCR and whole exosome sequencing. Cancer: histopathologically confirmed gallbladder cancer. | 17 tumor tissues, 9 matched adjacent normal tissue. | None of gallbladder samples *S*T -positive based on PCR. Traces of *S*T found in 11/26 gallbladder isolates. |
| Tsuchiya, 2018 [85] | Bolivia,  Chile | 2014-2016 | Gallbladder | Case-control | *S*T: detection of microbes in bile using sequencing of V3-V4 region. Cancer: *unk.* | 7 GBC patients, 30 controls with CL. | *S*T t not detected in bile of GBC patients. |

*Only abstract available. ST: *Salmonella* Typhi. SIR: standardized incidence ratio. *Unk:* unknown. GBC: gallbladder carcinoma. CL: cholelithiasis. CRC: colorectal cancer. ELISA: enzyme-linked immunosorbent assay. OR: odds ratio. ViAb: antibodies against *Salmonella* Typhi Vi antigen. ICD: International Classification of Diseases. PCR: polymerase chain reaction. IHA: indirect hemagglutination assay.

**Table S14**. Characteristics and main outcomes of epidemiological studies assessing the association between ***Schistosoma* spp.** and cancer in the gastrointestinal tract

| **First author, year** | **Country** | **Study period** | **Malignancy/ malignancies** | **Study type** | **Diagnostic method(s)** | **Population size or no. cases & controls** | **Main outcomes** |
| --- | --- | --- | --- | --- | --- | --- | --- |
| Zhao, 1981 [175] | China | 1970s | Colon | Cross-sectional | *Schistosoma*: *unk.* Cancer: *unk.* | *unk.* | 337 CC patients with concurrent schistosomiasis. |
| Xu, 1984 [174] | China | 1973-1979 | Colorectal | Case-control | *S. japonicum*: *unk.* Cancer: diagnosis based on histopathology, radiology, endoscopy, surgery. | 98 CC patients, 154 rectal cancer patients, 252 controls with non-GI cancer, 252 healthy controls. | Strong correlation between infection with *S. japonicum* and mortality from CRC. RRs of rectal cancer in patients with history of schistosomiasis 8.3 (95%CI 3.1-22.6) and 4.5 (95%CI 1.7-12.1) as compared to non-GI cancers and healthy controls respectively. RR for colon cancer not significantly increased (1.20; 95%CI 0.48-3.18). |
| Iida, 1999 [176] | Japan | 1985-1996 | Liver | Retrospective cohort | *S. japonicum*: history of anti-schistosomiasis treatment, positive skin test or ultrasonographic/computer tomographic confirmation. Cancer: *unk.* | 26 HCC patients, 484 chronic schistosomiasis patients (follow-up time of up to 10 years). | Development of HCC in 26/484 (5.4%) of schistosomiasis patients. Hepatitis C virus seropositivity in 39.5% of HCC patients with schistosomiasis. Out of 571 autopsies, 21/54 (39%) of patients with HCC had concurrent schistosomiasis, also 21/144 (14.6%) of patients with chronic schistosomiasis had HCC *vs.* 33/427 (7.7%) individuals without schistosomiasis (p=0.015). |
| Qiu, 2005 [173] | China | 1995-2002 | Colon, Liver | Case-control | *S. japonicum*: history of infection retrieved from medical records, surveillance data or by questionnaire. Cancer: *unk* | 142 CC patients, 285 controls matched to CC patients, 127 liver cancer patients, 127 controls matched to liver cancer patients. | Significant increased risk of CC (OR 3.3; 95%CI 1.8-6.1) and liver cancer (OR 3.7; 95%CI 1.0-1.3) after schistosomiasis. Fraction attributable to schistosomiasis of 24% for CC and 27% for liver cancer. |
| Madbouly, 2007 [177] | Egypt | 1999-2001 | Colorectal | Case-control | *S. mansoni*: diagnosed by histopathologic examination of the colonic mucosa. Cancer: *unk.* | 60 CC patients with schistosomal colitis (SCC), 40 CC patients without schistosomal colitis (NDCC). | More mucinous adenocarcinomas in SCC group compared to NDCC group (35% *vs.* 10%, p= 0.02) and more late stage tumors. Patients in SCC group significantly younger than NDCC controls (34.5 *vs.* 50.7 years; p=0.02) |
| Toda, 2015 [178] | Brazil | 2002-2015 | Liver | Case series | *S. mansoni*: epidemiological evidence, history of schistosomiasis treatment, fecal samples, imaging patterns and/or histological findings. Cancer: noninvasive diagnostic criteria of American Association for Study of Liver Diseases. | 7 patients with history of *S. mansoni* infection and HCC. | All 7 HCC patients tested negative on serological test for hepatitis C virus-antibodies, 4 were positive on hepatitis B virus core antibodies . |

*Unk:* unknown. CC: colon cancer. Non-GI: outside the gastrointestinal tract. CRC: colorectal cancer. RR: relative risk. HCC: hepatocellular carcinoma. OR: odds ratio. SCC: schistosomal colits. NDCC: without schistosomal colitis.

**Table S15**. Characteristics and main outcomes of epidemiological studies assessing the association between ***Streptococcus* spp.** and cancer in the gastrointestinal tract.

| **First author, year** | **Country** | **Study period** | **Malignancy/ malignancies** | **Study type** | **Diagnostic method(s)** | **Population size or no. cases & controls** | **Main outcomes** |
| --- | --- | --- | --- | --- | --- | --- | --- |
| Murray, 1978 [186] | USA | 1970-1976 | Colon | Cross-sectional | *S. bovis*: *unk.* Cancer: *unk.* | 36 patients with *S. bovis* bacteremia, of which 10 with *S. bovis* IE. | 2/36 (5.6%) bacteremia patients had a concurrent CC diagnosis and another 2/36 (5.6%) had potentially malignant villous adenomas. |
| Ruoff, 1989 [187] | USA | 1982-1987 | Colon | Cross-sectional | *S. bovis*: detection in blood samples using culturing methods. Cancer: *unk.* | 38 patients with *S. bovis* bacteremia. | 15/38 (39.5%) of *S. bovis* bacteremia patients were diagnosed with CC. |
| Zarkin, 1990 [188] | USA | 1979-1988 | Colon | Cross-sectional | *S. bovis*: detection in blood samples using culturing methods. Cancer: *unk.* | 92 patients with *S. bovis* bacteremia, of which 26 with *S. bovis* IE. | 43/92 (46.7%) bacteremia patients underwent colonic evaluation (e.g. colonoscopy, pathologic examination). Colonic lesions found in 11/24 (26.2%) *S. bovis* bacteremia patients and 11/19 (57.9%) *S. bovis* IE patients. CC diagnosis 6 *S. bovis* IE patients. |
| Gonzalez-Quintela, 2001 [184] | Spain | 1993-2000 | Colon | Cross-sectional | *S. bovis*: detection in blood samples using culturing methods.  Cancer: *unk.* | 20 patients with *S. bovis* bacteremia. | Colon evaluation data available for 13 *S. bovis* bacteremia patients, of which 3/13 (15.0%) were diagnosed with CC. |
| Jean, 2004* [185] | Taiwan | 1992-2001 | Colon | Cross-sectional | *S. bovis*: detection in blood samples using the API20 strep method. Cancer: diagnosis based on colonoscopy. | 62 patients with *S. bovis* bacteremia. | 19/62 (30.6%) of bacteremia patients underwent colonoscopy, of which 9/19 (47.3%) had colonic lesions (carcinoma or tubular adenoma). |
| Corredoira, 2005 [183] | Spain | 1987-2003 | Colon | Prospective cohort | *Streptococcus*: detection in blood samples using culturing methods. Cancer: *unk.* | 617 patients with *Streptococcus* bacteremia, of which 41 with *Sgg*. | More frequent detection of (pre)malignant CC in patients with a history of *Sgg* bacteremia (24/42, 57.1%) *vs. S. salivarius* bacteremia (0/52) (p=0.005). |
| Beck, 2008 [182] | Germany | *unk* | Colon | Retrospective cohort | *Streptococcus*: detection in blood samples using 16S rRNA gene sequencing. Cancer: *unk.* | 58 patients with *Streptococcus* bacteremia of which 46 with available patient record. | Less than 10% of *Streptococcus* bacteremia patients were diagnosed with CC. |
| Corredoira, 2008* [190] | Spain | 1988-2007 | Colon, Other GI cancers | Prospective cohort | *Streptococcus:* detection in blood samples using API20 strep method or GP card of the VITEK2 system. Cancer: *unk.* | 133 bacteremias (90 *Sgg*, 15 *S. gallolyticus* subsp. *pasteurianus*, 28 S. *infantarius*). | 51/105 (48.5%) and 3/28 (10.7%) of *S.* *gallolyticus* and *S. infantarious* infections respectively developed/had CC. 6/105 (5.7%) and 16/28 (57.1%) of *S.* *gallolyticus* and *S. infantarious* infections respectively had noncolonic cancer, of which 12 were GI cancers, mainly pancreatic and biliary tract. |
| Abdulamir, 2009 [11] | Malaysia | 2006-2007 | Colorectal | Case-control | *S. gallolyticus*: detection of IgG antibodies in blood samples using ELISA. Cancer: diagnosis based on colonoscopy. | 50 CRC patients, 14 colorectal adenoma patients, 30 apparently healthy volunteers and 30 controls without colon tumors. | IgG antibody titers significantly higher in CRC patients (0.158 ± 0.032) and adenoma patients (0.173 ± 0.024) *vs.* healthy volunteers (0.064 ± 0.011) and controls (0.046 ± 0.024) (p<0.05). No difference in antibody titers between CRC patients and adenoma patients. |
| Vaska, 2009 [192] | Australia | 1999-2006 | Colon | Retrospective cohort | *S. bovis*: detection in blood samples using culturing methods. Cancer: diagnosis based on colonoscopy and histopathology. | 20 patients with *S. bovis* bacteremia, of which 10 caused by *Sgg* and 10 by *S. bovis* biotype II. | 9/10 patients with a history of *Sgg* bacteremia underwent colonoscopy, of which 5/9 (55.6%) were diagnosed with CC and 4 had polyps or adenomas. 0/5 patients with a history *S. bovis* biotype II bacteremia who underwent colonoscopy developed CC. |
| Boleij, 2010 [189] | USA, The Netherlands | *unk* | Colorectal | Case-control | *S. bovis*: detection in blood samples using culturing methods, detection of antibodies against *S. bovis* antigen RpL7/L12 using ELISA. Cancer: *unk.* | Dutch population: 82 CRC patients, 10 patients with a systemic bacterial infection, 127 healthy controls. USA population: 64 CRC or polyp patients, 48 healthy controls. | Higher immune response against the RpL7/L12 antigen in stage I/II CRC patients and polyp patients compared to healthy controls (p=0.013) or late stage CRC patients (p=0.025). |
| Fernández-Ruiz, 2010 [191] | Spain | 1997-2008 | Colon | Cross-sectional | *S. bovis*: detection in blood samples using culturing methods. Cancer: diagnosis based on colonoscopy or pathologic examination of endoscopic/surgical samples. | 59 patients with *S. bovis* bacteremia. | 4/59 (6.8%) of the bacteremia patients was diagnosed with a GI malignancy before the bacteremia (1 of which with CC). 33/59 (55.9%) of the bacteremia patients underwent colonic evaluation, most of them ≤3 months after the bacteremia. 6/33 (18.2%) patients had CC. |
| Rahimkhani, 2010 [19] | Iran | *unk* | Colorectal | Case-control | *S. bovis*: detection in fecal samples using culturing methods. Cancer: *unk.* | 30 CRC patients, 30 healthy controls. | *S. bovis* most abundant bacterial species in fecal samples of 9 CRC patients and 6 controls. |
| Boleij, 2012 [193] | USA, The Netherlands | *unk* | Colorectal | Case-control | *S. bovis*: detection in blood samples using culturing methods, detection of IgG antibodies against 4 pilus proteins using ELISA. Cancer: *unk.* | Dutch population: 37 CRC patients, 12 patients with polyps, 15 patients with clinical bacterial infection (*E. coli, Klebsiella pneumonia* or *Sgg*), 27 healthy controls. USA population: 33 CRC patients, 11 patients with polyps and 47 healthy controls. | The immune response to the 4 pilus proteins (Gallo1569, -2039, -2178, -2179) was specific for *Sgg* infection (compared to the other bacterial infections), however also high interindividual variation observed. |
| Garza‐González, 2012 [194] | USA | *unk* | Colon | Case-control | *S. bovis*: detection of serological markers in blood samples using Western blot. Cancer: diagnosis based on colonoscopy. | 133 patients with colonic adenomatous polyps, 53 healthy controls (with normal colonoscopy). | 22 immunogenic proteins detected by the Western blot, of which two (of ~22 kDa and ~30 kDa) were most prominent. Presence of the 22 kDa protein associated with a higher risk of adenomatous polyps (OR 7.98; 95%CI 3.54-17.93, p<0.001). Higher OR when both proteins were present (OR 22.37; 95%CI 3.77-131.64, p<0.001). |
| Zammit, 2014 [195] | Malta | 2007-2012 | Colon, Liver, Biliary tract, Pancreas | Retrospective cohort | *S. bovis*: bacteremia confirmed by blood culture, catalase test or other test.  Cancer: *unk.* | 42 patients with a history of *S. bovis* bacteremia, 2 CRC patients, 6 colorectal adenoma patients, 11 patients with hepatobiliary–pancreatic pathologies. | 19% of patients with history of *S. bovis* bacteremia had colonic adenomas or colonic adenocarcinomas. Choledocholithiasis or cholelithiasis with cholangitis or cholecystitis observed in 4/42 patients. 2/42 patients had pancreatic cancer. |
| Corredoira, 2015 [61] | Spain | 1988-2014 | Colorectal | Prospective cohort | *S. bovis*: detection using conventional phenotypic test and/or molecular test on blood samples. Cancer: colonoscopy. | 109 patients with *S. bovis* IE. | Colorectal neoplasia found in 70/93 (75.3%) of patients who underwent colonoscopy during acute *S. bovis* IE (25 NAA, 37 AA, 8 CRC). 43 patients with previous *S. bovis* IE developed colorectal neoplasm (20 NAA, 18 AA, 5 CRC) during follow-up after a mean of 60.6 months (range 12-181 months). |
| Paritsky, 2015 [198] | Israel | 2012-2013 | Colorectal | Cross-sectional | *S.bovis*: detection in tissue using a VITEK2 system. Cancer: diagnosis based on colonoscopy. | 203 patients who underwent colonoscopy (for different reasons). | 49/203 (24.1%) of the individuals who underwent colonoscopy were tested positive on *S. bovis.* Of these 49 *S. bovis*-positive individuals, 17 (34.7%) had a malignant tumor, 22 (44.9%) had polyps, 4 (8.2%) had colitis and 6 (12.2%) showed no colonic abnormalities. No malignant tumors were found among the 154 individuals who tested *S. bovis*-negative. |
| Butt, 2016 [196] | Spain | 2008-2013 | Colorectal | Case-control | *Streptococcus*: multiplex serology against 4 *sgg* antigens (Gallo1569, 2039, 2178, 2179). Cancer: histologically confirmed colorectal cancer. | 576 CRC patients, 576 healthy controls. | (Significant) increased risk of CRC associated with: - positivity to Gallo2039: OR 1.58 (95%CI 1.09-2.28); - positivity to Gallo2178: OR 1.58 (95%CI 1.09-2.30); - positivity to Gallo2179: OR 1.45 (95%CI 1.00-2.11); - Double positivity for Gallo2178 and Gallo 2179: OR 3.54 (95%CI 1.49-8.44); - Two or more positivity of Gallo1569, Gallo2178, Gallo2179: OR 1.93 (95%CI 1.04-3.56). Stronger association in people aged <65 years. |
| Butt, 2017 [197] | Germany | 2005-2013, 2003-2007 | Colorectal | Case-control | *Streptococcus*: detection of 11 *sgg* antigens in serum using multiplex serology. Cancer: newly diagnosed colon or rectum cancer patients (*nfs*). | 50 newly diagnosed CRC patients (*BliTz study*), 30 NAA patients, 100 AA patients, 228 healthy controls. 318 additional CRC patients from *DACHSplus* study included. | (Significant) increased risk of CRC associated with: - positivity to Gallo2178: OR 4.13 (95%CI 2.11-8.08) (*DACHSplus)*; - Gallo2178: OR 3.19 (95%CI 1.11–9.21) (*BliTz*); - positivity to ≥2 of the 6 new SGG markers: OR 1.81 (95%CI 1.07–3.06) (*DACHSplus)*; - positivity to any of the 11 SGG antigens: OR 1.27 (95%CI 0.64-2.51) (*BliTz*); - positivity to ≥2 of the 6 new SGG markers: OR 1.50 (95%CI 0.61-3.72) (*BliTz study*). |
| Butt, 2018a [199] | Denmark, France, Greece, Germany, Italy, Netherlands, Norway, Spain, Sweden, United Kingdom | 1992-2003 | Colorectal | Case–control study nested within a prospective cohort | *Streptococcus*: detection of 11 *sgg* antigens in serum using multiplex serology. Cancer: ICD-10 C18-C20 diagnosis. | 485 CRC patients (samples drawn 0.4-8.5 years before CRC diagnosis), 485 healthy controls who did not develop cancer. | Significant increased risk of CRC associated with: - positivity to any of the 11 SGG antigens: OR 1.36 (95%CI 1.04-1.77); - positivity to ≥2 of the 6 new SGG markers: OR 2.17 (95%CI 1.44-3.27). |
| Butt, 2018b [200] | US | *unk* | Colorectal | Prospective cohort | *Streptococcus*: detection of 9 *sgg* antigens in serum using multiplex serology. Cancer: ICD-O-3 C180-C189, C199, C209 | Participants selected from 10 prospective cohorts. 4,063 CRC patients (samples drawn up to >10 years before CRC diagnosis), 4,063 healthy controls. | Increased risk of CRC with positivity to Gallo2178: OR 1.23 (95%CI 0.99-1.52), particularly when diagnosed <10 years after blood draw (OR 1.40; 95%CI 1.09-1.79). No association between Gallo2178-positivity and CRC risk in the subgroup diagnosed ≥10 years after blood draw. |
| Guven, 2018* [201] | Turkey | *unk* | Colorectal | Case-control | *Streptococcus*: detection in saliva samples using PCR. Cancer: *unk.* | 71 CRC patients, 77 healthy controls (aged >50 years). | *Sgg* positivity similar in CRC patients (31%) *vs.* controls (27%). Significantly higher amount of *Sgg* in saliva of CRC patients *vs.* controls (4.12 ±0.99 and 3.15 ±0.58 log^10^ copies/ml respectively, p< 0.001). No difference in prevalence or quantity between different tumor locations (proximal *vs.* distal) or tumor stage (stage 1-3 *vs.* stage 4). |
| Kale, 2018* [202] | India | 2013-2017 | Rectum, Liver, Pancreas | Retrospective cohort study | *Streptococcus*: detection in blood, ascitic fluid, bile or pleural fluid. Cancer: *unk.* | 68 patients with a *S. gallolyticus subsp pasteurianus* infection, of which 7 with a malignancy. | 5/68 patients with *S. gallolyticus subsp pasteurianus* had concomitant HCC, 1/68 rectal adenocarcinoma, 1 pancreatic carcinoma. |
| Kwong, 2018 [17] | China | 2006-2015 | Colorectal | Retrospective cohort | *S. bovis*: culture-confirmed bacteremia. Cancer: *unk.* | 203 *S. bovis* bacteremia patients, 1,0115 matched controls without history of bacteremia. | 8/203 (3.9%) of the bacteremia patients developed CRC compared to 10/1,015 (1.0%) of the controls, aHR: 5.73 (95%CI 2.18-15.1; p=0.0004). |
| Bundgaard, 2019 [13] | Denmark | 2002-2010 | Colorectal | Prospective cohort | *S. bovis*: DNA detection in (tumor and adjacent normal) tissue samples using qPCR. Cancer: *unk.* | 99 CRC patients, 96 adenoma patients, 104 patients with diverticular disease. | *S. bovis* not found in any of the samples of all study participants. |
| Justesen, 2020* [9] | Denmark | 2007-2018 | Colorectal | Retrospective cohort | *S. bovis*: culture confirmed bacteremia (in blood samples). Cancer: *unk.* | 117 patients with bacteremia caused by *S. bovis*. Reference population: ~2 million. | 6/117 (5.1%) of the individuals with a history of bacteremia caused by *S. bovis* were diagnosed with CRC, 5 of them within 1 year after bacteremia. |
| Sheikh, 2020 [203] | Iran | 2017-2018 | Colorectal | Case-control | *Streptococcus*: detection in fecal samples using culturing and PCR. Cancer: clinical, radiological, histological criteria and endoscopy and colonoscopy. | 22 CRC patients, 44 IBD patients, 40 healthy controls. | Significantly higher *Sgg* positivity in CRC and IBD patients *vs.* controls based on culturing (p=0.013) and PCR (p=0.001). Culturing: *Sgg* positivity in 2/22 (9.1%) CRC patients and 7/44 (15.9%) IBD patients *vs.* 0/40 of the controls. PCR: *Sgg* positivity in 9/22 (40.9%) CRC patients, 15/44 (34.1%) IBD patients *vs.* 3/40 (7.5%) healthy controls. |
| Wang, 2020 [31] | China |  | Colorectal | Case-control | *Streptococcus* spp.: detection in (tumor) and mucosa using 16S rRNA gene sequencing. Cancer: *unk.* | Tumor, adjacent normal and off-tumor site tissue from 75 CRC patients and mucosa from 26 healthy controls. | Relative abundance of *Streptococcus* spp. significantly higher in tumor tissue *vs.* mucosa from healthy controls. |

*Only abstract available. *Unk*: unknown. IE: infective endocarditis. CC: colon cancer. *Sgg*: *Streptococcus gallolyticus* subsp. *gallolyticus.* GI: gastrointestinal. NAA: non-advanced adenoma. AA: advanced adenoma. CRC: colorectal cancer. OR: odds ratio. *Nfs:* not further specified. ICD(-O): International Classification of Diseases (for Oncology). PCR: polymerase chain reaction. HCC: hepatocellular carcinoma. aHR: adjusted hazard ratio. IBD: inflammatory bowel disease.

**Table S16**. Characteristics and main outcomes of epidemiological studies assessing the association between *Strongyloides stercoralis* and cancer in the gastrointestinal tract.

| **First author, year** | **Country** | **Study period** | **Malignancy/ malignancies** | **Study type** | **Diagnostic method(s)** | **Population size or no. cases & controls** | **Main outcomes** |
| --- | --- | --- | --- | --- | --- | --- | --- |
| Hirata, 2007 [211] | Japan | 1991-2005 | Liver, Biliary tract, Pancreas | Case-control | *Strongyloides*: detection in fecal samples using culturing methods.  Cancer: diagnosis based on histology, cytology, radiological findings. | 196 (liver, pancreatic, biliary tract) cancer patients, 1,458 controls without cancer. | Prevalence of *S. stercoralis* significantly higher in biliary tract cancer patients (18.4%) *vs.* controls (7.5%). OR for developing cancer in pancreas 2.2 (95%CI 0.7-6.6), liver 0.9 (95%CI 0.5-1.8), biliary tract 2.7 (95%CI 1.1-6.3). |
| Tanaka, 2016 [210] | Japan | 1991-2014 | Esophagus, Stomach, Colorectal, Liver, Biliary tract, Pancreas, | Retrospective cohort | *Strongyloides*: detection in fecal samples using culturing methods.  Cancer: diagnosis based on histology, cytology, radiological findings. | 1,352 cancer patients (different malignancies including 7 esophagus, 24 stomach, 15 colorectal, 2 pancreas, 9 liver, 10 biliary tract), 2,596 controls without cancer. | OR for developing cancer in esophagus 0.65 (95%CI 0.29-1.45), stomach 1.22 (95%CI 0.76-1.97), colorectal 0.94 (95%CI 0.53-1.66), pancreas 0.83 (95%CI 0.19-3.55), liver 0.72 (95%CI 0.35-1.47), biliary tract 1.90 (95%CI 0.93-3.87). |

OR: odds ratio.

**References**

7. Haghi, F., Goli, E., Mirzaei, B., and Zeighami, H. (2019). The association between fecal enterotoxigenic *B. fragilis* with colorectal cancer. *BMC Cancer*, 19(1), 1-4. doi: 10.1186/s12885-019-6115-1

8. Zamani, S., Taslimi, R., Sarabi, A., Jasemi, S., Sechi, L. A., Feizabadi, M. M. (2020). Enterotoxigenic *Bacteroides* *fragilis*: a possible etiological candidate for bacterially-induced colorectal precancerous and cancerous lesions. *Front. Cell. Inf. Microbiol.* 9, 449. doi: 10.3389/fcimb.2019.00449

9. Justesen, U. (2020). Bacteraemia with anaerobic bacteria and association with colorectal cancer, in ECCMID-2020. [conference abstract].

10. Ma, Y., Zhang, Y., Xiang, J., Xiang, S., Zhao, Y., Xiao, M., et al. (2021). Metagenome analysis of intestinal bacteria in healthy people, patients with inflammatory bowel disease and colorectal cancer. *Front. Cell. Inf. Microbiol*., 11. doi: 10.3389/fcimb.2021.599734

11. Abdulamir, A.S., Hafidh, R. R., Mahdi, L. K., Al-jeboori, T., and Abubaker, F. (2009). Investigation into the controversial association of *Streptococcus* *gallolyticus* with colorectal cancer and adenoma. *BMC Cancer*, 9(1), 1-12. doi: 10.1186/1471-2407-9-403

12. Boleij, A., Hechenbleikner, E. M., Goodwin, A. C., Badani, R., Stein, E. M., Lazarev, M. G., et al. (2015). The *Bacteroides fragilis* toxin gene is prevalent in the colon mucosa of colorectal cancer patients. *Clin. Infect. Dis.,* 60(2), 208-215. doi: 10.1093/cid/ciu787

.

13. Bundgaard-Nielsen, C., Baandrup, U. T., Nielsen, L. P., and Sørensen, S. (2019). The presence of bacteria varies between colorectal adenocarcinomas, precursor lesions and non-malignant tissue. *BMC Cancer*, 19(1), 1-13. doi: 10.1186/s12885-019-5571-y

14. Fukugaiti, M. H., Ignacio, A., Fernandes, M. R., Ribeiro Júnior, U., Nakano, V., and Avila-Campos, M. J. (2015). High occurrence of *Fusobacterium* *nucleatum* and *Clostridium* *difficile* in the intestinal microbiota of colorectal carcinoma patients. *Braz. J. Microbiol*., 46(4), 1135-1140. doi: 10.1590/S1517-838246420140665

15. Hale, V. L., Jeraldo, P., Chen, J., Mundy, M., Yao, J., Priya, S., et al. (2018). Distinct microbes, metabolites, and ecologies define the microbiome in deficient and proficient mismatch repair colorectal cancers. *Genome Med*., 10(1), 1-13. doi: 10.1186/s13073-018-0586-6

16. Keenan, J., Aitchison, A., Greenlees, R., and Frizelle, F. (2014). Are toxin-producing strains of *Bacteroides* *fragilis* involved in colorectal cancer?:CR27. *Anz. J. Surg*. 84.

17. Kwong, T. N., Wang, X., Nakatsu, G., Chow, T. C., Tipoe, T., Dai, R. Z., et al. (2018). Association between bacteremia from specific microbes and subsequent diagnosis of colorectal cancer. *Gastroenterology*, 155(2), 383-390. doi: 10.1053/j.gastro.2018.04.028

18. Purcell, R. V., Pearson, J., Aitchison, A., Dixon, L., Frizelle, F. A., and Keenan, J. I. (2017). Colonization with enterotoxigenic *Bacteroides* *fragilis* is associated with early-stage colorectal neoplasia. *PloS One*, 12(2), e0171602. doi: 10.1371/journal.pone.0171602

19. Rahimkhani, M., Mohagheghi, M. A., and Yaraei, K. (2010). Fecal microbial flora in colorectal cancer. *Afr. J. Microbiol. Res*., 4(23), 2622-2625. doi: 10.5897/AJMR.9000323

20. Ulger Toprak, N., Yagci, A. Y. Ş. E. G. Ü. L., Gulluoglu, B. M., Akin, M. L., Demirkalem, P., Celenk, T., et al. (2006). A possible role of *Bacteroides* *fragilis* enterotoxin in the aetiology of colorectal cancer. *Clin. Microbiol. Infect*., 12(8), 782-786. doi: 10.1111/j.1469-0691.2006.01494.x

21. Viljoen, K. S., Dakshinamurthy, A., Goldberg, P., and Blackburn, J. M. (2015). Quantitative profiling of colorectal cancer-associated bacteria reveals associations between fusobacterium spp., enterotoxigenic *Bacteroides* *fragilis* (ETBF) and clinicopathological features of colorectal cancer. *PloS One*, 10(3), e0119462. doi: 10.1371/journal.pone.0119462

22. Xie, L. L., Wu, N., Zhu, Y. M., Qiu, X. Y., Chen, G. D., Zhang, L. M., et al. (2016). Expression of enterotoxigenic *Bacteroides* *fragilis* and polyketide synthase gene-expressing *Escherichia* *coli* in colorectal adenoma patients. *Zhonghua yi xue za zhi*, 96(12), 954-959. doi: 10.3760/cma.j.issn.0376-2491.2016.12.010

25. Alexander, J. L., Scott, A., Mroz, A., Perdones-Montero, A., Mckenzie, J., Rees, D. N., et al. (2016). 91 Mass spectrometry imaging (MSI) of microbiome-metabolome interactions in colorectal cancer. *Gastroenterology*, 150(4), S23. doi: 10.1016/S0016-5085(16)30202-5

26. Allali, I., Delgado, S., Marron, P. I., Astudillo, A., Yeh, J. J., Ghazal, H., et al. (2015). Gut microbiome compositional and functional differences between tumor and non-tumor adjacent tissues from cohorts from the US and Spain. *Gut Microbes*, 6(3), 161-172. doi: 10.1080/19490976.2015.1039223

27. Blackett, K. L., Siddhi, S. S., Cleary, S., Steed, H., Miller, M. H., Macfarlane, S., et al. (2013). Oesophageal bacterial biofilm changes in gastro‐oesophageal reflux disease, Barrett's and oesophageal carcinoma: association or causality?. *Aliment. Pharmacol. Ther*., 37(11), 1084-1092. doi: 10.1111/apt.12317

28. Brauner, A., Brandt, L., Frisan, T., Thelestam, M., and Ekbom, A. (2010). Is there a risk of cancer development after *Campylobacter* infection?. *Scand. J. Gastroenterol*., 45(7-8), 893-897. doi: 10.3109/00365521003734133

29. De Savornin Lohman, E., Duijster, J., Groot Koerkamp, B., van der Post, R., Franz, E., Mughini Gras, L., et al. (2020). Severe *Salmonella* spp. Or *Campylobacter* spp. infection and the risk of biliary tract cancer: A population-based study. *Cancers*, 12(11), 3348. doi: 10.3390/cancers12113348

30. Mughini Gras, L. (2017). Colon cancer risk in patients with a history of *Campylobacter* infection, in 19th International workshop CHRO 2017 on *Campylobacter*, *Helicobacter* and Related Organisms. 2017: Nantes, France. p. 250. [conference abstract].Alexander, J.L., et al., 91 Mass spectrometry imaging (MSI) of microbiome-metabolome interactions in colorectal cancer. Gastroenterology, 2016. 150(4): p. S23.

31. Wang, Y., Zhang, C., Hou, S., Wu, X., Liu, J., and Wan, X. (2020). Analyses of potential driver and passenger bacteria in human colorectal cancer. *Cancer Manag. Res*., 12, 11553. doi: 10.2147/CMAR.S275316

32. Wei, A. L., Li, M., Li, G. Q., Wang, X., Hu, W. M., Li, Z. L., et al. (2020). Oral microbiome and pancreatic cancer. *World J. Gastroenterol*., 26(48), 7679. doi: 10.3748/wjg.v26.i48.7679

33. Wu, N., Yang, X., Zhang, R., Li, J., Xiao, X., Hu, Y., et al. (2013). Dysbiosis signature of fecal microbiota in colorectal cancer patients. *Microb. Ecol*., 66(2), 462-470. doi: 10.1007/s00248-013-0245-9

40. Ahn, J., Sinha, R., Pei, Z., Dominianni, C., Wu, J., Shi, J., ... & Yang, L. (2013). Human gut microbiome and risk for colorectal cancer*. J. Nat. Cancer Inst*., 105(24), 1907-1911. doi: 10.1093/jnci/djt300

41. Liang, Q., Chiu, J., Chen, Y., Huang, Y., Higashimori, A., Fang, J., et al. Fecal bacteria act as novel biomarkers for noninvasive diagnosis of colorectal cancer. *Clin. Cancer Res*., 23(8), 2061-2070. doi: 10.1158/1078-0432.CCR-16-1599

42. Lu, Y., Zhang, B. Y., Shi, J. S., and Wu, L. Q. (2004). Expression of the bacterial gene in gallbladder carcinoma tissue and bile. *Hepatobiliary & Pancreatic Diseases International: HBPD INT*, 3(1), 133-135.

43. Ohigashi, S., Sudo, K., Kobayashi, D., Takahashi, O., Takahashi, T., Asahara, T., et al. (2013). Occurence of initiation and progression of colorectal cancer in the changed intestinal environment. *J. Clin. Oncol.,* 31(4), 416. doi: 10.1200/jco.2013.31.4_suppl.416

44. Hsieh, Y. Y., Tung, S. Y., Pan, H. Y., Yen, C. W., Xu, H. W., Lin, Y. J., et al. (2018). Increased abundance of *Clostridium* and *Fusobacterium* in gastric microbiota of patients with gastric cancer in Taiwan. *Scientific Rep*., 8(1), 1-11. doi: 10.1038/s41598-017-18596-0

45. Jahani-Sherafat, S., Azimirad, M., Alebouyeh, M., Amoli, H. A., Hosseini, P., Ghasemian-Safaei, H., et al. (2019). The rate and importance of *Clostridium* *difficile* in colorectal cancer patients. *Gastroenterol. Hepatol. Bed Bench*, 12(4), 358-363.

46. Liang, J. Q., Wong, S. H., Szeto, C. H., Chu, E. S., Lau, H. C., Chen, Y., et al. (2021). Fecal microbial DNA markers serve for screening colorectal neoplasm in asymptomatic subjects. *J. Gastroenterol. Hepatol*., 36(4), 1035-1043. doi: 10.1111/jgh.15171

48. Kalantari, N., Gorgani-Firouzjaee, T., Ghaffari, S., Bayani, M., Ghaffari, T., and Chehrazi, M. (2020). Association between *Cryptosporidium* infection and cancer: a systematic review and meta-analysis. *Parasitol. Int*., 74, 101979. doi: 10.1016/j.parint.2019.101979

51. Sulżyc-Bielicka, V., Kołodziejczyk, L., Jaczewska, S., Bielicki, D., Safranow, K., Bielicki, P., et al. (2018). Colorectal cancer and *Cryptosporidium* spp. infection. *PLoS One*, 13(4), e0195834. doi: 10.1371/journal.pone.0195834

52. Sulżyc-Bielicka, V., Kuźna-Grygiel, W., Kołodziejczyk, L., Bielicki, D., Kładny, J., Stępień-Korzonek, M., et al. (2007). Cryptosporidiosis in patients with colorectal cancer. *J. Parasitol*., 93(3), 722-724. doi: 10.1645/GE-1025R1.1

53. Essid, R., Menotti, J., Hanen, C., Aoun, K., and Bouratbine, A. (2018). Genetic diversity of *Cryptosporidium* isolates from human populations in an urban area of Northern Tunisia. *Infect. Genet. Evol*., 58, 237-242. doi: 10.1016/j.meegid.2018.01.004

54. Osman, M., Benamrouz, S., Guyot, K., Baydoun, M., Frealle, E., Chabe, M., et al. (2017). High association of *Cryptosporidium* spp. infection with colon adenocarcinoma in Lebanese patients. *PloS One*, 12(12), e0189422. doi: 10.1371/journal.pone.0189422

55. Sanad, M. M., Thagfan, F. A., Al Olayan, E. M., Almogren, A., Al Hammaad, A., Al-Mawash, A., et al. (2014). Opportunistic coccidian parasites among Saudi cancer patients presenting with diarrhea: prevalence and immune status. *Res. J. Parasitol*., 9(2), 55-63. doi: 10.3923/jp.2014.55.63

56. Sulżyc-Bielicka, V., Kołodziejczyk, L., Jaczewska, S., Bielicki, D., Kładny, J., and Safranow, K. (2012). Prevalence of *Cryptosporidium* sp. in patients with colorectal cancer. *Pol. J. Surg*., 2012. 84(7): p. 348-351. doi: 10.2478/v10035-012-0058-4

58. De Almeida, C. V., Lulli, M., di Pilato, V., Schiavone, N., Russo, E., Nannini, G., et al. (2019). Differential responses of colorectal cancer cell lines to *Enterococcus* *faecalis’* strains isolated from healthy donors and colorectal cancer patients. *J. Clin. Med*., 8(3), 388. doi: 10.3390/jcm8030388

60. Balamurugan, R., Rajendiran, E., George, S., Samuel, G. V., and Ramakrishna, B. S. (2008). Real‐time polymerase chain reaction quantification of specific butyrate‐producing bacteria, *Desulfovibrio* and *Enterococcus* *faecalis* in the feces of patients with colorectal cancer. *J. Gastroenterol. Hepatol*., 23(8pt1), 1298-1303. doi: 10.1111/j.1440-1746.2008.05490.x

61. Corredoira, J., García-País, M. J., Coira, A., Rabuñal, R., García-Garrote, F., Pita, J., et al. (2015). Differences between endocarditis caused by *Streptococcus* *bovis* and *Enterococcus* spp. and their association with colorectal cancer. *Eur. J. Clin. Microbiol. Infect. Dis*., 34(8), 1657-1665. doi: 10.1007/s10096-015-2402-1

62. Geravand, M., Fallah, P., Yaghoobi, M. H., Soleimanifar, F., Farid, M., Zinatizadeh, N., et al. (2019). Investigation of Enterococcus faecalis population in patients with polyp and colorectal cancer in comparison of healthy individuals. *Arq. Gastroenterol*., 56, 141-145. doi: 10.1590/S0004-2803.201900000-28

63. D’asheesh, T. I. A., Hussen, B. M., Al-Marzoqi, A. H., and Ghasemian, A. (2021). Assessment of oncogenic role of intestinal microbiota in colorectal cancer patients. *J. Gastrointest. Cancer*, 52(3), 1016-1021. doi: 10.1007/s12029-020-00531-8

64. Pericas, J. M., Corredoira, J., Moreno, A., García-País, M. J., Falces, C., Rabuñal, R., et al. (2017). Relationship between *Enterococcus* *faecalis* infective endocarditis and colorectal neoplasm: preliminary results from a cohort of 154 patients. *Rev. Esp. Cardiol*. (English Edition), 70(6), 451-458. doi: 10.1016/j.rec.2016.10.013

65. Rezasoltani, S., Aghdaei, H. A., Dabiri, H., Sepahi, A. A., Modarressi, M. H., and Mojarad, E. N. (2018). The association between fecal microbiota and different types of colorectal polyp as precursors of colorectal cancer. *Microb. Pathog.*, 124, 244-249. doi: 10.1016/j.micpath.2018.08.035

66. Shoji, M., Sasaki, Y., Abe, Y., Nishise, S., Yaoita, T., Yagi, M., et al. (2021). Characteristics of the gut microbiome profile in obese patients with colorectal cancer. *JGH Open*, 5(4), 498-507. doi: 10.1002/jgh3.12529

67. Wang, T., Cai, G., Qiu, Y., Fei, N., Zhang, M., Pang, X., et al. (2012). Structural segregation of gut microbiota between colorectal cancer patients and healthy volunteers. *ISME J*., 6(2), 320-329. doi: 10.1038/ismej.2011.109

68. Zhou, Y., He, H., Xu, H., Li, Y., Li, Z., Du, Y., et al. (2016). Association of oncogenic bacteria with colorectal cancer in South China. *Oncotarget*, 7(49), 80794. doi: 10.18632/oncotarget.13094

73. Tang, L., Zhou, Y. J., Zhu, S., Liang, G. D., Zhuang, H., Zhao, M. F., et al. (2020). *E. coli* diversity: low in colorectal cancer. *BMC Med. Genom*., 13(1), 1-17. doi: 10.1186/s12920-020-0704-3

75. Bonnet, M., Buc, E., Sauvanet, P., Darcha, C., Dubois, D., Pereira, B., et al. (2014). Colonization of the human gut by *E. coli* and colorectal cancer risk. *Clin. Cancer Res*., 20(4), 859-867. doi: 10.1158/1078-0432.CCR-13-1343

76. Buc, E., Dubois, D., Sauvanet, P., Raisch, J., Delmas, J., Darfeuille-Michaud, A., et al. (2013). High prevalence of mucosa-associated *E. coli* producing cyclomodulin and genotoxin in colon cancer*. PloS One*, 8(2), e56964. doi: 10.1371/journal.pone.0056964

77. Iyadorai, T., Mariappan, V., Vellasamy, K. M., Wanyiri, J. W., Roslani, A. C., Lee, G. K., et al. (2020). Prevalence and association of pks+ *Escherichia coli* with colorectal cancer in patients at the University Malaya Medical Centre, Malaysia. *PloS One*, 15(1), e0228217. doi: 10.1371/journal.pone.0228217

78. Kohoutova, D., Smajs, D., Moravkova, P., Cyrany, J., Moravkova, M., Forstlova, M., et al. (2014). *Escherichia* *coli* strains of phylogenetic group B2 and D and bacteriocin production are associated with advanced colorectal neoplasia. *BMC Infect. Dis*., 14(1), 1-8. doi: 10.1186/s12879-014-0733-7

79. Pleguezuelos-Manzano, C., Puschhof, J., Huber, A. R., van Hoeck, A., Wood, H. M., Nomburg, J., et al. (2020). Mutational signature in colorectal cancer caused by genotoxic pks+ *E. coli*. *Nature*, 580(7802), 269-273. doi: 10.1038/s41586-020-2080-8

80. Rezasoltani, S., Asadzadeh, A. H., Dabiri, H., Akhavan, S. A., Modarressi, M. H., and Nazemalhosseini, M. E. (2020). Investigating the number of *Lactobaccilus*, *Escherichia* *coli* and *Prevotella* in fecal microbiota of adenomatous polyposis and colorectal cancer patients. *Tehran Univ. Med. J*., 78(3), 137-143.

81. Shimpoh, T., Hirata, Y., Ihara, S., Suzuki, N., Kinoshita, H., Hayakawa, Y., et al. (2017). Prevalence of pks-positive *Escherichia* *coli* in Japanese patients with or without colorectal cancer. *Gut Pathog.*, 9(1), 1-8. doi: 10.1186/s13099-017-0185-x

82. Tunsjø, H. S., Gundersen, G., Rangnes, F., Noone, J. C., Endres, A., and Bemanian, V. (2019). Detection of Fusobacterium nucleatum in stool and colonic tissues from Norwegian colorectal cancer patients. *Eur. J. Clin. Microbiol. Infect. Dis*., 38(7), 1367-1376. doi: 10.1007/s10096-019-03562-7

83. Yoshikawa, Y., Tsunematsu, Y., Matsuzaki, N., Hirayama, Y., Higashiguchi, F., Sato, M., et al. (2020). Characterization of colibactin-producing *Escherichia* *coli* isolated from Japanese patients with colorectal cancer. *Jpn. J. Infect. Dis*., 73(6), 437-442. doi: 10.7883/yoken.JJID.2020.066

84. Zarei, O., Arabestan, M. R., Majlesi, A., Mohammadi, Y., and Alikhani, M. Y. (2019). Determination of virulence determinants of *Escherichia* *coli* strains isolated from patients with colorectal cancer compared to the healthy subjects. *Gastroenterol. Hepatol. Bed Bench*, 12(1), 52.

85. Tsuchiya, Y., Loza, E., Villa-Gomez, G., Trujillo, C. C., Baez, S., Asai, T., et al. (2018). Metagenomics of microbial communities in gallbladder bile from patients with gallbladder cancer or cholelithiasis. *Asian Pac. J. Cancer Prev*., 19(4), 961. doi: 10.22034/APJCP.2018.19.4.961

89. Castellarin, M., Warren, R. L., Freeman, J. D., Dreolini, L., Krzywinski, M., Strauss, J., et al. (2012). *Fusobacterium* *nucleatum* infection is prevalent in human colorectal carcinoma. *Genome Res*., 22(2), 299-306. doi: 10.1101/gr.126516.111

90. Flanagan, L., Schmid, J., Ebert, M., Soucek, P., Kunicka, T., Liska, V., et al. (2014). *Fusobacterium* *nucleatum* associates with stages of colorectal neoplasia development, colorectal cancer and disease outcome. *Eur. J. Clin. Microbiol. Infect. Dis*., 33(8), 1381-1390. doi: 10.1007/s10096-014-2081-3

91. Ito, M., Kanno, S., Nosho, K., Sukawa, Y., Mitsuhashi, K., Kurihara, H., et al. (2015). Association of *Fusobacterium* *nucleatum* with clinical and molecular features in colorectal serrated pathway. *Int. J. Cancer*, 137(6), 1258-1268. doi: 10.1002/ijc.29488

92. Mira-Pascual, L., Cabrera-Rubio, R., Ocon, S., Costales, P., Parra, A., Suarez, A., et al. (2015). Microbial mucosal colonic shifts associated with the development of colorectal cancer reveal the presence of different bacterial and archaeal biomarkers. *J. Gastroenterol*., 50(2), 167-179. doi: 10.1007/s00535-014-0963-x

93. Amitay, E. L., Werner, S., Vital, M., Pieper, D. H., Höfler, D., Gierse, I. J., et al. (2017). *Fusobacterium* and colorectal cancer: causal factor or passenger? Results from a large colorectal cancer screening study. *Carcinogenesis*, 38(8), 781-788. doi: 10.1093/carcin/bgx053

94. Drewes, J. L., White, J. R., Dejea, C. M., Fathi, P., Iyadorai, T., Vadivelu, J., et al. (2017). High-resolution bacterial 16S rRNA gene profile meta-analysis and biofilm status reveal common colorectal cancer consortia. *NPJ Biofilms Microbiomes*, 3(1), 1-12. doi: 10.1038/s41522-017-0040-3

95. Eklöf, V., Löfgren‐Burström, A., Zingmark, C., Edin, S., Larsson, P., Karling, P., et al. (2017). Cancer‐associated fecal microbial markers in colorectal cancer detection. *Int. J. Cancer*, 141(12), 2528-2536. doi: 10.1002/ijc.31011

96. Wang, H. F., Li, L. F., Guo, S. H., Zeng, Q. Y., Ning, F., Liu, W. L., et al. (2016). Evaluation of antibody level against *Fusobacterium* *nucleatum* in the serological diagnosis of colorectal cancer. *Scientific Rep*., 6(1), 1-10. doi: 10.1038/srep33440

97. Xie, Y.H. and Fang, J.Y. (2016). Single fecal abundance of *Fusobacterium* *nucleatum* for early- and late-stage detection of colorectal cancer. *J. Dig. Dis*., 2016. 17: p. 3.

98. Yu, J., Chen, Y., Fu, X., Zhou, X., Peng, Y., Shi, L., et al. (2016). Invasive *Fusobacterium* *nucleatum* may play a role in the carcinogenesis of proximal colon cancer through the serrated neoplasia pathway. *Int. J. Cancer*, 139(6), 1318-1326. doi: 10.1002/ijc.30168

99. Scott, A., Alexander, J. L., Marchesi, J., and Kinross, J. M. (2017). Modification of the appendix microbiome in acute appendicitis and colon adenocarcinoma. *Gastroenterol.*, 152(5), S1228.

100. Suehiro, Y., Sakai, K., Nishioka, M., Hashimoto, S., Takami, T., Higaki, S., et al. (2017). Highly sensitive stool DNA testing of *Fusobacterium* *nucleatum* as a marker for detection of colorectal tumours in a Japanese population. *Ann. Clin. Biochem*., 54(1), 86-91. doi: 10.1177/0004563216643970

101. Yamamura, K., Baba, Y., Miyake, K., Nakamura, K., Shigaki, H., Mima, K., et al. (2017). *Fusobacterium* *nucleatum* in gastroenterological cancer: Evaluation of measurement methods using quantitative polymerase chain reaction and a literature review. *Oncol. Lett*., 14(6), 6373-6378. doi: 10.3892/ol.2017.7001

102. Yoon, H., Kim, N., Park, J. H., Kim, Y. S., Lee, J., Kim, H. W., et al. (2017). Comparisons of gut microbiota among healthy control, patients with conventional adenoma, sessile serrated adenoma, and colorectal cancer. *J. Cancer Prev*., 22(2), 108. doi: 10.15430/JCP.2017.22.2.108

103. Yu, J., Feng, Q., Wong, S. H., Zhang, D., Liang, Q., Qin, Y., et al. (2017). Metagenomic analysis of faecal microbiome as a tool towards targeted non-invasive biomarkers for colorectal cancer. *Gut*, 66(1), 70-78. doi: 10.1136/gutjnl-2015-309800

104. Butt, J., Jenab, M., Pawlita, M., Overvad, K., Tjonneland, A., Olsen, A., et al. (2019). Antibody responses to *Fusobacterium* *nucleatum* proteins in prediagnostic blood samples are not associated with risk of developing colorectal cancer. *Cancer Epidemiol. Prev. Biomark*., 28(9), 1552-1555. doi: 10.1158/1055-9965.EPI-19-0313

105. Repass, J. (2018). Replication Study: *Fusobacterium* *nucleatum* infection is prevalent in human colorectal carcinoma. *Elife*, 7, e25801. doi: 10.7554/eLife.25801

106. Russo, E., Bacci, G., Chiellini, C., Fagorzi, C., Niccolai, E., Taddei, A., et al. (2018). Preliminary comparison of oral and intestinal human microbiota in patients with colorectal cancer: a pilot study. *Front. Microbiol*., 8, 2699. doi: 10.3389/fmicb.2017.02699

107. Chen, X. H., Wang, A., Chu, A. N., Gong, Y. H., and Yuan, Y. (2019). Mucosa-associated microbiota in gastric cancer tissues compared with non-cancer tissues. *Front. Microbiol*., 10, 1261. doi: 10.3389/fmicb.2019.01261

108. De Carvalho, A. C., de Mattos Pereira, L., Datorre, J. G., Dos Santos, W., Berardinelli, G. N., Matsushita, M. D. M., et al. (2019). Microbiota profile and impact of *Fusobacterium* *nucleatum* in colorectal cancer patients of Barretos Cancer Hospital. *Front. Oncol*., 9, 813. doi: 10.3389/fonc.2019.00813

109. Kageyama, S., Takeshita, T., Takeuchi, K., Asakawa, M., Matsumi, R., Furuta, M., et al. (2019). Characteristics of the salivary microbiota in patients with various digestive tract cancers. *Front. Microbiol*., 10, 1780. doi: 10.3389/fmicb.2019.01780

110. Saito, K., Koido, S., Odamaki, T., Kajihara, M., Kato, K., Horiuchi, S., et al. (2019). Metagenomic analyses of the gut microbiota associated with colorectal adenoma. *PLoS One*, 14(2), e0212406. doi: 10.1371/journal.pone.0212406

111. Yachida, S., Mizutani, S., Shiroma, H., Shiba, S., Nakajima, T., Sakamoto, T., et al. (2019). Metagenomic and metabolomic analyses reveal distinct stage-specific phenotypes of the gut microbiota in colorectal cancer. *Nat. Med*., 25(6), 968-976. doi: 10.1038/s41591-019-0458-7

112. Alkharaan, H., Lu, L., Gabarrini, G., Halimi, A., Ateeb, Z., Sobkowiak, M. J., et al. (2020). Circulating and salivary antibodies to *Fusobacterium* *nucleatum* are associated with cystic pancreatic neoplasm malignancy. *Front. Immunol*., 11, 2003. doi: 10.3389/fimmu.2020.02003

113. Boehm, E. T., Thon, C., Kupcinskas, J., Steponaitiene, R., Skieceviciene, J., Canbay, A., et al. (2020). *Fusobacterium* *nucleatum* is associated with worse prognosis in Lauren’s diffuse type gastric cancer patients. *Sci. Rep*., 10(1), 1-12. doi: 10.1038/s41598-020-73448-8

114. Gantuya, B., El Serag, H. B., Matsumoto, T., Ajami, N. J., Uchida, T., Oyuntsetseg, K., et al. (2020). Gastric mucosal microbiota in a Mongolian population with gastric cancer and precursor conditions. *Aliment. Pharmacol. Ther*., 51(8), 770-780. doi: 10.1111/apt.15675

115. Kashani, N., Bezmin Abadi, A. T., Rahimi, F., and Forootan, M. (2020). FadA-positive *Fusobacterium* *nucleatum* is prevalent in biopsy specimens of Iranian patients with colorectal cancer. *New Microbes New Infect*., 2020. 34. doi: 10.1016/j.nmni.2020.100651

116. Reynolds, I. S., Thomas, V., O’Connell, E., Fichtner, M., McNamara, D. A., Kay, E. W., et al. (2020). Mucinous adenocarcinoma of the rectum: a whole genome sequencing study. *Front. Oncol*., 10, 1682. doi: 10.3389/fonc.2020.01682

117. Zhang, C., Thakkar, P. V., Sharma, P., Vennelaganti, S., Betel, D., and Shah, M. A. (2019). Abstract 2826: Understanding associations among local microbiome, immune response, and efficacy of immunotherapy in esophageal cancer. *Cancer Res.,* 79(13). doi: 10.1158/1538-7445.AM2019-2826

118. Kawasaki, M., Ikeda, Y., Ikeda, E., Takahashi, M., Tanaka, D., Nakajima, Y., et al. (2021). Oral infectious bacteria in dental plaque and saliva as risk factors in patients with esophageal cancer. *Cancer*, 127(4), 512-519. doi: 10.1002/cncr.33316

119. Kurt, M., and Yumuk, Z. (2021). Diagnostic accuracy of *Fusobacterium* *nucleatum* IgA and IgG ELISA test in colorectal cancer. *Sci. Rep*., 11(1), 1-6. doi: 10.1038/s41598-021-81171-1

120. Pignatelli, P., Iezzi, L., Pennese, M., Raimondi, P., Cichella, A., Bondi, D., et al. (2021). The potential of colonic tumor tissue *Fusobacterium* *nucleatum* to predict staging and its interplay with oral abundance in colon cancer patients. *Cancers*, 13(5), 1032. doi: 10.3390/cancers13051032

121. Eisele, Y., Mallea, P. M., Gigic, B., Stephens, W. Z., Warby, C. A., Buhrke, K., et al. (2021). *Fusobacterium* *nucleatum* and clinicopathologic features of colorectal cancer: results from the ColoCare study. *Clin. Colorectal Cancer*, 20(3), e165-e172. doi: 10.1016/j.clcc.2021.02.007

122. Gethings-Behncke, C., Coleman, H. G., Jordao, H. W., Longley, D. B., Crawford, N., Murray, L. J., et al. (2020). *Fusobacterium* *nucleatum* in the colorectum and its association with cancer risk and survival: a systematic review and meta-analysis. *Cancer Epidemiol. Prev. Biomark*., 29(3), 539-548. doi: 10.1158/1055-9965.EPI-18-1295

123. Janati, A. I., Karp, I., Laprise, C., Sabri, H., and Emami, E. (2020). Detection of *Fusobaterium* *nucleatum* in feces and colorectal mucosa as a risk factor for colorectal cancer: a systematic review and meta-analysis. *Syst. Rev*., 9(1), 1-15. doi: 10.1186/s13643-020-01526-z

128. Ahn, J., Segers, S., and Hayes, R. B. (2012). Periodontal disease, *Porphyromonas* *gingivalis* serum antibody levels and orodigestive cancer mortality. *Carcinogenesis*, 33(5), 1055-1058. doi: 10.1093/carcin/bgs112

129. Fan, X., Alekseyenko, A. V., Wu, J., Peters, B. A., Jacobs, E. J., Gapstur, S. M., et al. (2018). Human oral microbiome and prospective risk for pancreatic cancer: a population-based nested case-control study. *Gut*, 67(1), 120-127. doi: 10.1136/gutjnl-2016-312580

130. Gao, S., Li, S., Ma, Z., Liang, S., Shan, T., Zhang, M., et al. (2016). Presence of *Porphyromonas* *gingivalis* in esophagus and its association with the clinicopathological characteristics and survival in patients with esophageal cancer. *Infect. Agents Cancer*, 11(1), 1-9. doi: 10.1186/s13027-016-0049-x

131. Gao, S. G., Yang, J. Q., Ma, Z. K., Yuan, X., Zhao, C., Wang, G. C., et al. (2018). Preoperative serum immunoglobulin G and A antibodies to *Porphyromonas* *gingivalis* are potential serum biomarkers for the diagnosis and prognosis of esophageal squamous cell carcinoma. *BMC Cancer*, 18(1), 1-8. doi: 10.1186/s12885-017-3905-1

132. Michaud, D. S., Izard, J., Wilhelm-Benartzi, C. S., You, D. H., Grote, V. A., Tjønneland, A., et al. (2013). Plasma antibodies to oral bacteria and risk of pancreatic cancer in a large European prospective cohort study. *Gut*, 62(12), 1764-1770. doi: 10.1136/gutjnl-2012-303006

133. Peters, B. A., Wu, J., Pei, Z., Yang, L., Purdue, M. P., Freedman, N. D., et al. (2017). Oral microbiome composition reflects prospective risk for esophageal cancers. *Cancer Res*., 77(23), 6777-6787. doi: 10.1158/0008-5472.CAN-17-1296

134. Wang, X., Jia, Y., Wen, L., Mu, W., Wu, X., Liu, T., et al. (2021). *Porphyromonas* *gingivalis* promotes colorectal carcinoma by activating the hematopoietic NLRP3 inflammasome. *Cancer Res*., 81(10), 2745-2759. doi: 10.1158/0008-5472.CAN-20-3827

135. Sinha, R., Ahn, J., Sampson, J. N., Shi, J., Yu, G., Xiong, X., et al. (2016). Fecal microbiota, fecal metabolome, and colorectal cancer interrelations. *PloS One*, 11(3), e0152126. doi: 10.1371/journal.pone.0152126

136. Scanu, T., Spaapen, R. M., Bakker, J. M., Pratap, C. B., Wu, L. E., Hofland, I., et al. (2015). *Salmonella* manipulation of host signaling pathways provokes cellular transformation associated with gallbladder carcinoma. Cell Host Microbe, 17(6), 763-774.

137. Chang, R., Wei, J. C. C., Lin, M. C., and Hung, Y. M. (2021). The potential role of nontyphoidal salmonellosis in gastric cancer: a nationwide matched cohort study. *Gastric Cancer*, 24(2), 292-301. doi: s10120-020-01132-x

138. Duijster, J. W., Hansen, J. V., Franz, E., Neefjes, J. J., Frisch, M., Mughini-Gras, L., et al. (2021). Association between *Salmonella* infection and colon cancer: a nationwide registry-based cohort study. *Epidemiol. Infect*., 149, E56. doi: 10.1017/S0950268821000285

139. Iyer, P., Barreto, S. G., Sahoo, B., Chandrani, P., Ramadwar, M. R., Shrikhande, S. V., et al. (2016). Non-typhoidal *Salmonella* DNA traces in gallbladder cancer. *Infect. Agents Cancer*, 11(1), 1-4. doi: 10.1186/s13027-016-0057-x

140. Kato, I., Boleij, A., Kortman, G. A., Roelofs, R., Djuric, Z., Severson, R. K., et al. (2013). Partial associations of dietary iron, smoking and intestinal bacteria with colorectal cancer risk. *Nutr. Cancer*, 65(2), 169-177. doi: 10.1080/01635581.2013.748922

141. Lu, R., Bosland, M., Xia, Y., Zhang, Y. G., Kato, I., and Sun, J. (2017). Presence of *Salmonella* AvrA in colorectal tumor and its precursor lesions in mouse intestine and human specimens. *Oncotarget*, 8(33), 55104. doi: 10.18632/oncotarget.19052

142. Mughini-Gras, L., Schaapveld, M., Kramers, J., Mooij, S., Neefjes-Borst, E. A., Pelt, W. V., et al. (2018). Increased colon cancer risk after severe *Salmonella* infection. *PloS One*, 13(1), e0189721. doi: 10.1371/journal.pone.0189721

145. Capoor, M. R., Nair, D., Khanna, G., Krishna, S. V., Chintamani, M. S., and Aggarwal, P. (2008). Microflora of bile aspirates in patients with acute cholecystitis with or without cholelithiasis: a tropical experience. *Braz. J. Infect. Dis.*, 12, 222-225. doi: 10.1590/S1413-86702008000300012

146. Caygill, C. P., Hill, M. J., Braddick, M., and Sharp, J. C. (1994). Cancer mortality in chronic typhoid and paratyphoid carriers. *Lancet*, 343(8889), 83-84. doi: 10.1016/S0140-6736(94)90816-8

147. Csendes, A., Becerra, M., Burdiles, P., Demian, I., Bancalari, K., and Csendes, P. (1994). Bacteriological studies of bile from the gallbladder in patients with carcinoma of the gallbladder, cholelithiasis, common bile duct stones and no gallstones disease. *Eur. J. Surg*., 160(6-7), 363-367.

148. Dutta, U., Garg, P. K., Kumar, R., and Tandon, R. K. (2000). Typhoid carriers among patients with gallstones are at increased risk for carcinoma of the gallbladder. *Am. J. Gastroenterol*., 95(3), 784-787. doi: 10.1016/S0002-9270(99)00919-3

149. Hazrah, P., Oahn, K. T. H., Tewari, M., Pandey, A. K., Kumar, K., Mohapatra, T. M., et al. (2004). The frequency of live bacteria in gallstones. HPB, 6(1), 28-32. doi: 10.1080/13651820310025192

150. Mellemgaard, A. and K. Gaarslev, Risk of hepatobiliary cancer in carriers of *Salmonella* typhi*. J. Nat. Cancer Institute*, 1988. 80(4): p. 288. doi: 10.1093/jnci/80.4.288

151. Nath, G., Singh, H., and Shukla, V. K. (1997). Chronic typhoid carriage and carcinoma of the gallbladder. *Eur. J. Cancer Prev*., 6(6), 557-559. doi: 10.1097/00008469-199712000-00011

152. Nath, G., Singh, Y. K., Kumar, K., Gulati, A. K., Shukla, V. K., Khanna, A. K., et al. (2008). Association of carcinoma of the gallbladder with typhoid carriage in a typhoid endemic area using nested PCR. *J. Infect. Dev. Ctries*., 2(04), 302-307. doi: 10.3855/jidc.226

153. Pandey, M., and Shukla, V. K. (2003). Lifestyle, parity, menstrual and reproductive factors and risk of gallbladder cancer. *Eur. J. Cancer Prev*., 12(4), 269-272.

154. Roa, I., Ibacache, G., Carvallo, J., Melo, A., Araya, J., De Aretxabala, X., et al. (1999). Microbiological study of gallbladder bile in a high risk zone for gallbladder cancer. *Rev. Med.Chile*, 127(9), 1049-1055.

155. Safaeian, M., Gao, Y. T., Sakoda, L. C., Quraishi, S. M., Rashid, A., Wang, B. S., et al. (2011). Chronic typhoid infection and the risk of biliary tract cancer and stones in Shanghai, China. *Infect. Agents Cancer*, 6(1), 1-3. doi: 10.1186/1750-9378-6-6

156. Serra, I., Yamamoto, M., Calvo, A., Cavada, G., Báez, S., Endoh, K., et al. (2002). Association of chili pepper consumption, low socioeconomic status and longstanding gallstones with gallbladder cancer in a Chilean population. *Int. J. Cancer*, 102(4), 407-411. doi: 10.1002/ijc.10716

157. Sharma, V., Chauhan, V. S., Nath, G., Kumar, A., and Shukla, V. K. (2007). Role of bile bacteria in gallbladder carcinoma. *Hepato-gastroenterology*, 54(78), 1622-1625.

158. Shukla, V. K., Singh, H., Pandey, M., Upadhyay, S. K., and Nath, G. (2000). Carcinoma of the gallbladder—is it a sequel of typhoid?. *Dig. Dis. Sci*., 45(5), 900-903. doi: 10.1023/A:1005564822630

159. Singh, H., Pandey, M., amd Shukla, V. K. (1996). *Salmonella* carrier state, chronic bacterial infection and gallbladder carcinogenesis. *Eur. J. Cancer Prev*., 5(2), 144.

160. Strom, B. L., Soloway, R. D., Rios‐Dalenz, J. L., Rodriguez‐Martinez, H. A., West, S. L., Kinman, J. L., et al. (1995). Risk factors for gallbladder cancer. An international collaborative case–control study. *Cancer*, 76(10), 1747-1756. doi: 10.1002/1097-0142(19951115)76:10<1747::AID-CNCR2820761011>3.0.CO;2-L

161. Tewari, M., Mishra, R. R., and Shukla, H. S. (2010). *Salmonella* typhi and gallbladder cancer: report from an endemic region. *Hepatobiliary Pancreat. Dis. Int*., 9(5), 524-530.

162. Vaishnavi, C., Kochhar, R., Singh, G., Kumar, S., Singh, S., and Singh, K. (2005). Epidemiology of typhoid carriers among blood donors and patients with biliary, gastrointestinal and other related diseases. *Microbiol. Immunol*., 49(2), 107-112. doi: 10.1111/j.1348-0421.2005.tb03709.x

163. Welton, J., Marr, J., and Friedman, S. (1979). Association between hepatobiliary cancer and typhoid carrier state. *Lancet*, 313(8120), 791-794. doi: 10.1016/S0140-6736(79)91315-1

164. Yagyu, K., Lin, Y., Obata, Y., Kikuchi, S., Ishibashi, T., Kurosawa, M., et al. (2004). Bowel movement frequency, medical history and the risk of gallbladder cancer death: a cohort study in Japan. *Cancer Sci*., 95(8), 674-678. doi: 10.1111/j.1349-7006.2004.tb03328.x

165. Nagaraja, V., and Eslick, G. D. (2014). Systematic review with meta‐analysis: the relationship between chronic *Salmonella* typhi carrier status and gall‐bladder cancer. *Aliment. Pharmacol. Ther*., 39(8), 745-750. doi: 10.1111/apt.12655

166. Koshiol, J., Wozniak, A., Cook, P., Adaniel, C., Acevedo, J., Azócar, L., et al. (2016). *Salmonella* enterica serovar Typhi and gallbladder cancer: a case–control study and meta‐analysis. *Cancer Med*., 5(11), 3310-3235. doi: 10.1002/cam4.915

173. Qiu, D. C., Hubbard, A. E., Zhong, B., Zhang, Y., and Spear, R. C. (2005). A matched, case–control study of the association between *Schistosoma* *japonicum* and liver and colon cancers, in rural China. *Ann. Trop. Med. Parasitol*., 99(1), 47-52. doi: 10.1179/136485905X19883

174. Xu, Z., and Su, D. L. (1984). Schistosoma japonicum and colorectal cancer: an epidemiological study in the People's Republic of China. *Int. J. Cancer*, 34(3), 315-318. doi: 10.1002/ijc.2910340305

175. En-Sheng, Z., Cancer of the colon and schistosomiasis. 1981, SAGE Publications Sage UK: London, England.

176. Iida, F., Iida, R., Kamijo, H., Takaso, K., Miyazaki, Y., Funabashi, W., et al. (1999). Chronic Japanese schistosomiasis and hepatocellular carcinoma: ten years of follow-up in Yamanashi Prefecture, Japan. *Bull. World Health Organ*., 77(7), 573.

177. Madbouly, K. M., Senagore, A. J., Mukerjee, A., Hussien, A. M., Shehata, M. A., Navine, P., et al. (2007). Colorectal cancer in a population with endemic *Schistosoma* *mansoni*: is this an at-risk population?. *Int. J. Colorectal Dis*., 22(2), 175-181. doi: 10.1007/s00384-006-0144-3

178. Toda, K. S., Kikuchi, L., Chagas, A. L., Tanigawa, R. Y., Paranaguá-Vezozzo, D. C., Pfiffer, T., et al. (2015). Hepatocellular carcinoma related to *Schistosoma* *mansoni* infection: case series and literature review. *J. Clin. Transl. Hepatol*., 3(4), 260. doi: 10.14218/JCTH.2015.00027

182. Beck, M., Frodl, R., and Funke, G. (2008). Comprehensive study of strains previously designated *Streptococcus* *bovis* consecutively isolated from human blood cultures and emended description of *Streptococcus* *gallolyticus* and *Streptococcus* *infantarius* subsp. *coli*. *J. Clin. Microbiol*., 46(9), 2966-2972. doi: 10.1128/JCM.00078-08

183. Corredoira, J. C., Alonso, M. P., Garcia, J. F., Casariego, E., Coira, A., Rodriguez, A., et al. (2005). Clinical characteristics and significance of *Streptococcus* *salivarius* bacteremia and *Streptococcus* *bovis* bacteremia: a prospective 16-year study. *Eur. J. Clin. Microbiol. Infect. Dis*., 24(4), 250-255. doi: 10.1007/s10096-005-1314-x

184. Gonzlez-Quintela, A., Martınez-Rey, C., Castroagudın, J. F., Rajo-Iglesias, M. C., and Domınguez-Santalla, M. J. (2001). Prevalence of liver disease in patients with *Streptococcus* *bovis* bacteraemia. *J. Infect*., 42(2), 116-119. doi: 10.1053/jinf.2001.0799

185. Jean, S. S., Teng, L. J., Hsueh, P. R., Ho, S. W., and Luh, K. T. (2004). Bacteremic *Streptococcus* *bovis* infections at a university hospital, 1992-2001. *J. Formos. Med. Assoc*., 103(2), 118-123.

186. Murray, H. W., and Roberts, R. B. (1978). *Streptococcus* *bovis* bacteremia and underlying gastrointestinal disease. *Arch. Int. Med*., 138(7), 1097-1099. doi: 10.1001/archinte.1978.03630320037013

187. Ruoff, K. L., Miller, S. I., Garner, C. V., Ferraro, M. J., and Calderwood, S. B. (1989). Bacteremia with *Streptococcus* *bovis* and *Streptococcus* *salivarius*: clinical correlates of more accurate identification of isolates. *J. Clin. Microbiol.*, 27(2), 305-308. doi: 10.1128/jcm.27.2.305-308.1989

188. Zarkin, B. A., Lillemoe, K. D., Cameron, J. L., Effron, P. N., Magnuson, T. H., and Pitt, H. A. (1990). The triad of *Streptococcus* *bovis* bacteremia, colonic pathology, and liver disease. *Ann. Surg*., 211(6), 786. doi: 10.1097/00000658-199006000-00019

189. Boleij, A., Roelofs, R., Schaeps, R. M., Schülin, T., Glaser, P., Swinkels, D. W., et al. (2010). Increased exposure to bacterial antigen RpL7/L12 in early stage colorectal cancer patients. *Cancer*, 116(17), 4014-4022. doi: 10.1002/cncr.25212

190. Corredoira, J., Alonso, M. P., Coira, A., and Varela, J. (2008). Association between *Streptococcus* *infantarius* (formerly *S.* *bovis* II/1) bacteremia and noncolonic cancer. *J. Clin. Microbiol.*, 46(4), 1570-1570. doi: 10.1128/JCM.00129-08

191. Fernández-Ruiz, M., Villar-Silva, J., Llenas-García, J., Caurcel-Díaz, L., Vila-Santos, J., Sanz-Sanz, F., et al. (2010). *Streptococcus* *bovis* bacteraemia revisited: clinical and microbiological correlates in a contemporary series of 59 patients. *J. Infect*., 61(4), 307-313. doi: 10.1016/j.jinf.2010.07.007

192. Vaska, V. L., and Faoagali, J. L. (2009). *Streptococcus* *bovis* bacteraemia: identification within organism complex and association with endocarditis and colonic malignancy. *Pathology*, 41(2), 183-186. doi: 10.1080/00313020802436816

193. Boleij, A., Roelofs, R., Danne, C., Bellais, S., Dramsi, S., Kato, I., et al. (2012). Selective antibody response to *Streptococcus* *gallolyticus* pilus proteins in colorectal cancer patients. *Cancer Prev. Res*., 5(2), 260-265. doi: 10.1158/1940-6207.CAPR-11-0321

194. Garza‐González, E., Ríos, M., Bosques‐Padilla, F. J., Francois, F., Cho, I., González, G. M., et al. (2012). Immune response against *Streptococcus* *gallolyticus* in patients with adenomatous polyps in colon. *Int. J. Cancer*, 131(10), 2294-2299. doi: 10.1002/ijc.27511

195. Zammit, S. C., Azzopardi, N., and Ellul, P. (2014). *Streptococcus* *gallolyticus* bacteraemia in hepatobiliary–pancreatic and colonic pathologies. *QJM: Int. J. Med*., 107(5), 355-361. doi: 10.1093/qjmed/hct261

196. Butt, J., Romero‐Hernández, B., Pérez‐Gómez, B., Willhauck‐Fleckenstein, M., Holzinger, D., Martin, V., et al. (2016). Association of *Streptococcus* *gallolyticus* subspecies *gallolyticus* with colorectal cancer: serological evidence. *Int. J. Cancer,* 138(7), 1670-1679. doi: 10.1002/ijc.29914

197. Butt, J., Werner, S., Willhauck‐Fleckenstein, M., Michel, A., Waterboer, T., Zörnig, I., et al. (2017). Serology of *Streptococcus* *gallolyticus* subspecies *gallolyticus* and its association with colorectal cancer and precursors. *Int. J. Cancer*, 141(5), 897-904. doi: 10.1002/ijc.30765

198. Paritsky, M., Pastukh, N., Brodsky, D., Isakovich, N., and Peretz, A. (2015). Association of *Streptococcus* *bovis* presence in colonic content with advanced colonic lesion. *World J. Gastroenterol*., 21(18), 5663. doi: 10.3748/wjg.v21.i18.5663

199. Butt, J., Blot, W. J., Teras, L. R., Visvanathan, K., Le Marchand, L., Haiman, C. A., et al. (2018). Antibody responses to *Streptococcus* *Gallolyticus* subspecies *Gallolyticus* proteins in a large prospective colorectal cancer cohort consortium. *Cancer Epidemiol. Prev. Biomark*., 27(10), 1186-1194. doi: 10.1158/1055-9965.EPI-18-0249

200. Butt, J., Jenab, M., Willhauck‐Fleckenstein, M., Michel, A., Pawlita, M., Kyrø, C., et al. (2018). Prospective evaluation of antibody response to *Streptococcus* *gallolyticus* and risk of colorectal cancer. *Int. J. Cancer*, 143(2), 245-252. doi: 10.1002/ijc.31283

201. Guven, D. C., Dizdar, O., Alp, A., Akdogan Kittana, F. N., Karakoc, D., Hamaloglu, E., et al. (2018). Analysis of *Fusobacterium* *nucleatum*, *Streptococcus* *gallolyticus* and *Porphyromonas* *gingivalis* in saliva in colorectal cancer patients and healthy controls. *J. Clin. Oncol.,* 36(15). doi: 10.1200/JCO.2018.36.15_suppl.e15617

202. Kale, P., Khillan, V., and Sarin, S. K. (2018). Novel association of *Streptococcus* *gallolyticus* subspecies *pasteurianus* and hepatocelluar carcinoma: opening new frontiers. *Scand. J. Gastroenterol*., 53(10-11), 1354-1357. doi: 10.1080/00365521.2018.1511826

203. Sheikh, A. F., Zadeh, A. R. M., Saki, M., Khani, P., Hashemi, S. J., Zadeh, S. S., et al. (2020). Detection of *Streptococcus* *gallolyticus* in colorectal cancer and inflammatory bowel disease patients compared to control group in southwest of Iran. *Mol. Biol. Rep*., 47(11), 8361-8365. doi: 10.1007/s11033-020-05807-7

204. Boleij, A., van Gelder, M. M., Swinkels, D. W., and Tjalsma, H. (2011). Clinical importance of *Streptococcus* *gallolyticus* infection among colorectal cancer patients: systematic review and meta-analysis. *Clin. Infect. Dis*., 53(9), 870-878. doi: 10.1093/cid/cir609

205. Krishnan, S., and Eslick, G. D. (2014). *Streptococcus* *bovis* infection and colorectal neoplasia: a meta‐analysis. *Colorectal Dis*., 16(9), 672-680. doi: 10.1111/codi.12662

210. Tanaka, T., Hirata, T., Parrott, G., Higashiarakawa, M., Kinjo, T., Kinjo, T., et al. (2016). Relationship among *Strongyloides* *stercoralis* infection, human T-cell lymphotropic virus type 1 infection, and cancer: a 24-year cohort inpatient study in Okinawa, Japan. *Am. J. Trop. Med. Hyg*., 94(2), 365. doi: 10.4269/ajtmh.15-0556

211. Hirata, T., Kishimoto, K., Kinjo, N., Hokama, A., Kinjo, F., and Fujita, J. (2007). Association between *Strongyloides* *stercoralis* infection and biliary tract cancer. *Parasitol. Res.*, 101(5), 1345-1348. doi: 10.1007/s00436-007-0648-y
